# The anti-aging potential of antihypertensive peptides of *Pariset*, a dataset of algal peptides

**DOI:** 10.3389/fragi.2025.1618082

**Published:** 2025-07-30

**Authors:** Isaac Karimi, Parisa Olfati, Layth Jasim Mohammed, Jawad Kadhim Tarrad, Ahmed M. Amshawee, Maryam A. Hussain, Helgi B. Schiöth

**Affiliations:** ^1^ Laboratory for Computational Physiology, Department of Biology, Faculty of Science, Razi University, Kermanshah, Iran; ^2^ Department of Microbiology, College of Medicine, Babylon University, Hilla City, Iraq; ^3^ Department of Radiology, University of Hilla, Babylon, Iraq; ^4^ Babylon Technical Institute, AL-Furat Al-Awsat Technical University, Babylon, Iraq; ^5^ Department of Surgical Sciences, Functional Pharmacology and Neuroscience, Uppsala University, Uppsala, Sweden

**Keywords:** *Pariset* dataset, algal peptides, hypertension, renoprotection, senescence

## Abstract

**Introduction:**

Cellular senescence drives aging and disease by promoting inflammation and tissue dysfunction. The kidneys, highly susceptible to aging, worsen with hypertension, increasing chronic disease risk. Managing blood pressure with angiotensin-converting enzyme (ACE) inhibitors and natural bioactive peptides helps maintain kidney health. This study explores a kidney-associated aging network and algal peptides with renoprotective and anti-aging effects.

**Methods:**

Senescence-associated genes from Human Ageing Genomic Resources (HAGR) were used to construct and analyze a protein-protein interaction (PPI) network, refining a kidney-related subset ACE, angiotensin II Receptor Type 1 (AGTR1), and angiotensin II Receptor Type 2 (AGTR2). Algal antihypertensive peptides were filtered out of the laboratory dataset of algal peptides, *Pariset*, and assessed for allergenicity, antigenicity, toxicity, and anti-aging potential via sequence similarity searches. Selected peptides were prepared for molecular docking, tested against kidney-aging targets, and visualized.

**Results:**

A senescence-associated PPI network revealed key aging-related proteins—IL1R, CD4, FN1, STAT3, CD45, APOE, CD44, ITGAM. CD8A, CD68, CDH1, ACE, AGTR1, and AGTR2—linked to inflammation, immunity, and fibrosis. Screening identified 54 antihypertensive peptides, among which seven were predicted to be non-allergenic and non-antigenic peptides, while six out of them exhibited anti-aging properties. KTFPY and others exhibited strong binding to ACE and kidney-aging proteins, suggesting therapeutic benefits.

**Discussion:**

The senescence-associated PPI network reveals potentially important aging-related proteins affecting kidney health. Algal peptides, particularly KTFPY, VYRT, PGDTY, PVAFN, and MTFF, exhibit strong ACE binding, suggesting potential antihypertensive and anti-aging benefits. CD68 expressed reliable binding affinities with small-molecule ACE inhibitors, and it indicated the repurposing potential of these drugs for aging-associated conditions. These computational results highlight the potential of peptide-based therapies in addressing age-related kidney dysfunction, and warrant further experimental investigations.

## 1 Introduction

Cellular senescence refers to a state where cells cease to divide but remain metabolically active, often secreting harmful molecules known as the senescence-associated secretory phenotype [SASP; (Kirkland and Tchkonia, 2017)]. These secretions contribute to inflammation, tissue dysfunction, and other hallmarks of aging. Targeting senescent cells has become a promising therapeutic strategy, leading to the development of senolytic drugs, which selectively abolish these cells, and senomorphic agents, which modulate their harmful effects (Xu et al., 2018). Key pathways for intervention include anti-apoptotic mechanisms like senescent cell anti-apoptotic pathways and SASP-associated signaling. Compounds such as dasatinib and quercetin, fisetin, and navitoclax have shown preclinical success in reducing senescent cell burden, mitigating age-related disorders, and improving healthspan (Krzystyniak et al., 2022). Furthermore, studies on heat shock protein 90 (hsp90) inhibitors and Janus kinase (JAK) inhibitors highlight the potential to suppress SASP-induced inflammation, further expanding the arsenal against aging-related conditions (Childs et al., 2015). Therefore, delving deeper into molecular aspects of aging may be a helpful and hopeful strategy for treating chronic diseases, which act as aging accelerators.

The kidneys are often the first organs to exhibit aging symptoms due to their high workload. Kidney aging is a natural process marked by a gradual decline in function, includes reduced filtration efficiency and structural alterations. Hypertension, or high blood pressure, can exacerbate this decline by placing additional strain on the kidneys, thereby accelerating the progression to chronic kidney disease (CKD). Recent studies, such as a 10-year population-based analysis, have highlighted the interplay between aging, hypertension, and declining kidney function (Jaques et al., 2022). In conclusion, effectively managing hypertension through lifestyle modifications and appropriate medications is vital to slowing this decline and maintaining kidney health as individuals age (Ren et al., 2024).

Drug management of hypertension is a cornerstone of controlling hypertension and preventing complications such as heart disease, stroke, and kidney damage. In this context, angiotensin-converting enzyme (ACE) inhibitors (ACEIs), such as lisinopril and captopril, and putative biopeptides are medications used to manage hypertension, heart failure, and kidney diseases like diabetic nephropathy (Ahmad et al., 2023). They work by blocking the ACE, thereby reducing the production of angiotensin II, a hormone that constricts blood vessels and elevates blood pressure. By inhibiting this process, ACEIs help relax and widen blood vessels, improving blood flow and reducing the strain on the heart and kidneys. In this regard, ACEIs are effective and well-tolerated, though some patients may experience side effects like dry cough, elevated potassium levels, or dizziness, which require monitoring by healthcare professionals. The bioactive peptides, often derived from natural sources like milk proteins, plant proteins, or meat hydrolysates, work by blocking the activity of ACE (Ahmad et al., 2023). For example, peptides obtained from fermented milk and porcine liver and placenta or enzymatically hydrolyzed plant proteins have demonstrated ACE-inhibitory activity *in vitro* and *in vivo* (Daskaya-Dikmen et al., 2017; Pearman et al., 2025; Aslam et al., 2019). Therefore, peptides that inhibit ACE are gaining attention for their potential in managing hypertension.

Algae are excellent sources of peptides due to their sustainable cultivation, rapid growth rates, and rich diversity of proteins, which can be enzymatically hydrolyzed to yield bioactive compounds with nutritional and pharmaceutical benefits. Interestingly, algal peptides have significant potential as natural inhibitors of ACE, making them promising candidates for managing hypertension. These bioactive peptides are often derived from the enzymatic hydrolysis of various algal proteins. For instance, peptides extracted from the marine red alga *Gracilariopsis lemaneiformis* have demonstrated high ACE inhibitory activity, with specific sequences like Gln-Val-Glu-Tyr (QVEY) identified as potent inhibitors (Cao et al., 2017). Similarly, peptides derived from the red alga *Acrochaetium* sp. possess non-competitive ACE inhibitory activity, highlighting their therapeutic potential (Windarto et al., 2022). These peptides typically exhibit low molecular weight and specific amino acid sequences that enhance their binding affinity (BA; kcal/mol) to ACE, thereby reducing its activity. In this computational study, we first aimed to construct an aging protein-protein interaction (PPI) network along with its kidney-associated aging subnetwork. Subsequently, the antihypertensive algal peptides filtered out from our laboratory dataset, *Pariset*, were used to identify their antiaging potential in the kidney PPI aging subnetwork.

## 2 Materials and methods

### 2.1 Protein-protein interaction network construction and analysis

The entire set of senescence-associated genes has been downloaded from the Human Ageing Genomic Resources (HAGR; https://genomics.senescence.info/). HAGR is a collection of databases and tools designed to study the genetics of human aging using modern approaches such as functional genomics, network analyses, systems biology, and evolutionary analyses. The downloaded genes were matched with their equivalent protein names in UniProt launched at https://www.uniprot.org and submitted to the STRING database, accessible at https://string-db.org/cgi/network, to construct a protein-protein interaction (PPI) network. The PPI network was robotically submitted to Cytoscape *ver*. 3.10.2 (Shannon et al., 2003) and initially analyzed with the installed StringApp (Doncheva et al., 2018), and its summary statistics were reported. Moreover, this network was analyzed with the cytoHubba plugin launched at http://hub.iis.sinica.edu.tw/cytohubba/ to explore hub genes and special features of the network (Chin et al., 2014). PPI subnetwork were pooled and submitted to the STRING database to decipher their interactome and the STITCH database (Kuhn et al., 2007) to explore their protein-chemical network. Functional enrichment analyses of hub genes were performed using Metascape launched at http://metascape.org/gp/index.html#/main/step1, applying a cutoff value of P < 0.05 (Zhou et al., 2019).

### 2.2 Allergenicity, toxicity, and antigenicity of algal antihypertensive peptide

The antihypertensive peptides were filtered out from the *Pariset*, a dataset curated from PubMed and updated continuously, and their allergenicities, toxicities, and antigenicities were assessed using the online tool AllerCatPro *ver*. 2.0 (Maurer-Stroh et al., 2019; Nguyen et al., 2022) launched at https://allercatpro.bii.a-star.edu.sg/, ToxinPred launched at http://crdd.osdd.net/raghava/toxinpred (Gupta et al., 2013), and Vaxijen *ver.* 2.0 (Doytchinova and Flower, 2007) launched at https://www.ddg-pharmfac.net/vaxijen/VaxiJen/VaxiJen.html.

### 2.3 Anti-aging potential of algal antihypertensive peptide

The Smith-Waterman algorithm was employed to examine the sequence similarities of selected peptides with anti-aging peptides using FASTA software *ver*. 3.8. This search was performed in the AagingBase database (Kunjulakshmi et al., 2024) launched at https://project.iith.ac.in/cgntlab/aagingbase/index.php, which includes anti-aging peptides to show the biological ontology of *Pariset*. The z-score and Smith-Waterman score of the peptides were exported into an Excel file.

### 2.4 Molecular docking of selected algal antihypertensive peptides and benchmark ACEIs with aging targets

The selected peptide sequences were converted into the 3D Protein Data Bank (PDB; http://www.RCSB.org) structure format using Probuilder *on-line* server launched at https://www.ddl.unimi.it/vegaol/probuilder.htm. Each PDB file was provided to the UCSF Chimera software *ver*. 1.8.1 was launched at http://www.rbvi.ucsf.edu/chimera for preparation and hydrogenated using the DOC PREP algorithm, and saved (Pettersen et al., 2004). All protein targets involved in kidney aging were searched for the PDB structures and downloaded from RCSB PDB. The structures were processed to remove co-crystallized ligands, cofactors, and water using the Molegro Virtual Docker (Thomsen and Christensen, 2006). After completing these steps and addressing the warnings, the cleaned receptor was saved in PDB format. Molecular docking was performed using scripts in the Hex *ver*. 8.0.0 software (Macindoe et al., 2010). In this case, using Notepad, the docking commands were written and executed from the Macro section. Examples of commands that presented in Supplementary Figure S1 of the Supplementary File S1 were provided. Each docked structure was downloaded separately in PDB format, and the docking scores were entered into an Excel file. Specifically, we used a grid resolution of 0.6 Å, with shape plus electrostatics correlation type, and an angular step size of 7.5°. Both the receptor and ligand were treated as rigid bodies, as Hex does not support flexible docking or conformational changes during the simulation. Additionally, we acknowledge the inherent limitations of rigid docking approaches, including the lack of solvent modeling, the absence of conformational flexibility, and the fact that docking provides only a computational estimate of BA. PyRx software *ver*. 0.8 was launched at https://pyrx.software.informer.com/0.8/ and employed to dock benchmark ACEIs onto the protein targets using VINA WIZARD. In this regard, the crystal structure of the target proteins was obtained from the Protein Data Bank (PDB; http://www.RCSB.org) and prepared using Molegro Virtual Docker (*vide supra*) and UCSF Chimera *ver*. 1.8.1 (http://www.rbvi.ucsf.edu/chimera). The structures of the benchmark ACEIs were drawn in GaussView 5.0 and hydrogenated and charged using UCSF Chimera. The BA values were computed, and a more negative value indicates a better binding and orientation, as submitted to the interfacial graphic software. Then, the docking results file was input into the LigPlot^+^ software, and the two-dimensional images of the dockings were saved for dissecting the interactions (Laskowski and Swindells, 2011).

## 3 Results

### 3.1 The protein-protein interaction networks

**FIGURE 1:**
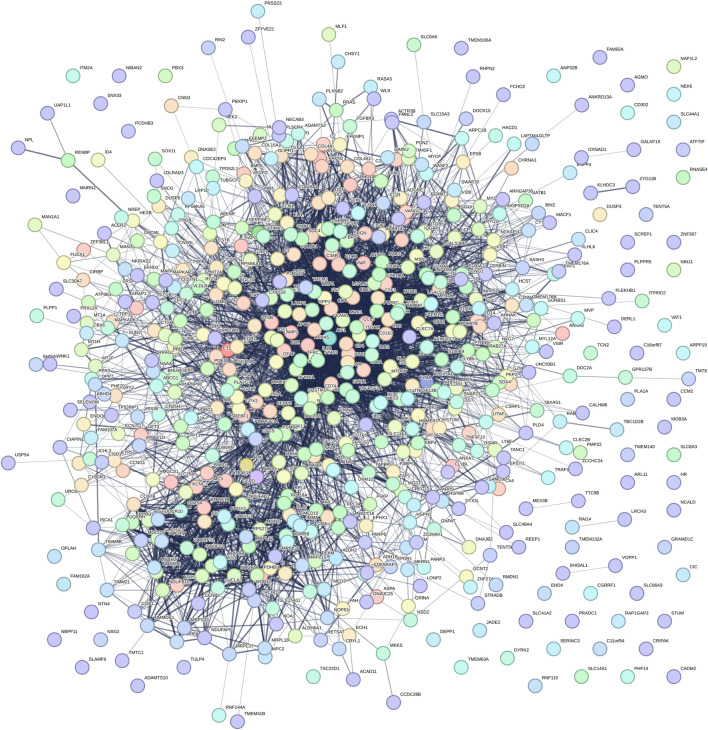
The protein-protein interaction (PPI) networks of genes involving in aging process. PPI network was constructed and analyzed using STRING database, accessible at https://string-db.org/cgi/network. Spheres represent the nodes, while lines or edges indicate the interactions between them.

The senescence-associated PPI was presented in Figure 1. In this context, the summary statistics of this network were as follows: number of nodes: 607; number of edges: 5675; average number of neighbors: 21.276; network diameter: 8; network radius: 4; characteristic path length: 2.805; clustering coefficient: 0.402; network density: 0.040; network heterogeneity: 1.127; network centralization: 0.239; connected components: 70; and analysis time (sec): 0.236. This network was significantly more enriched with PPI enrichment p-value of <1.0e^−16,^ and such an enrichment indicates that the proteins are at least partially biologically connected, as a group, and need to be dissected. The ontological evaluation of this network at biological, molecular, and cellular levels showed that inflammatory responses, cell adhesion molecule binding, and secretory granule lumen were ontological terms for aging with the highest signal rates, respectively (data not shown). The top-ten high-degree nodes of the PPI network computed with cytoHubba and their potential relevance to the human ageing process were reported here (Table 1). The physiopharmacological significance of nodes was presented in Table 1. For example, the interactions of selected antiaging peptides with IL-1RI were pursued since this protein is one of the top players of organismal aging and renal aging (Table 1).

**Table 1:** Common features of the protein-protein interaction (PPI) network constructed using the top-ten ranked nodes of the ageing PPI network and the top-five nodes of the kidney-associated ageing PPI subnetwork**.**

HGNC symbol	Degree scores of aging PPI network/kidney subnetwork	Expression during ageing/longevity association	Known involvement in renal aging and function	Agonist/antagonist/inhibitor	PDB ID
IL1B	148/28	Overexpressed/No significant	Yes	Anakinra(IL-1RA;small molecule)	IL1B (1T4Q) IL1R (1G0Y)
CD4	122/-	Overexpressed	Yes		1CDJ
FN1	116/31	Overexpressed/Pro-longevity in mice	Yes	pUR4 (small peptide)	3M7P
STAT3	116/27	Overexpressed/Pro-longevity in flies	Yes	S3I-201, Stattic (small molecule)	4ZIA
CD45	114/-	Overexpressed	Yes	2-[(4-acetylphenyl) amino]−3-chloronaphthoquinone (small molecule)	1YGU
APOE	110/23		Yes	Epigallocatechin gallate (small molecule)	1B68
CD44	108/26	Overexpressed	Yes	KPSSPPEE (small peptide)	1UUH
ITGAM	105/-	Overexpressed	No	LL-37 (peptide)	1NA5
CD8A	99/-	Overexpressed	No		1CD8
CD68	98/-	Overexpressed	No	P39 (small peptide)	P34810
CDH1	77/24	Overexpressed	Yes		2O72
ACE	-/15		Yes	Angiotensinogen (substrate); angiotensin (product; antagonist) (small peptide)	1O8A
AGTR1	-/8		Yes	Angiotensin (agonist; small peptide)	7F6G
AGTR2	-/5		Yes	Angiotensin (agonist; small peptide)	7JNI

Note: HGNC: Human Gene Nomenclature Committee (HGNC); PPI: Protein-protein interactions (PPIs); PDB ID: Protein Data Bank (PDB); IL1B: Interleukin 1 beta; Interleukin-1 receptor antagonist (IL-1RA); CD: cluster of differentiation 4, 45, 68, and 8A; FN1: Fibronectin 1; STAT3: Signal transducer and activator of transcription 3; PTPRC=CD45: Protein tyrosine phosphatase receptor type C; APOE: Apolipoprotein E; CD44: CD11b = ITGAM: Integrin subunit alpha M; CD8A: CD8 subunit alpha; CDH1: Cadherin 1: ACE: Angiotensin-converting enzyme; AGTR1: Angiotensin-2 type receptor 1; AGTR2: Angiotensin-2 type receptor 2; LL-37: LLGDFFRKSKEKIGKEFKRIVQRIKDFLRNLVPRTES; P39: DCAIVYAYD.

**FIGURE 2:**
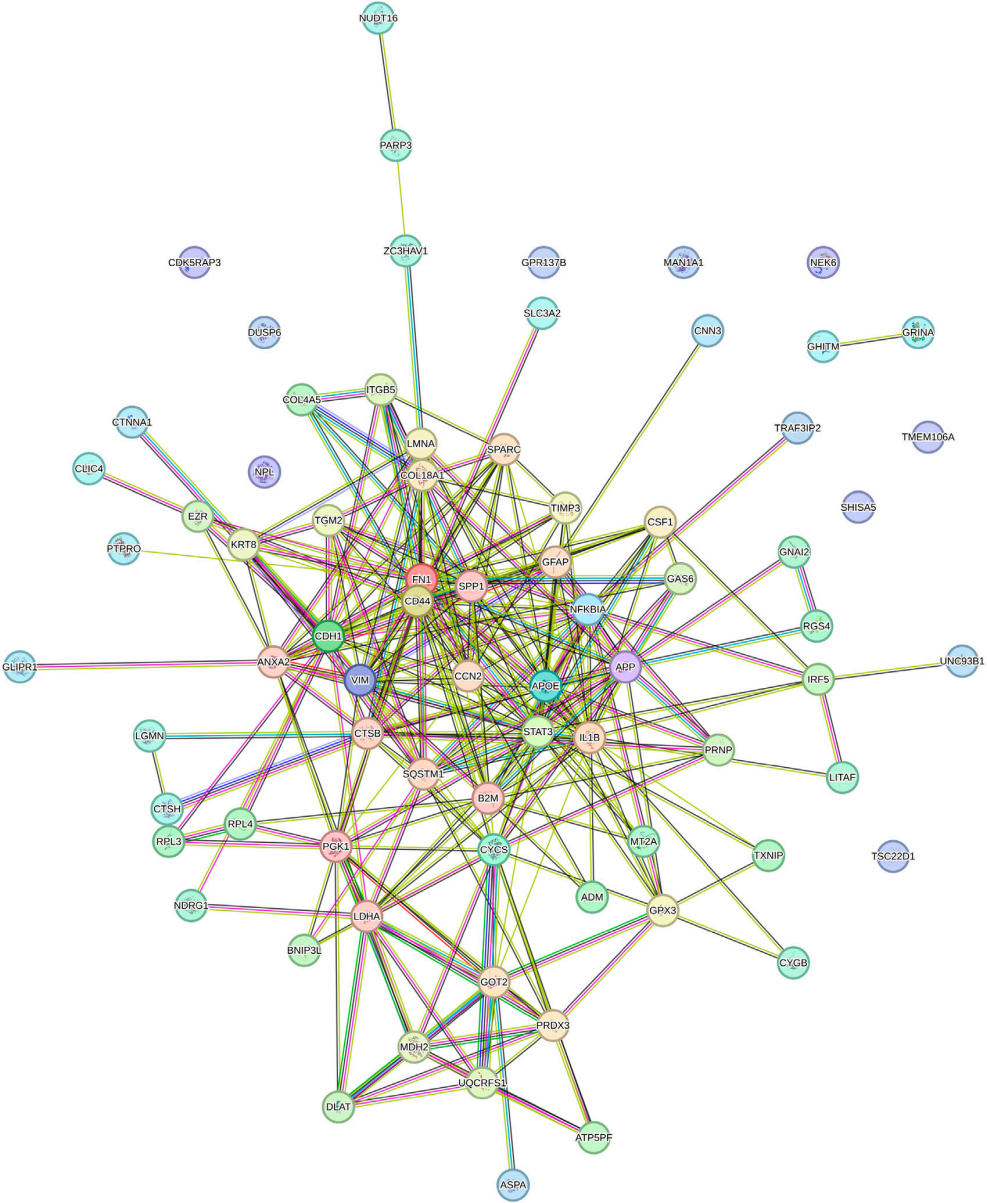
The protein-protein interaction (PPI) network of aging genes of the kidney enriched by druggable ACE, AGTR1, and AGTR2 proteins. PPI network was constructed and analyzed using STRING database, accessible at https://string-db.org/cgi/network. Spheres represent the nodes, while lines or edges indicate the interactions between them.

**FIGURE 3:**
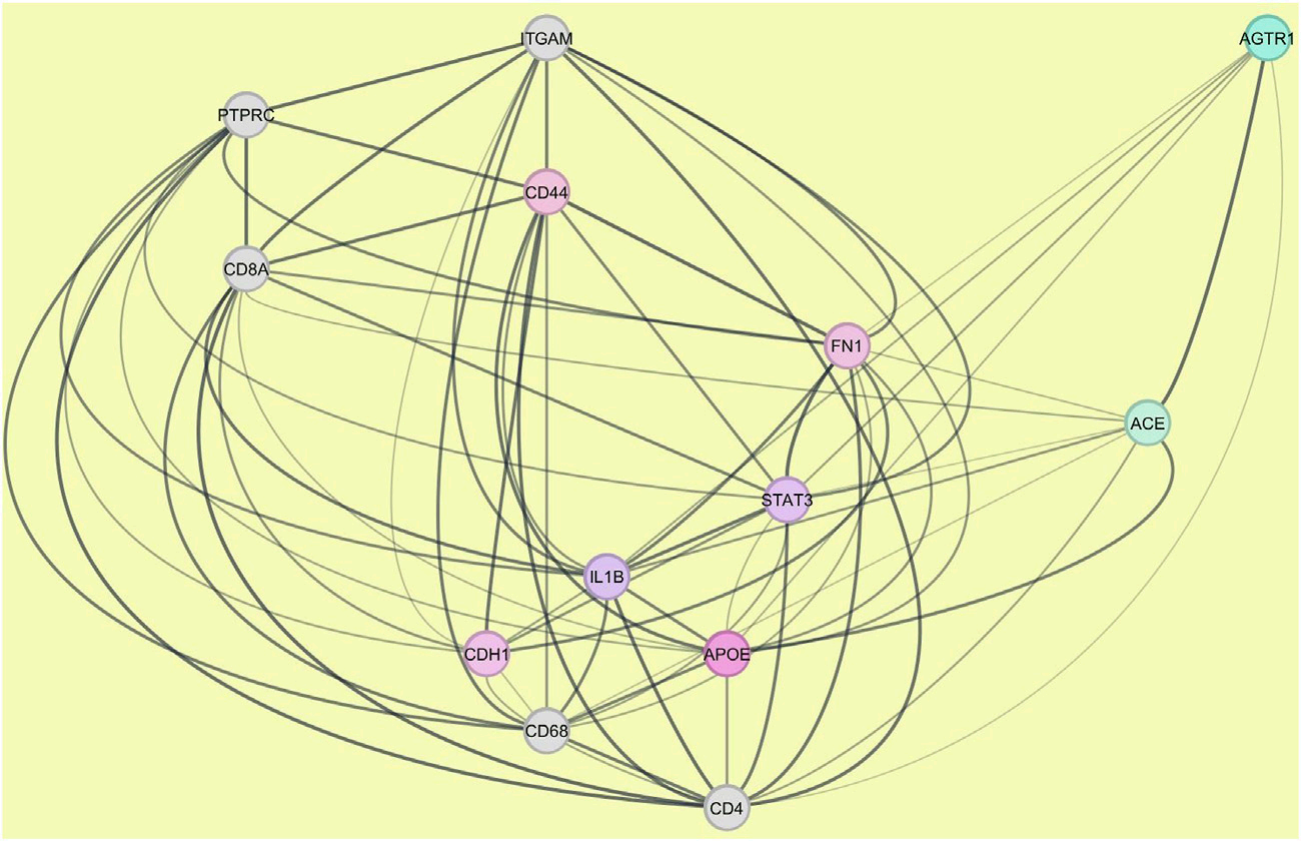
The protein-protein interaction (PPI) network constructed using the top-ten ranked nodes of the aging PPI network and the top-five nodes of the kidney-associated aging PPI subnetwork. PPI network was constructed and analyzed using STRING database, accessible at https://string-db.org/cgi/network. Spheres represent the nodes, while lines or edges indicate the interactions between them.

In this context, the subset of nodes that was ontologically associated with renal expression was culled, three major druggable targets of antihypertension, including ACE, AGTR1, and AGTR2, were added, and a new PPI network associated with the kidney was constructed (Figure 2). The resulting network was analyzed using the above-mentioned plugins, and the top-five nodes were reported (Table 1 of Supplementary File S1; Table 1). In conclusion, the trio of ACE, AGTR1, and AGTR2 interacted with other proteins that are associated with renal aging and subsequently organismal or general aging. The summary statistics of kidney-associated PPI network analyzed by StringApp were as follows: number of nodes: 78; number of edges: 310; average number of neighbors: 9.224; network diameter: 6; network radius: 3; characteristic path length: 2.437; clustering coefficient: 0.523; network density: 0.140; network heterogeneity: 0.852; network centralization: 0.340; connected components: 11; analysis time (sec): 0.125; and PPI enrichment p-value: <1.0e^−16^. The analysis of the kidney-associated PPI network using cytoHubba is shown in the Table 1 of Supplementary File S1, which contains various local- and general-based methods of network analysis. Accordingly, the top-five essential nodes (FN1, IL1B, STAT3, CD44, and CDH1) plus our targets of interest, including ACE, AGTR1, and AGTR2, were selected for their interactions with putative algal peptides of *Pariset* (Figure 3).

**FIGURE 4:**
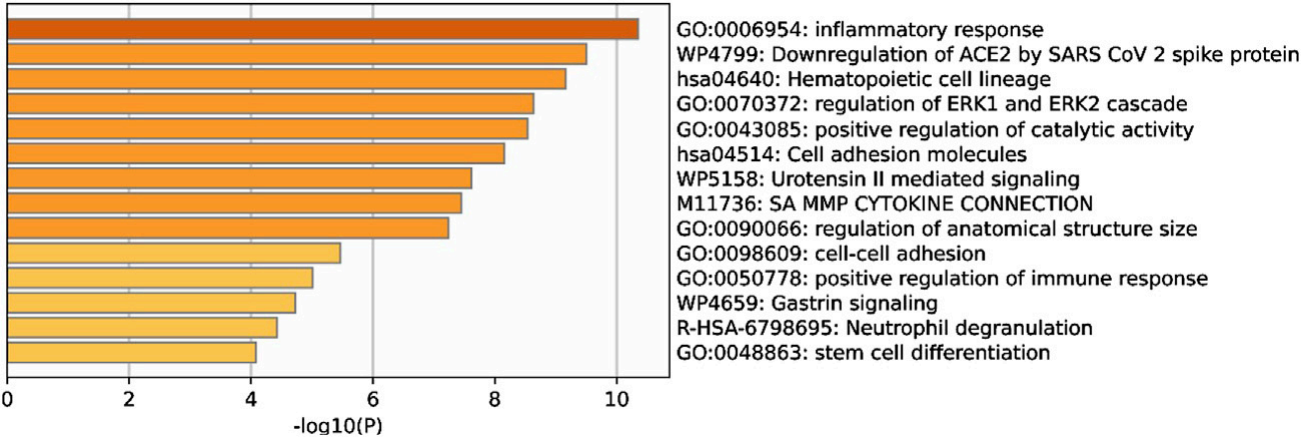
Metascape database performed enrichment analysis of hub genes of the kidney-associated network. Bar chart displaying clustered enrichment of ontology categories, including gene ontology (GO) and Kyoto Encyclopedia of Genes and Genomes (KEGG) terms. Heatmap showing the top 14 clusters colored according to the p-value (-log 10(p)) darker color indicates a lower p-value.

Enrichment analyses depicted their enrichment of hub genes involving kidney aging, mainly gene ontologies including inflammatory responses, regulation of ERK1 and ERK2 cascade, positive regulation of catalytic activity, and cell-cell adhesion, and other processes (Figure 4).

### 3.2 The chemical-protein interaction networks

**FIGURE 5:**
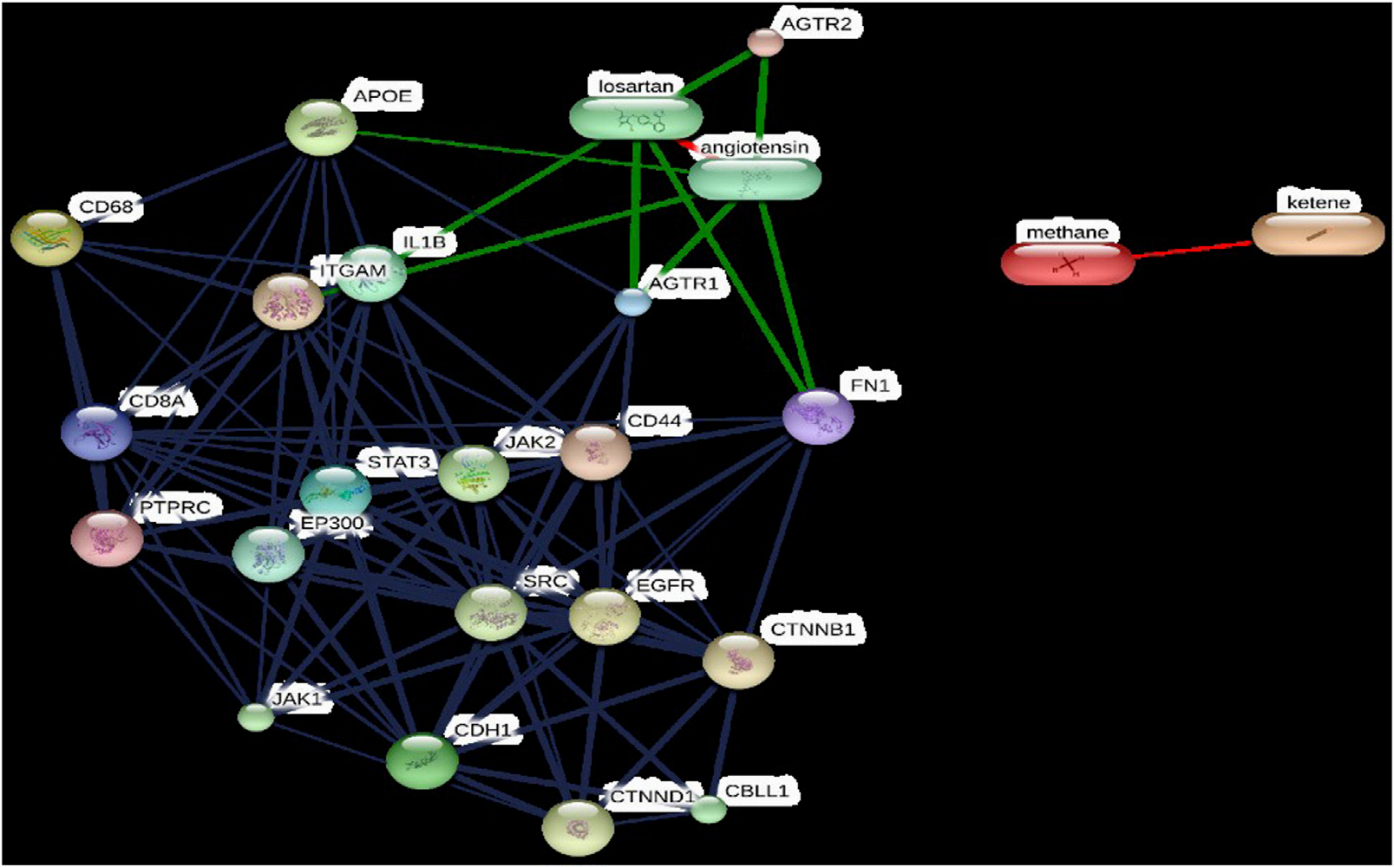
The confidence view of the target-chemical interaction network constructed using the top-ten ranked nodes of the ageing protein-protein interaction (PPI) network and the top-five nodes of the kidney-associated ageing PPI subnetwork submitted to STITCH, accessible at http://stitch.embl.de. Note: Thicker lines represent stronger associations. PPIs are shown in grey, chemical-protein interactions are shown in green, and interactions between chemicals are shown in red. See text for details of nodes.

The STITCH results showed that all hub genes that constructed the chemical-target network are highly interacted with losartan and angiotensin (Figure 5). Losartan interacts with IL1B, although there is no experimental data to support this interaction (Figure 5). The STITCH also enriched the interactions of the renal and organismal hub aging network by presenting new interacted nodes like JAK1, JAK2, EP300, SRC, EGFR, CTNNB1, and CBLL1 (Figures 3, 5). In this study, EP300 interacted with an array of top-selected nodes, including STAT3, CD44, CDH1, ITGAM, and IL1B in the kidney-associated aging PPI network (Figure 5).

### 3.3 Allergenicity, antigenicity, toxicity, and anti-aging potential of algal antihypertensive peptide

Fifty-four types of antihypertensive peptides were selected from the *Pariset*. After assessing allergenicity, antigenicity, and toxicity, seven non-toxic and non-allergenic peptides were selected. Six peptides showed sequence similarity with anti-aging peptides and were selected for further steps (Supplementary File S2), while one peptide, AAGGSLFEEYMR, was excluded because it was not confirmed as anti-aging peptide when submitted to the AagingBase database (*vide supra*).

### 3.4 Molecular docking validation of anti-aging algal peptides and aging targets

KTFPY interacted with all hub proteins in the final PPI network of kidney aging, exhibiting reliable BAs compared to those of other proteins (Supplementary Table 2). This algal peptide also interacted with the ACE-AT1R-AT2R module, showing lower but acceptable binding affinities (Supplementary Table 2). In this continuum, KTFPY exhibited BA with CD44 stronger than that of KPSSPPEE, a known CD44 peptide inhibitor. In this regard, KTFPY formed wide array of hydrogen bonds with CD44 than those formed by KPSSPPEE (Supplementary File S3).

All algal peptides interacted with CDH1 in the loosest mode among other target proteins (Table 2). In this line, PGDTY employed both hydrophobic interactions and hydrogen binds for docking with CDH1 (Supplementary File S3). VYRT exhibited maximum BA with CDH1 using hydrophobic interactions (Supplementary File S3). This peptide interacted with ACE using hydrophobic interactions with maximum BA among other algal peptides and angiotensin (Table 2; Supplementary File S3). VYRT also interacted with FN1 strongly than its known antagonist, pUR4 (Table 2). VDHY showed the lowest BAs among algal peptides with protein targets except the ACE-AT1R-AT2R module (Table 2; Supplementary File S3). It interacted with ACE using a bunch of hydrogen bonds (Supplementary File S3). PVAFN interacted with FN1 better than its inhibitor, pUR4, while showing the lowest BA with the ACE-AT1R-AT2R module among algal peptides (Supplementary Table 2). PGDTY interacted hydrophobically with STAT3 with maximum BA compared to other algal peptides (Table 2). After KTFPY, PGDTY showed the highest BA with CDH1 using hydrophobic interactions (Supplementary File S3).

**Table 2:** Molecular docking of selected algal antihypertensive peptides with aging targets.

Algal peptide	Binding affinity (-∆G) Kcal/mol with aging target proteins												
	STAT3	FN1	IL1R	CD4	CD68	CD8A	CD44	CDH1	ITGAM	CD45	ACE	AT1R	AT2R
KTFPY	352.1	350.6	369.3	313.5	329.0	299.2	**357.8**	165.4	317.7	309.3	322.4	297.6	281.1
MTFF	318.5	298.9	314.0	273.5	301.9	273.4	310.5	155.5	286.7	263.1	356.9	301.4	264.5
PGDTY	335.1	311.7	317.5	301.6	290.4	273.4	322.4	164.7	316.1	260.4	318.0	294.1	251.1
PVAFN	297.9	304.0	294.3	290.4	334.3	282.1	309.4	135.9	281.3	270.6	304.7	277.1	254.7
VDHY	284.3	296.5	310.8	265.9	289.1	268.5	266.3	107.2	272.6	261.5	325.2	306.6	264.9
VYRT	337.4	326.9	341.8	301.1	307.8	282.3	316.4	175.5	279.6	304.6	358.5	335.5	279.2
DRVYIHPF											340.2	348.9	310.4
DRVYIHPFHL											373.1		
IL1B			713.7										
pUR4		302.3											
KPSSPPEE							333.4						

Note: PDB/protein name: 4ZIA/STAT3, 3M7P/FN1, 1G0Y/IL1receptor, 1CDJ/CD4, P34810/CD68, 1CD8/CD8A, 1UUH/CD44, 2O72/CDH1, 1NA5/ITGAM, 1YGU/CD45, 1O8A/ACE, 7F6G/AT1R, 7JNI/AT2R, 1T4Q/ IL1B; pUR4 sequence is GSKDQSPLAGESGETEYITEVYGNQQNPVDIDKKLPNETGFSGNMVETEDTKLN; IL-1R: interleukin 1 receptor.

MTFF interacted with ACE using hydrogen bonds and hydrophobic interactions with maximum BA among protein targets (Table 2). It also interacted with STAT3 using hydrogen bonds and hydrophobic interactions and with reliable BA (Table 2). In conclusion, antihypertensive and putative antiaging algal peptides showed high BAs with ACE as their main targets, however, they showed higher BAs with other target proteins that are putatively involved in kidney aging.

The ACEIs showed reliable and stronger BAs with their canonical target, ACE; however, some ACEIs, like Quinapril and Benazepril, showed stronger BAs with other protein targets (Table 1 of Supplementary File S4). Moexipril Hydrochloride showed the strongest BA with ACE among other ACEIs. The BAs of all ACEIs except Trandolapril were stronger for AT1R compared to those for AT2R (Table 1 of Supplementary File S4). CD44, CD45, CD68, and FN1were protein targets for some ACEIs with reliable BAs. For example, Quinapril and Benazepril interacted with these targets in a trustworthy manner.

## 4 Discussion

Kidneys play a critical role in maintaining fluid balance, waste elimination, blood pressure regulation, and drug metabolism; therefore, preserving renal health is essential to counteract systemic aging (Papacocea et al., 2021; Dybiec et al., 2022). Hypertension, which can both contribute to and result from kidney disorders, emerges as a pivotal factor in renal aging (Boima et al., 2025). Managing blood pressure is thus crucial for mitigating the progression of kidney damage (Semenikhina et al., 2025). This highlights the importance of pharmacological interventions aimed at preventing or treating hypertension, as they not only support cardiovascular health but also enhance renal function, thereby reducing age-related deterioration. The present study aimed to investigate the potential anti-aging properties of antihypertensive peptides derived from algae. To achieve this, we constructed a PPI network based on genes associated with aging, subsequently selecting antihypertensive peptides from algae and assessing their toxicity, allergenicity, and similarity to established anti-aging peptides. Molecular docking was then employed to evaluate the interactions of these peptides with key proteins related to aging in the kidney.

PPI network analysis revealed several significant proteins that are involved in renal and organismal aging. In this context, interleukin-1 beta (IL-1β) is a proinflammatory cytokine that triggers immune responses, including the synthesis of prostaglandins, the activation of neutrophils, and the stimulation of T cells and B cells. It promotes Th17 differentiation and, in conjunction with IL-12, induces IFN-γ synthesis in Th1 cells, thereby contributing to inflammation and immune regulation. However, CD4 is a T-cell glycoprotein that aids the immune response by acting as a coreceptor for MHC class II molecules, facilitating antigen recognition and interaction with the T-cell receptor on antigen-presenting cells (APCs). This protein has a specific function in virus entry into cells, and antibodies and nanobodies have been reported for it (Zhu et al., 2024). Additionally, this protein plays key roles in the health and disease status of the kidneys (van der Putten et al., 2021). This protein is overexpressed in the processes of organismal aging and it is involved in kidney aging (Escrig-Larena and Mittelbrunn, 2025). This protein is not druggable, necessitating modulation through vaccines, particularly epitope-based ones. Although no association of this protein has been determined, it may be involved in inflammatory conditions and autoinflammatory syndromes, hastening the aging process (Di Cianni et al., 2013; Dinarello et al., 2012). Because this protein interacts physiologically with many antagonists, including anakinra, rilonacept, and canakinumab (Dinarello et al., 2012; Wijdan et al., 2025), In this study, the interactions of selected antiaging peptides and ACEIs with IL-1RI and IL1B were pursued; however, ACEIs did not express strong BAs with this IL-1R-IL1B couple. In conclusion, this protein is one of the top players of organismal aging and renal aging and more investigations are requested here.

A noteworthy protein identified was FN1 (fibronectin), which is crucial for cell adhesion and tissue repair. Dysregulation of FN1 has been associated with kidney fibrosis and age-related damage (Spada et al., 2021). Importantly, several inhibitors, including antibodies and small molecules, have been developed to mitigate its effects (Lee et al., 2019). FN1 is a key node in the kidney-associated aging network and a major component of the organismal aging PPI network. It binds to collagen, fibrin, heparin, DNA, and actin, playing essential roles in cell adhesion, motility, wound healing, and tissue maintenance. FN1 is crucial for osteoblast mineralization and collagen regulation, with dysregulated splicing linked to reduced lifespan in mice (Muro et al., 2003). FN1 inhibitors, including monoclonal antibodies and small molecules, modulate cell behavior, reduce inflammation, and may reverse fibrosis, with therapeutic applications in fibrotic diseases such as liver cirrhosis, pulmonary fibrosis, and kidney fibrosis (Spada et al., 2021). These inhibitors help prevent excessive extracellular matrix accumulation, making FN1 a promising target in anti-aging and disease management strategies. In this line, pUR4 (sequence: GSKDQSPLAGESGETEYITEVYGNQQNPVDIDKKLPNETGFSGNMVETEDTKLN) was introduced as a peptide inhibitor of FN1 (Lee et al., 2019). Moreover, FN1 showed reliable BAs with three ACEIs including quinapril, moexipril hydrochloride, and benazepril in the present study. Therefore, FN1 is involved in kidney diseases and overexpressed during aging and considered a pro-longevity gene in mice (Muro et al., 2003; Bowers et al., 2019).

Signal transducer and activator of transcription 3 (STAT3) is a key node in the kidney-associated aging and the organismal aging PPI networks. STAT3 regulates inflammatory responses and mediates cellular signaling from interleukins, growth factors, and cytokines (Rehman et al., 2025). Activated by IL31, it influences differentiation and aging. STAT3 alterations reduce lifespan, highlighting its role in longevity and age-related processes (Shi et al., 2025). Their roles in intracellular signaling pathways suggest factors such as STAT3 might be involved in aging and/or age-related disease (De-Fraja et al., 2000). Expression levels of JAK-STAT signaling targets are increased with age, and STAT3 has been implicated in the regulation of self-renewal and stem cell fate in several tissues. Knockdown or pharmacological inhibition of the JAK-STAT enhances satellite stem cell division potential resulting in better muscle regeneration (Price et al., 2014). STAT3 has been associated with age-related heart failure in mice (Jacoby et al., 2003). STAT3 has a pivotal role in the pathophysiology of kidney injury by counterbalancing resident macrophage phenotypes under inflammatory conditions (Pace et al., 2019). Pharmacological inhibition of STAT3 signaling can be achieved by direct targeting of STAT3 or upstream signaling elements. In this study, ACEIs did not present reliable BAs with STAT3, therefore STAT3 cannot be considered as druggable target.

Protein tyrosine phosphatase receptor type C (PTPRC; CD45) is a key node that is overexpressed in the organismal aging PPI networks. CD45 is essential for T-cell activation via the antigen receptor and enhances coactivation upon binding to DPP4. Its first PTPase domain possesses enzymatic activity, while the second influences substrate specificity. Upon activation, CD45 recruits and dephosphorylates SKAP1 and FYN, modulating LYN activity. It belongs to the protein-tyrosine phosphatase family, receptor class 1/6 subfamily (Al Barashdi et al., 2021). PTP inhibitor XIX (PI-19) is a potential CD45 inhibitor used to treat organ graft rejection and autoimmunity (Le et al., 2017). Perron et al. discovered 211 (2-[(4-acetylphenyl) amino]-3-chloronaphthoquinone), a selective CD45 inhibitor that effectively suppresses T-cell responses in a delayed hypersensitivity inflammatory model with minimal toxicity to the normal immune system (Perron and Saragovi, 2018). This agent may also serve as a therapeutic option to inhibit lymphoid tumor metastasis by targeting CD45 enzymatic activity for cancer treatment. Quinapril, enalapril, and benzaperil showed trustful BAs with CD45, and this striking feature of these ACEIs acknowledges further investigations.

Apolipoprotein E (APOE) is an interactive apolipoprotein in the kidney-associated aging and the organismal aging PPI networks. It mainly functions in lipoprotein-mediated lipid transport between organs via the plasma and interstitial fluids, and is involved in the pathogenesis of neurodegenerative diseases as a therapeutic target (Williams et al., 2020), however, this protein was not considered a druggable target to design inhibitors. Studies indicate that mutations in apoE might independently contribute to kidney dysfunction through increased mesangial expansion since apoE regulates growth as well as survival of mesangial cells (Chen et al., 2001).

CD44 is a highly interactive and overexpressed node in the kidney-associated aging and the organismal aging PPI networks. CD44 antigen is a cell-surface receptor that plays a role in cell-cell interactions, cell adhesion, and migration, helping them to response to changes in the tissue microenvironment. It participates thereby in a wide variety of cellular functions, including the activation, recirculation, and homing of T-lymphocytes, hematopoiesis, inflammation, and response to bacterial infection. It engages, through its ectodomain, extracellular matrix components such as hyaluronan/HA, collagen, growth factors, cytokines or proteases and serves as a platform for signal transduction. CD44 would be a druggable target to inhibit tumor growth. For example, one study aimed to evaluate the targeting-promising ability and antitumor efficiency of A6, a specific short peptide (KPSSPPEE) (Yazdian-Robati et al., 2023). CD44^+^ cell activation and complement filtration contribute to renal fibrosis in glomerulopathies, with the strongest expression in focal segmental glomerulosclerosis. CD44^+^ levels correlate with proteinuria, complement components in urine, and fibrosis severity, highlighting its role in kidney disease progression (Chebotareva et al., 2023). Moreover, quinapril and benazepril interacted with CD44^+^ with reliable BAs and can be considered for aging kidney.

ITGAM, CD8A, and CD68 were overexpressed during aging, but they were not reported in the PPI subnetwork of the kidney. In this regard, integrin alpha-M (ITGAM/ITGB2) mediates adhesive interactions in monocytes, macrophages, and granulocytes, facilitating the uptake of complement-coated particles and pathogens (Hou et al., 2024). Identical to CR-3, it binds the iC3b fragment of complement component C3 and likely recognizes the R-G-D peptide in C3b (Hou et al., 2024). It also serves as a receptor for fibrinogen, factor X, and ICAM1, recognizing P1 and P2 peptides in the fibrinogen gamma chain and regulating neutrophil migration (Hou et al., 2024). Pharmacological activation of CD11b, ITGAM, using the small molecule agonist leukadherin one enhances pro-inflammatory macrophage polarization, effectively suppressing tumor growth in murine and human cancer models (Schmid et al., 2018). ITGAM was reported as a peptide with various applications. In this regard, LL-37, a human antimicrobial peptide, exhibits broad antibacterial, anti-biofilm, and immunomodulatory properties, making it a promising antibiotic alternative (Zhang et al., 2016; Ridyard and Overhage, 2021).

CD8A-targeting peptides enhance T-cell tumor response, acting as immunoregulators. Analogue SC4 (p54-59) inhibited cytotoxic T lymphocytes activity and prolonged skin allograft survival, showing therapeutic potential (Choksi et al., 1998). CD68, a macrophage marker, facilitates liver invasion of Plasmodium sporozoite via Kupffer cells. Peptide P39 binds CD68, inhibiting sporozoite entry, offering a potential malaria treatment strategy (Cha et al., 2015). CDH1 encodes E-cadherin, which is an interactive node in the kidney-associated aging and the organismal aging PPI networks. It is a cancer predisposition gene linked to hereditary diffuse gastric cancer (HDGC), lobular breast cancer, and cleft lip/palate. Research is investigating CDH1 deficiency and its influence on cancer cell behavior, exploring its potential as a therapeutic target (Santos et al., 2024). One study examined signaling pathways—Snail, MAPK, SOCE, TGF-β, JAK/STAT, Wnt, and RAS—in regulating E-cadherin, cell migration, and proliferation, along with dietary influences on E-cadherin signaling (Ashkar and Wu, 2024). Interestingly, CD68 expressed reliable BAs with wide range of ACEIs including quinapril, moexipril hydrochloride, ramipril, trandolapril, and benazepril. This druggable target of ACEIs may open new research pipline for repurposing of ACEIs for age-related disorders.

Then, the subset of nodes that was ontologically associated with kidney expression was culled, three major druggable targets of antihypertension, including ACE, AGTR1, and AGTR2, were added, and a new PPI network associated with the kidney was constructed. Targeting vascular aging through dietary, lifestyle, and pharmacological interventions may slow CKD progression and associated cardiovascular disease. Standard treatments like renin-angiotensin-aldosterone system (RAAS) inhibitors, sodium-glucose cotransporter-2 (SGLT-2) inhibitors, and glucagon-like peptide-1 (GLP-1) receptor agonists influence these aging pathways (Fountoulakis et al., 2025). A myriad of studies highlighted the role of ACE, AGTR1, and AGTR2 in the process of aging (e.g., (Sarkar et al., 2025; Shen et al., 2025; Gotseva and Grahlyova, 2025; Díaz-Morales et al., 2025)). In conclusion, the trio of ACE, AGTR1, and AGTR2 interacted with other proteins that are associated with renal aging and subsequently organismal or general aging.

Enrichment analyses of kidney-associated aging PPI network depicted gene ontologies including inflammatory responses, regulation of ERK1 and ERK2 cascade, positive regulation of catalytic activity, and cell-cell adhesion, and other processes. In a seminal review (Wang G. M. et al., 2025) was underscored the pathophysiology of renal aging which leads to tubular atrophy, glomerulosclerosis, and declining function. In this case, senescence-associated macrophages play a key role in disease progression, exhibiting pro-damage responses while their aging further influences pathology. According to our findings, all ontogenies found for the renal aging network are consistent with the hallmarks of renal aging that are explained in the current review (Borri et al., 2025). In this context, renal endothelial cell (REC) dysfunction accelerates kidney aging by disrupting blood flow, filtration, and vascular integrity, leading to macrovascular and microvascular changes (Borri et al., 2025). Therefore, senotherapies targeting endothelial cell metabolism, with potential benefits for CKD patients and aged kidney transplants, highlight the need for innovations in REC rejuvenation.

The chemical-target network constructed using STITCH just presented losartan and angiotensin as an interactive chemicals for proteins that we suggested as main players of kidney aging. Losartan is an angiotensin-receptor blocker (ARB) that may be used alone or with other agents to treat hypertension (Wang Y.-b. et al., 2025). Losartan and its longer acting metabolite, E-3174, lower blood pressure by antagonizing RAAS; they compete with angiotensin II for binding to the type-1 angiotensin II receptor (AT1) subtype and prevents the blood pressure increasing effects of angiotensin II (Wang G. M. et al., 2025). In this study, losartan interacts with IL1B, although there is no experimental data to support this interaction.

The STITCH also enriched the interactions of the renal and organismal hub aging network by presenting new interacted nodes like JAK1, JAK2, EP300, SRC, EGFR, CTNNB1, and CBLL1. In this continuum, EP300, also known as E1A binding protein p300, is a transcriptional coactivator that plays a crucial role in various transcriptional events, including DNA repair. It functions as a histone acetyltransferase, regulating transcription through chromatin remodeling (Hasan et al., 2001). Histone acetylation serves as an epigenetic marker for transcriptional activation. Notably, EP300 activity declines in ageing mice (Li et al., 2002). Given its association with several proteins linked to ageing, such as WRN (Blander et al., 2002), EP300 may have a potential impact on human ageing. In this study, EP300 interacted with an array of top-selected nodes, including STAT3, CD44, CDH1, ITGAM, and IL1B in the kidney-associated aging PPI network (Figure 5). SRC, v-src sarcoma (Schmidt-Ruppin A-2) viral oncogene homolog (avian), is a non-receptor protein tyrosine kinase activated by various cellular receptors, including immune response receptors, integrins, receptor protein tyrosine kinases, G protein-coupled receptors, and cytokine receptors. It plays a key role in signaling pathways that regulate gene transcription, immune response, cell adhesion, cell cycle progression, apoptosis, migration, and transformation. It interacted with a wide variety of top-selected nodes in the kidney-associated aging PPI network. Epidermal growth factor receptor, EGFR, is a glycoprotein and oncogene involved in cell proliferation. It has been associated with age-related declines in stress-response (Enwere et al., 2004), but it is not known whether this is a cause or effect of aging. EGFR has also been related to age-related declines in rat hepatocytes (Hutter et al., 2000). Although one study on Korean people proposed EGFR as an aging gene, its role in human ageing remains to be determined (Park et al., 2009). CTNNB1, or beta-catenin, is crucial for cell adhesion, communication, growth, and wound healing. It interacts with FOXO in oxidative stress (Essers et al., 2005), maintains epidermal and hair follicle integrity (Huelsken et al., 2001) and is key in Wnt signaling for stem cell renewal (Reya et al., 2003). Temporal reduction of CTNNB1 during early fracture repair has been found to improve bone healing in old mice (Baht et al., 2015). Its role in ageing remains plausible but unconfirmed. To sum up, SRC, CTNNB1, and EGFR proteins have also emerged as a new node in the STITCH-derived network, and their relatedness to cellular senescence is reported in the AagingBase database.

Among the peptides derived from algae that were investigated, KTFPY exhibited the most significant potential. This peptide demonstrated a strong binding affinity for both IL1B and FN1, surpassing the known FN1 inhibitor pUR4 in binding affinity. These results underscore KTFPY as a promising candidate for further exploration as a potential therapeutic agent aimed at delaying renal aging. In this continuum, KTFPY is known through *in silico* digestion of *Pyropia pseudolinearis* plastid proteins (Kumagai et al., 2021). This peptide interacted with all hub proteins in the final PPI network of kidney aging, exhibiting reliable BAs compared to those of other proteins. This algal peptide also interacted with the ACE-AT1R-AT2R module, showing lower but acceptable BAs. Therefore, this algal peptide may possess anti-aging potential by destabilizing the interactions within the final PPI network of kidney aging. In this continuum, KTFPY exhibited BA with CD44 stronger than that of KPSSPPEE, a known CD44 peptide inhibitor (Yazdian-Robati et al., 2023). In this regard, KTFPY formed wide array of hydrogen bonds with CD44 than those formed by KPSSPPEE.

All algal peptides interacted with CDH1 in the loosest mode among other target proteins. In this line, PGDTY employed both hydrophobic interactions and hydrogen binds for docking with CDH1. VYRT exhibited maximum BA with CDH1 using hydrophobic interactions. This peptide has been prepared *in vitro* from *Pyropia pseudolinearis* protein (Kumagai et al., 2021) and interacted with ACE using hydrophobic interactions with maximum BA among other algal peptides and angiotensin. VYRT also interacted with FN1 stronger than its known antagonist, pUR4 (Lee et al., 2019). VDHY showed the lowest BAs among algal peptides with protein targets except ACE-AT1R-AT2R module. It interacted with ACE with a bunch of hydrogen bonds. This peptide has been prepared *in vitro* from *Pyropia pseudolinearis* protein (Kumagai et al., 2021).

PVAFN interacted with FN1 better than its inhibitor, pUR4, while showing the lowest BA with ACE-AT1R-AT2R module among algal peptides. PVAFN is known through *in silico* digestion of *Pyropia pseudolinearis* plastid proteins (Kumagai et al., 2021). PGDTY interacted hydrophobically with STAT3 with maximum BA compared to other algal peptides. PGDTY is known through *in silico* digestion of *Pyropia pseudolinearis* plastid proteins (Kumagai et al., 2021). After KTFPY, PGDTY showed the highest BA with CDH1 using hydrophobic interactions. MTFF interacted with ACE using hydrogen bonds and hydrophobic interactions with maximum BA among protein targets. It also interacted with STAT3 using hydrogen bonds and hydrophobic interactions and with reliable BA. MTFF is known through *in silico* digestion of *Pyropia pseudolinearis* plastid proteins (Kumagai et al., 2021). Although BA will give a significant cue for fitness of a molecule to be considered as a hit candidate for drug discovery, further characterization as LADMET (Liberation, Absorption, Distribution, Metabolism, Excretion, Toxicity) and molecular dynamics-based validations are acknowledged. In conclusion, antihypertensive and putative antiaging algal peptides showed high BAs with ACE as their main targets, however, they showed higher BAs with other target proteins that are computationally involved in kidney aging. We acknowledging this limitation of our computational effort and emphasizing the need for experimental validation. Specifically, we note that follow-up studies using cell-based assays, animal models, or *in vitro* ADMET profiling will be essential to confirm the functional relevance and therapeutic potential of the identified peptides. In this regard, to validate the biological activity and therapeutic potential of the identified peptides, several *in vitro* testing strategies can be employed. These include cytotoxicity and viability assays to ensure cellular safety (Mosmann, 1983), and target-specific functional assays such as enzyme inhibition, reporter gene assays, or Western blotting to confirm modulation of relevant pathways (Inglese et al., 2007). For aging-related kidney pathology, peptides can be evaluated for anti-inflammatory or anti-fibrotic effects by measuring cytokine levels (e.g., IL-6, TNF-α) or fibrosis markers (e.g., TGF-β1, collagen I) in relevant cell lines (Lee and Ha, 2005). Additional assays for oxidative stress (e.g., reactive oxygen species detection) and senescence (e.g., β-galactosidase staining) can provide further insights into potential anti-aging effects (Cadar et al., 2024; Debacq-Chainiaux et al., 2009). Moreover, *in vitro* ADMET profiling, including Caco-2 permeability, serum stability, and hepatocyte toxicity, offers early prediction of pharmacokinetic and safety properties, supporting the rationale for subsequent *in vivo* studies (Hubatsch et al., 2007; Di and Kerns, 2016).

## 5 Conclusion

In conclusion, the constructed senescence-associated PPI network highlights key aging-related proteins involved in inflammation, immunity, fibrosis, and kidney aging. Losartan, as an effective ARB, interacts strongly with these hub genes, offering therapeutic potential for hypertension and kidney-related conditions. The screening of antihypertensive peptides identified several candidates with promising anti-aging properties, particularly KTFPY, VYRT, PGDTY, PVAFN, and MTFF, which demonstrated strong binding to ACE and other aging-related targets. Classical ACEIs also interacted with aging target proteins that open new aperture for repurposing ACEIs for aging-related disorders. These findings suggest that algal-derived peptides could serve as potential therapeutics for both hypertension and aging-associated kidney dysfunction, warranting further exploration in biomedical applications.

## Data Availability

The datasets presented in this study can be found in online repositories. The names of the repository/repositories and accession number(s) can be found in the article/Supplementary Material.
