# Supplementary material for: The anti-aging potential of antihypertensive peptides of Pariset, a dataset of algal peptides
Source: Front Aging. 2025 Jul 30;6:1618082. doi: 10.3389/fragi.2025.1618082 (PMC12343563; doi:10.3389/fragi.2025.1618082)
Supplement: Supplementary file 1 [file Table1.docx]

**The anti-aging potential of antihypertensive peptides of *Pariset*, a dataset of algal peptides**

**Isaac Karimi^1^*, Parisa Olfati^1^, Layth Jasim Mohammed^2^, Jawad Kadhim Tarrad^2^, Ahmed M. Amshawee^3^, Maryam A. Hussain^4^ and Helgi B. Schiöth*^5^**

^1^Laboratory for Computational Physiology, Department of Biology, Faculty of Science, Razi University 67149-67346, Kermanshah, Iran. p.olfati1999@gmail.com

^2^Department of Microbiology, College of Medicine, Babylon University, Hilla City, Babylon Governorate, 51002, Iraq. E-mail: [med996.layth.jasim@uobabylon.edu.iq](mailto:med996.layth.jasim@uobabylon.edu.iq)

^3^Department of Radiology, University of Hilla, Babylon, Iraq. E-mail: [ahmed_meki@hilla-unc.edu.iq](mailto:ahmed_meki@hilla-unc.edu.iq)

^4^Babylon Technical Institute, AL-Furat Al-Awsat Technical University, Babylon, Iraq. E-mail: maryam.hussein.iba3@atu.edu.iq

^5^Department of Surgical Sciences, Functional Pharmacology and Neuroscience, Uppsala University, 751 24, Uppsala, Sweden.

*Correspondence: Helgi B. Schiöth, helgi.schioth@uu.se, Tel and Fax: 0046-18-4714160; Isaac Karimi; isaac_karimi2000@yahoo.com; karimiisaac@razi.ac.ir. Tel & Fax: 0098-83-34274545.


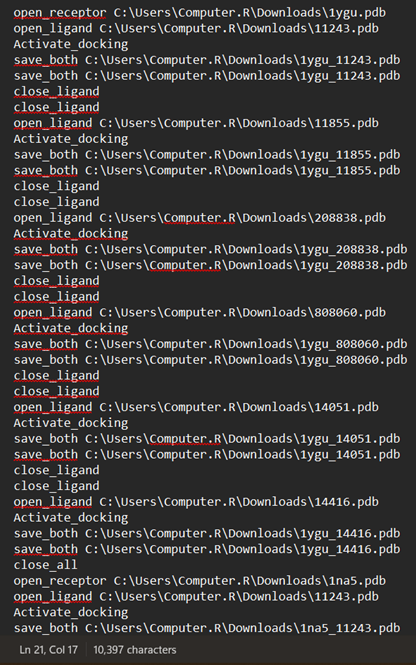


Figure 1. Serial molecular docking using Hex Macro section.

**Table 1.** The features of the top-ten ranked nodes of the ageing PPI network and the top-five nodes of the kidney-associated ageing PPI subnetwork computed by various algorithms of cytoHubba

| **Node_Name** | **MCC** | **DMNC** | **MNC** | **Degree** | **EPC** | **BottleNeck** | **EcCentricity** | **Closeness** | **Radiality** | **Betweenness** | **Stress** | **ClusteringCoefficient** |
| --- | --- | --- | --- | --- | --- | --- | --- | --- | --- | --- | --- | --- |
| 9606.ENSP00000263341 | 731520 | 0.80492 | 12 | 12 | 5.88 | 3 | 1 | 12 | 2.25 | 3.77937 | 22 | 0.83333 |
| 9606.ENSP00000346839 | 731520 | 0.80492 | 12 | 12 | 6.024 | 1 | 1 | 12 | 2.25 | 3.77937 | 22 | 0.83333 |
| 9606.ENSP00000264657 | 731520 | 0.80492 | 12 | 12 | 5.975 | 1 | 1 | 12 | 2.25 | 3.77937 | 22 | 0.83333 |
| 9606.ENSP00000011653 | 731520 | 0.80492 | 12 | 12 | 5.907 | 1 | 1 | 12 | 2.25 | 3.77937 | 22 | 0.83333 |
| 9606.ENSP00000386559 | 730800 | 0.8484 | 11 | 11 | 5.825 | 1 | 0.5 | 11.5 | 2.16667 | 1.4127 | 10 | 0.90909 |
| 9606.ENSP00000250092 | 730800 | 0.8484 | 11 | 11 | 5.888 | 1 | 0.5 | 11.5 | 2.16667 | 1.4127 | 10 | 0.90909 |
| 9606.ENSP00000252486 | 368640 | 0.7975 | 11 | 11 | 5.767 | 1 | 0.5 | 11.5 | 2.16667 | 2.72381 | 16 | 0.85455 |
| 9606.ENSP00000411355 | 725760 | 0.87792 | 10 | 10 | 5.759 | 1 | 0.5 | 11 | 2.08333 | 0.22222 | 2 | 0.97778 |
| 9606.ENSP00000398632 | 725760 | 0.87792 | 10 | 10 | 5.799 | 1 | 0.5 | 11 | 2.08333 | 0.22222 | 2 | 0.97778 |
| 9606.ENSP00000496959 | 725760 | 0.87792 | 10 | 10 | 5.515 | 1 | 0.5 | 11 | 2.08333 | 0.22222 | 2 | 0.97778 |
| 9606.ENSP00000261769 | 362880 | 0.85919 | 9 | 9 | 5.506 | 1 | 0.5 | 10.5 | 2 | 0 | 0 | 1 |
| 9606.ENSP00000290866 | 5760 | 0.75809 | 8 | 8 | 5.053 | 1 | 0.5 | 10 | 1.91667 | 0.66667 | 4 | 0.92857 |
| 9606.ENSP00000398832 | 720 | 0.71324 | 6 | 6 | 4.424 | 1 | 0.5 | 9 | 1.75 | 0 | 0 | 1 |
