# Supplementary material for: The anti-aging potential of antihypertensive peptides of Pariset, a dataset of algal peptides
Source: Front Aging. 2025 Jul 30;6:1618082. doi: 10.3389/fragi.2025.1618082 (PMC12343563; doi:10.3389/fragi.2025.1618082)
Supplement: Supplementary file 2 [file Table2.docx]

**The anti-aging potential of antihypertensive peptides of *Pariset*, a dataset of algal peptides**

**Isaac Karimi^1^*, Parisa Olfati^1^, Layth Jasim Mohammed^2^, Jawad Kadhim Tarrad^2^, Ahmed M. Amshawee^3^, Maryam A. Hussain^4^ and Helgi B. Schiöth*^5^**

^1^Laboratory for Computational Physiology, Department of Biology, Faculty of Science, Razi University 67149-67346, Kermanshah, Iran. p.olfati1999@gmail.com

^2^Department of Microbiology, College of Medicine, Babylon University, Hilla City, Babylon Governorate, 51002, Iraq. E-mail: [med996.layth.jasim@uobabylon.edu.iq](mailto:med996.layth.jasim@uobabylon.edu.iq)

^3^Department of Radiology, University of Hilla, Babylon, Iraq. E-mail: [ahmed_meki@hilla-unc.edu.iq](mailto:ahmed_meki@hilla-unc.edu.iq)

^4^Babylon Technical Institute, AL-Furat Al-Awsat Technical University, Babylon, Iraq. E-mail: maryam.hussein.iba3@atu.edu.iq

^5^Department of Surgical Sciences, Functional Pharmacology and Neuroscience, Uppsala University, 751 24, Uppsala, Sweden.

*Correspondence: Helgi B. Schiöth, helgi.schioth@uu.se, Tel and Fax: 0046-18-4714160; Isaac Karimi; isaac_karimi2000@yahoo.com; karimiisaac@razi.ac.ir. Tel & Fax: 0098-83-34274545.


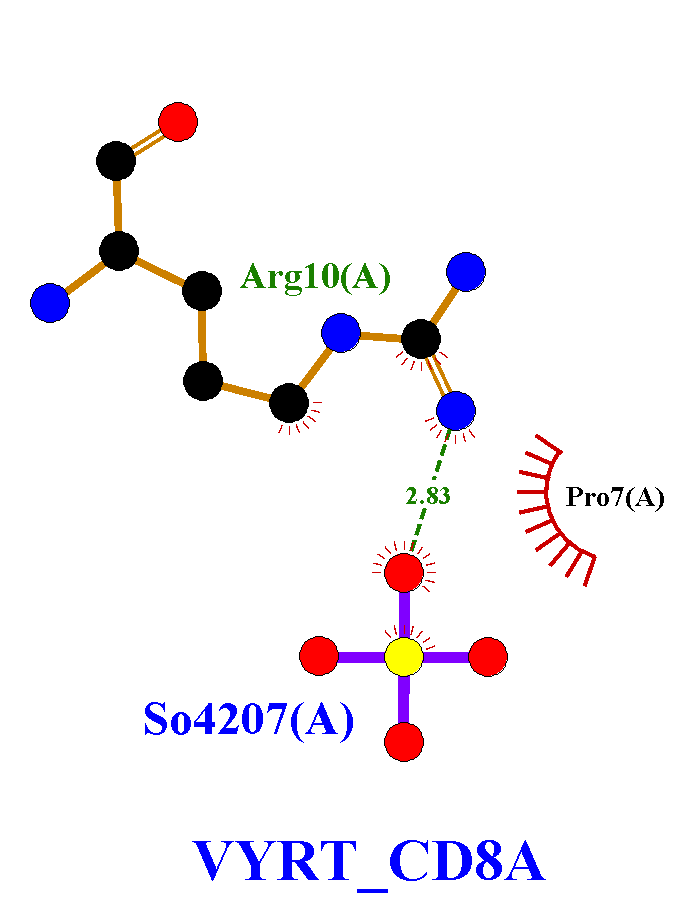

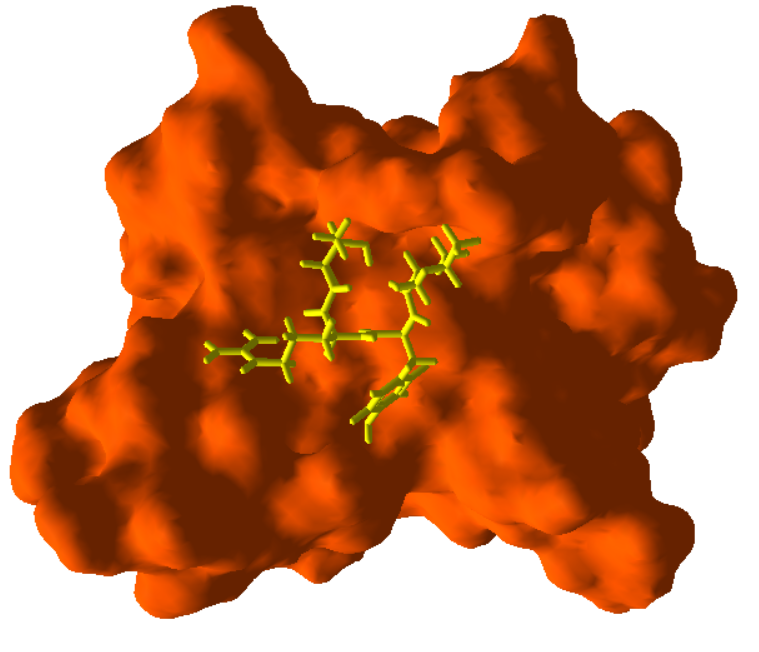


**Figure (1) VYRT_CD8A** Non-ligand bond. Corresponding atoms and non-ligand Pro7 involved in hydrophobic interactions. Hydrogen bond between VYRT and Arg10 shown by dotted *green* lines.


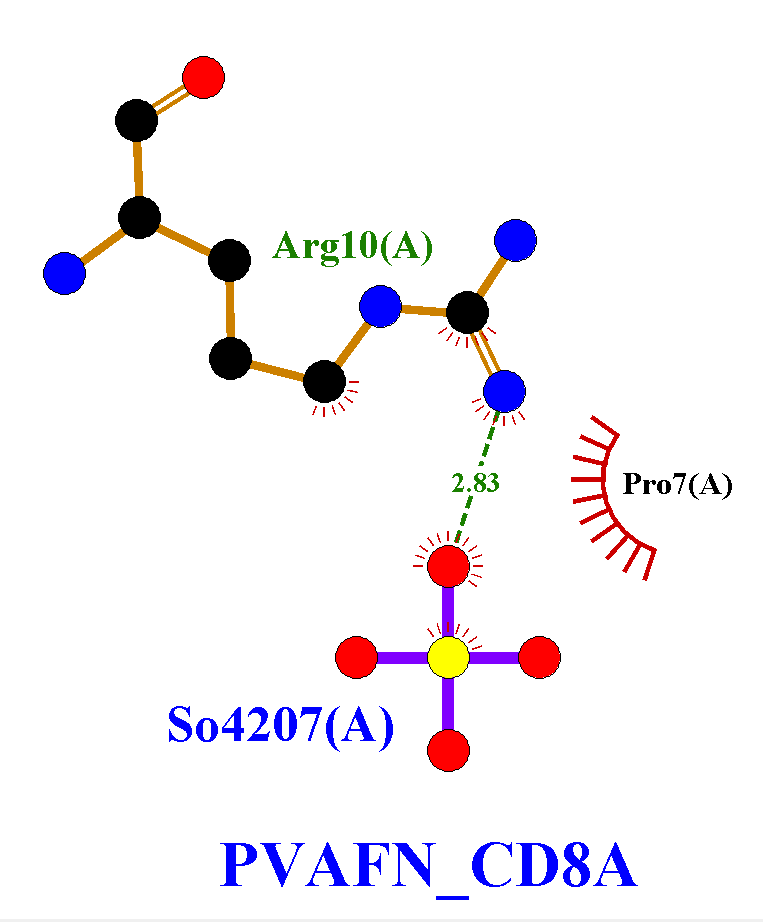

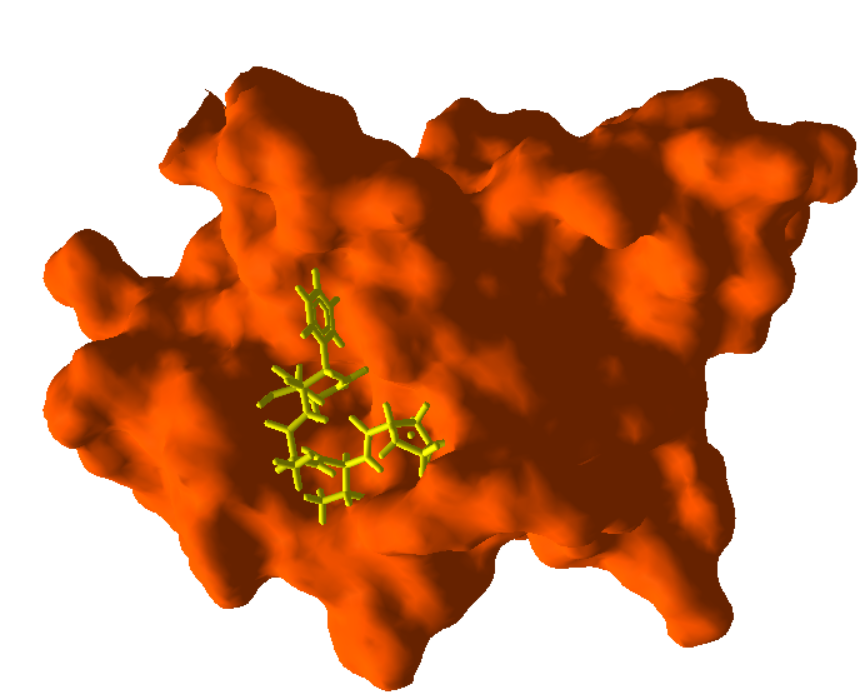


**Figure (2) PVAFN_CD8A** Non-ligand bond. Corresponding atoms and non-ligand Pro7 involved in hydrophobic interactions. Hydrogen bond between PVAFN and Arg10 shown by dotted *green* lines.


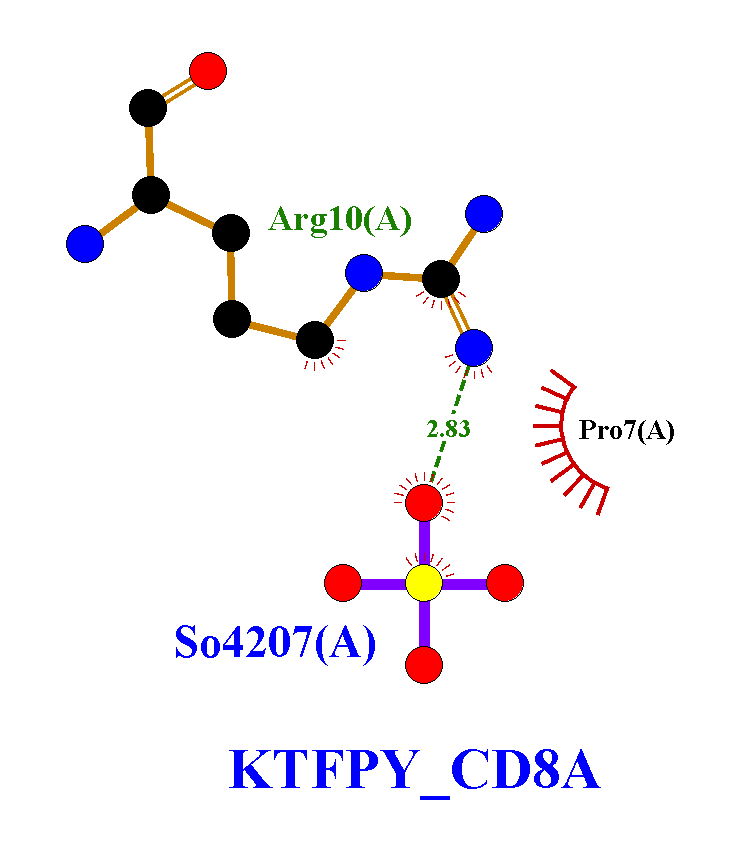

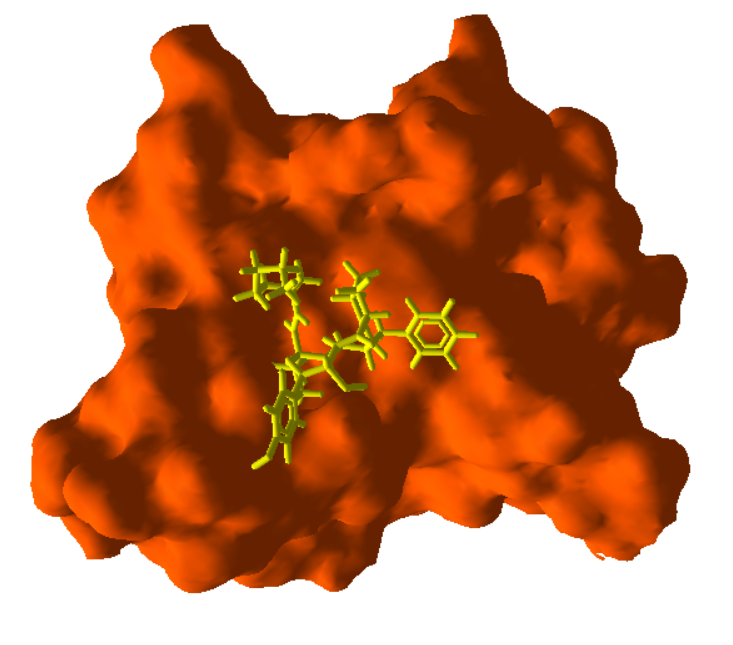


**Figure (3) KTFPY_CD8A** Non-ligand bond. Corresponding atoms and non-ligand Pro7 involved in hydrophobic interactions. Hydrogen bond between KTFPY and Arg10 shown by dotted *green* lines.


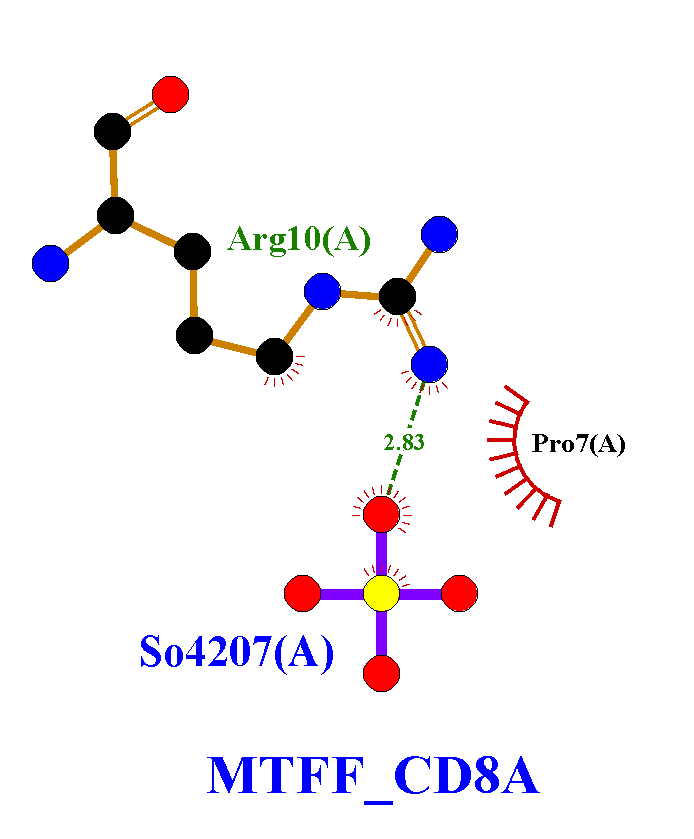

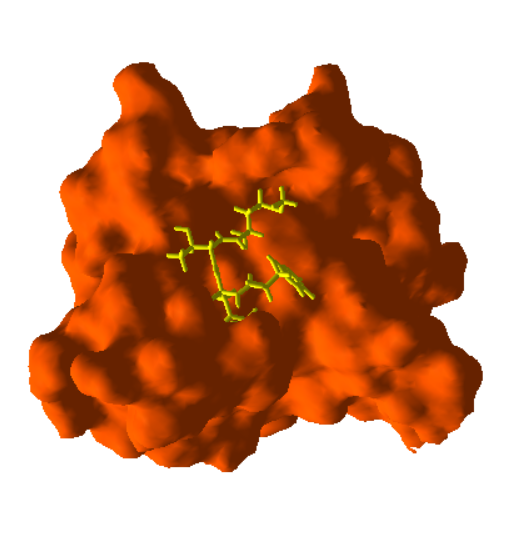


**Figure (4) MTFF_CD8A** Non-ligand bond. Corresponding atoms and non-ligand Pro7 involved in hydrophobic interactions. Hydrogen bond between MTFF and Arg10 shown by dotted green lines.


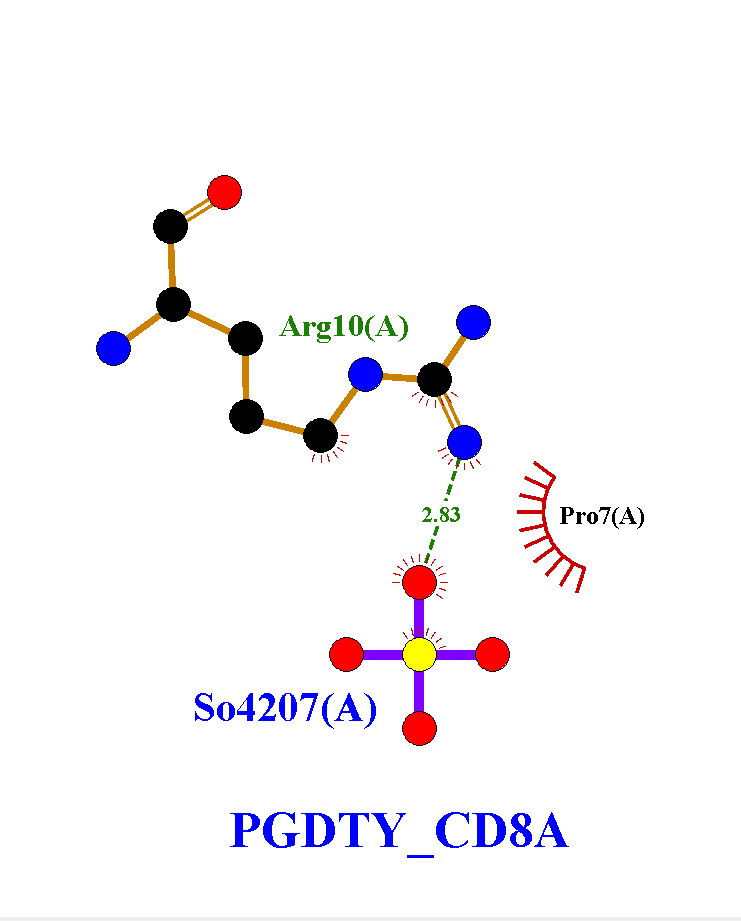

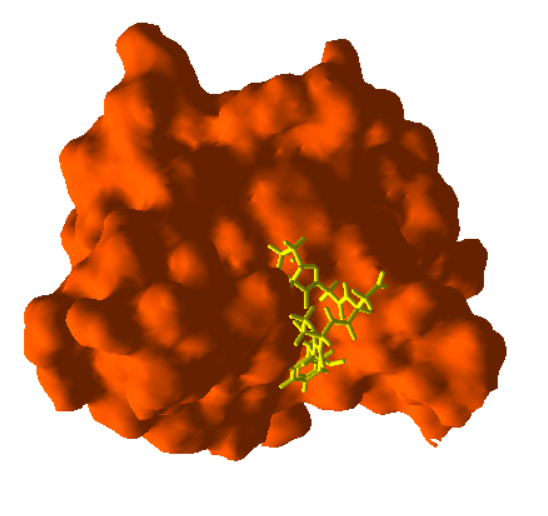


**Figure (5) PGDTY_CD8A** Non-ligand bond. Corresponding atoms and non-ligand Pro7 involved in hydrophobic interactions. Hydrogen bond between PGDTY and Arg10 shown by dotted green lines.


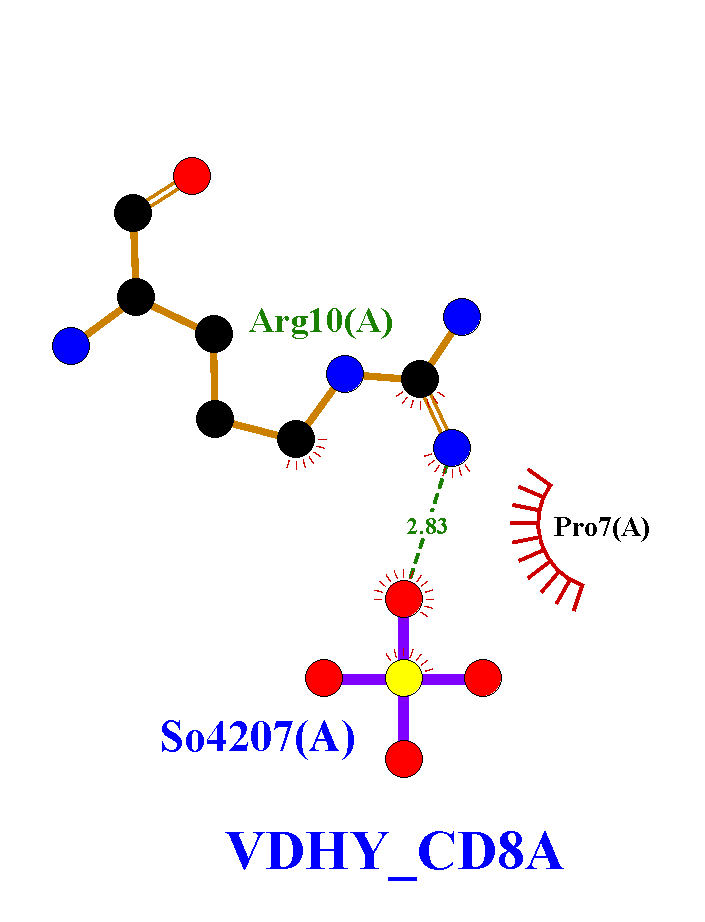

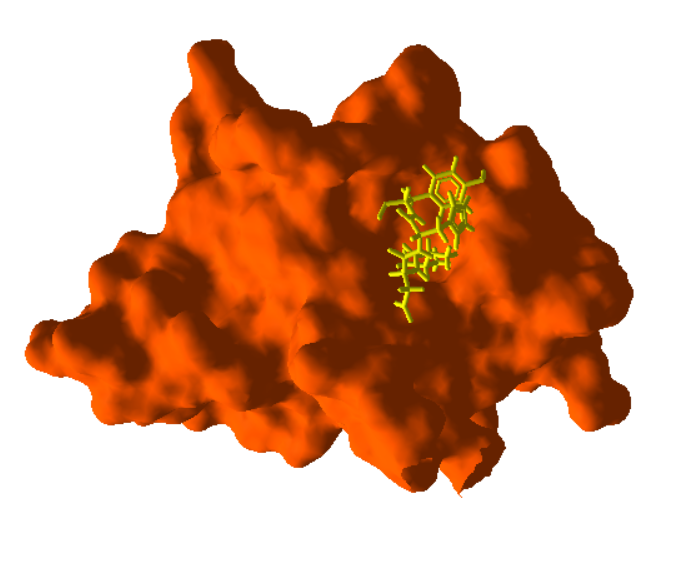


**Figure (6) VDHY_CD8A** Non-ligand bond. Corresponding atoms and non-ligand Pro7 involved in hydrophobic interactions. Hydrogen bond between VDHY and Arg10 shown by dotted green lines.


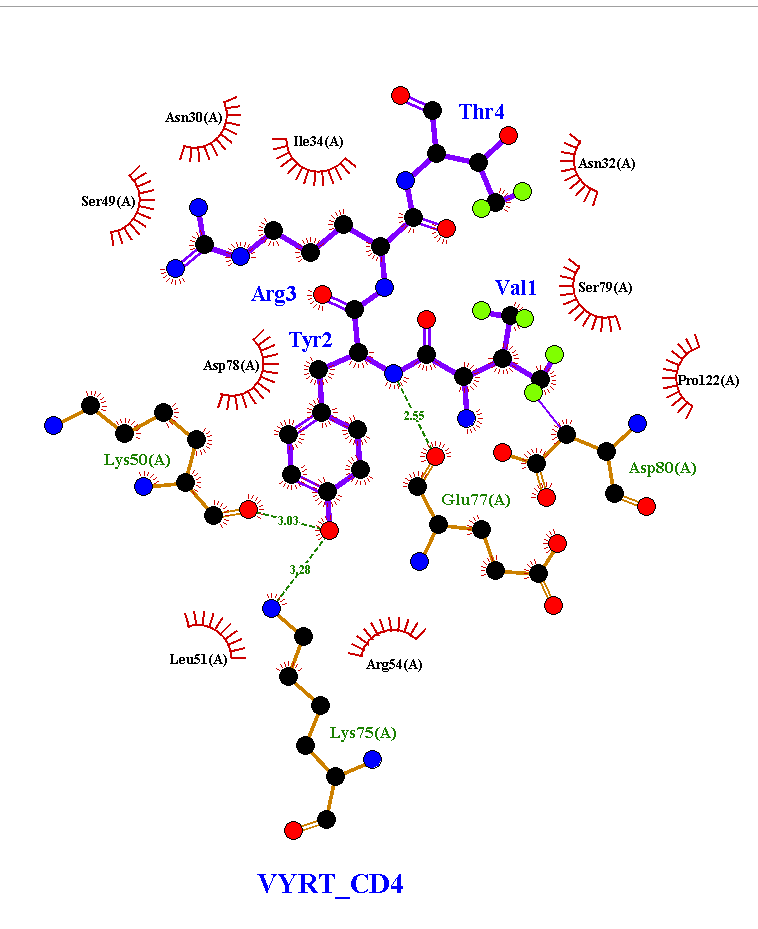

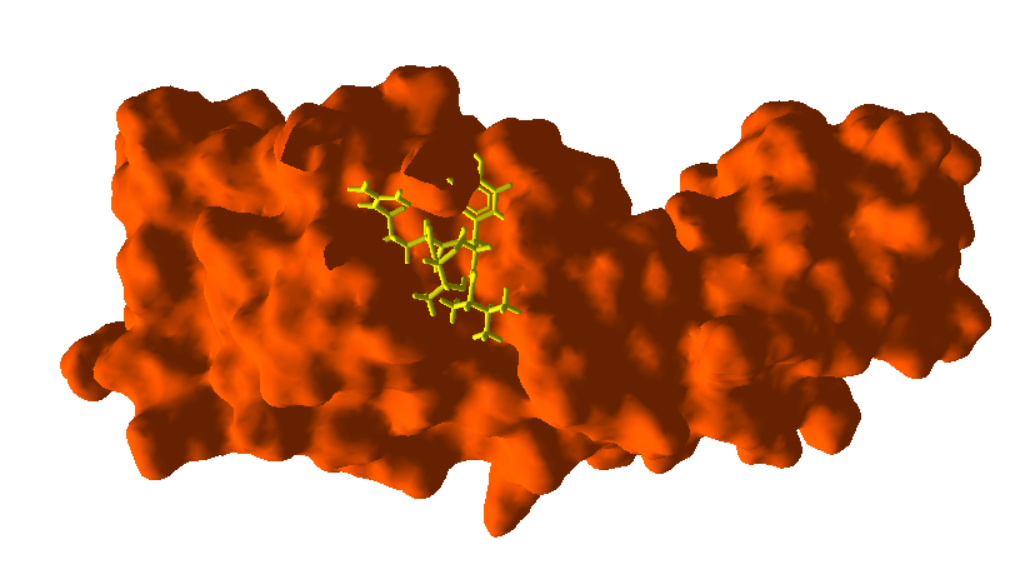


**Figure (1) VYRT_CD4** Non-ligand bond. Corresponding atoms and non-ligand Arg54, Pro122, Ser79, Asn32, Ile34, Asn30, Ser49, Asp78 and Leu51 involved in hydrophobic interactions. Hydrogen bond between VYRT and Glu77, Lys50, Lys75 and external bond with Asp80 shown by dotted green lines.


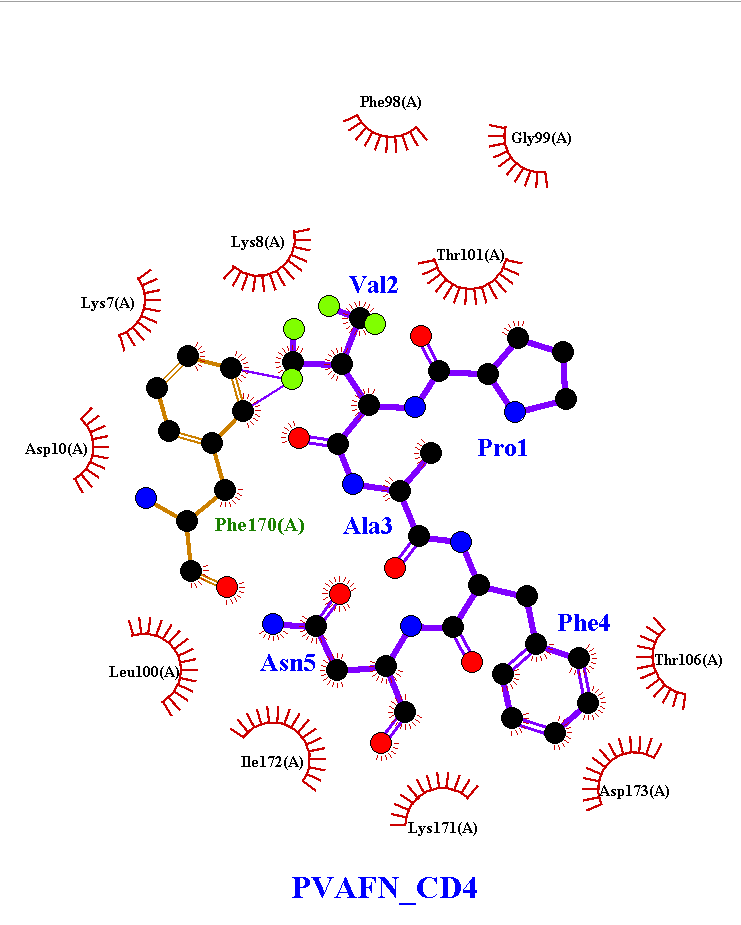

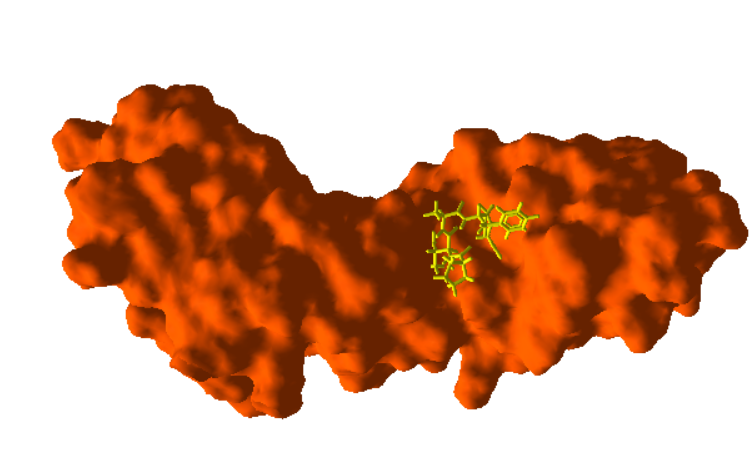


**Figure (2) PVAFN_CD4** Non-ligand bond. Corresponding atoms and non-ligand Thr106, Asp173, Lys171, Ile172, Leu100, Asp10, Lys7, Lys8, Phe98, Gly99 and Thr101 involved in hydrophobic interactions. External bonds between PVAFN and Phe170 shown by dotted purple lines.


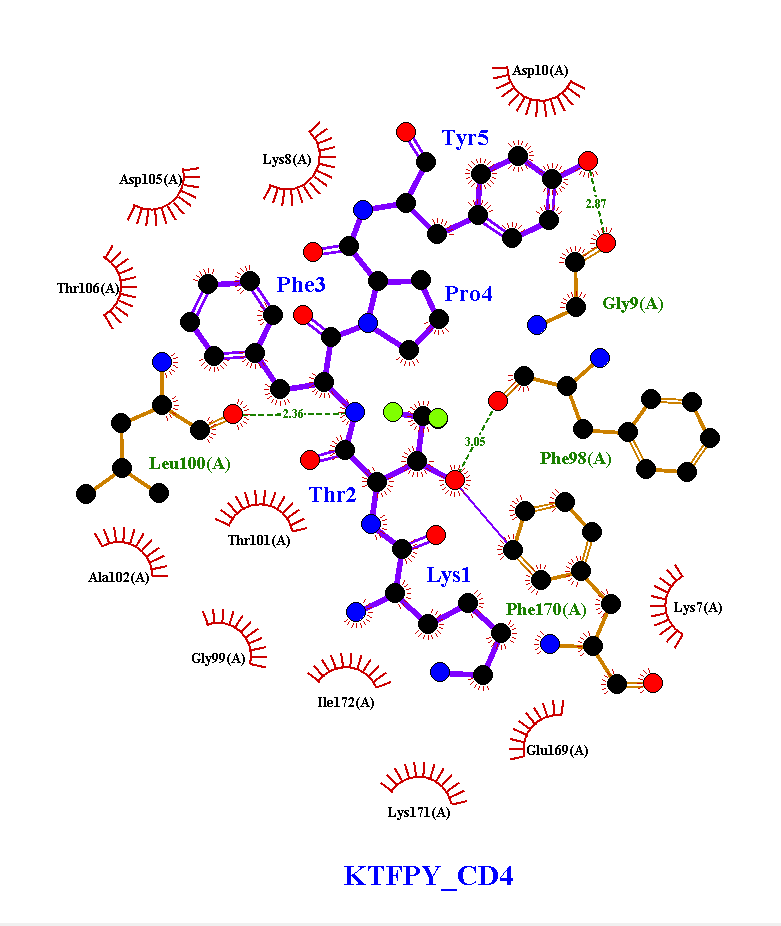

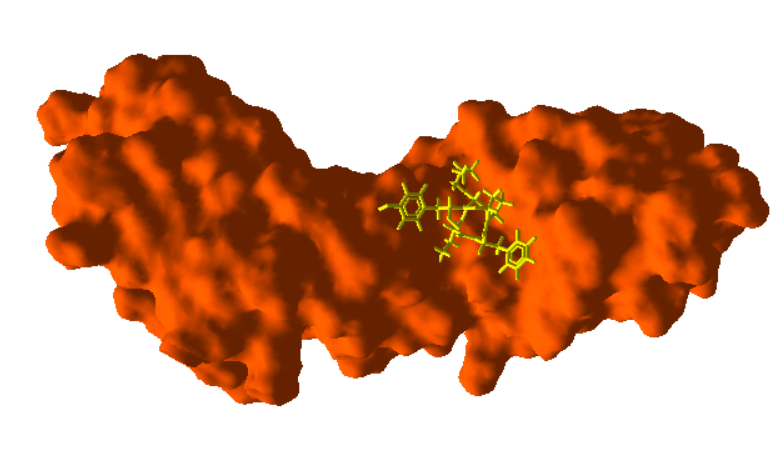


**Figure (3) KTFPY_CD4** Non-ligand bond. Corresponding atoms and non-ligand Lys7, Glu169, Lys171, Ile172, Gly99, Thr101, Ala102, The106, Asp105, Lys8 and Asp10 involved in hydrophobic interactions. Hydrogen bond between KTFPY and Leu100 and Phe98 and external bond with Phe170 shown by dotted green lines.


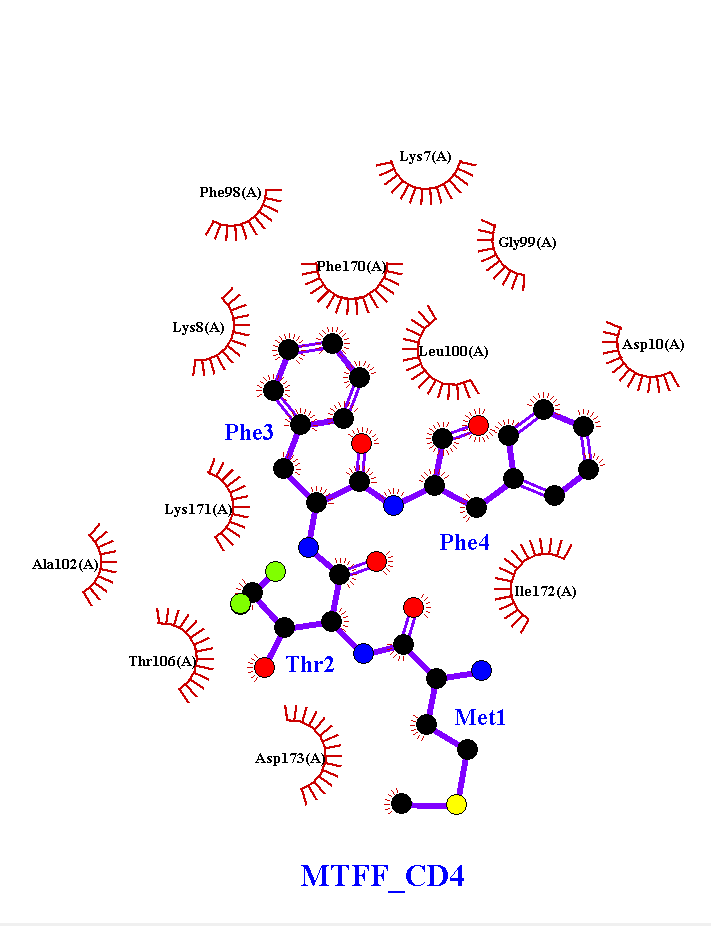

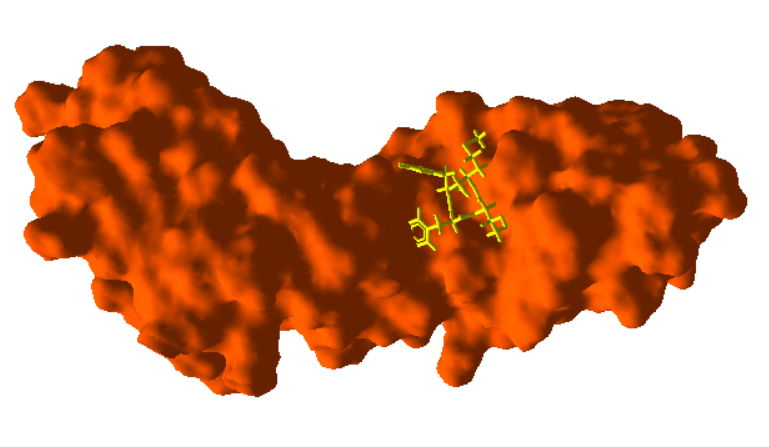


**Figure (4) MTFF_CD4** Corresponding atoms and non-ligand Ile172, Asp173, Thr106, Ala102, Lys171, Lys8, Phe98, Phe170, Leu100, Gly99, Lys7 and Asp10 involved in hydrophobic interactions.


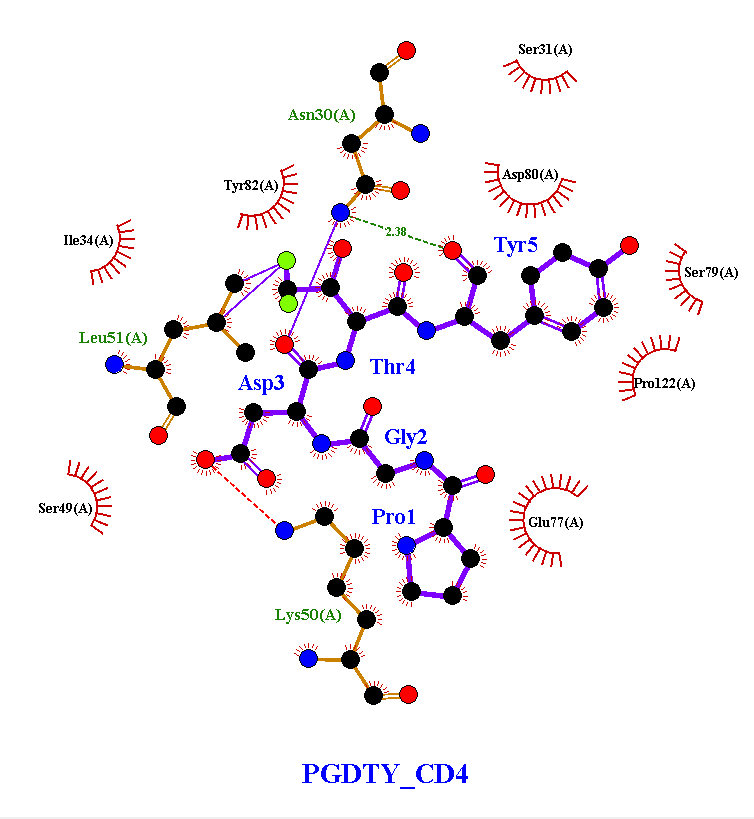

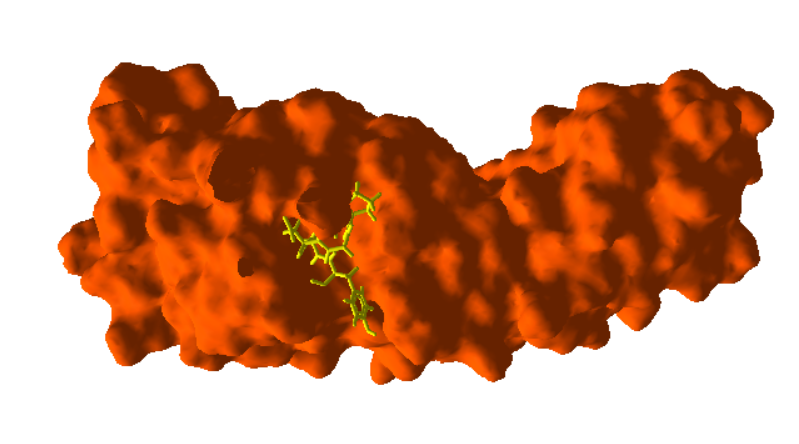


**Figure (5) PGDTY_CD4** Corresponding atoms and non-ligand Glu77, Pro122, Ser79, Asp80, Ser31, Tyr82, Ile34, Ser49 involved in hydrophobic interactions. Hydrogen bond between PGDTY and Asn30 and external bond with Leu51 and Asn50 and salt bridges with Lys50 shown by dotted green lines.


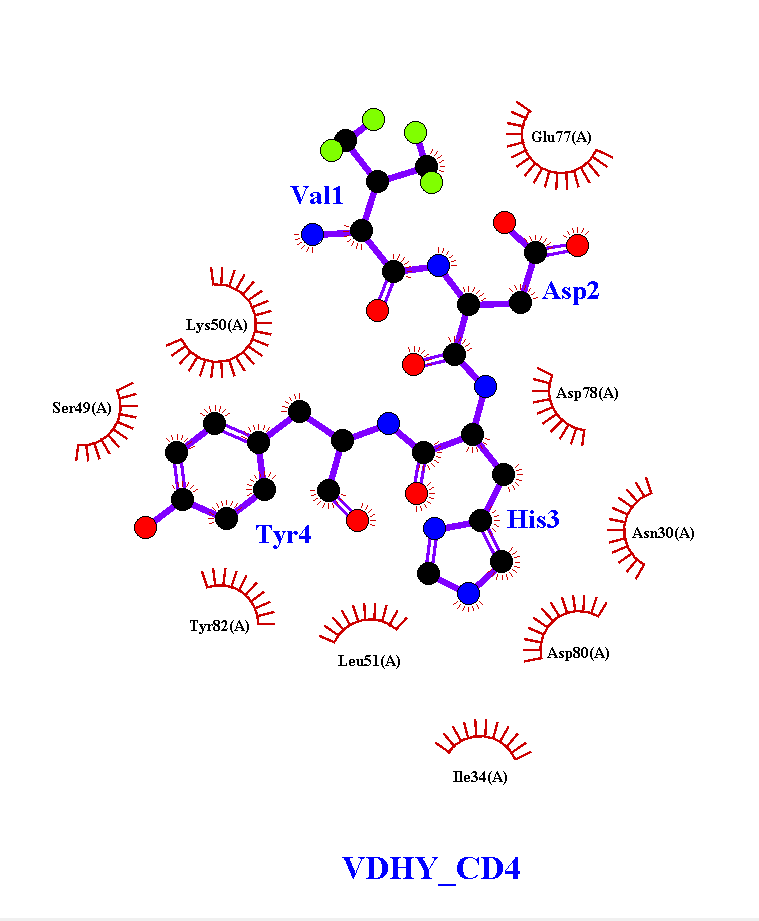

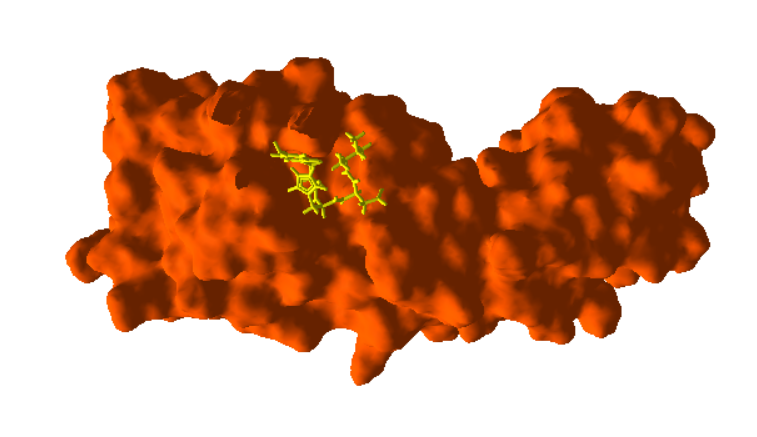


**Figure (6) VDHY_CD4** Non-ligand bond. Corresponding atoms and non-ligand Asp78, Asn30, Asp80, Ile34, Leu51, Tyr82, Ser49, Lys50 and Glu77 involved in hydrophobic interactions.


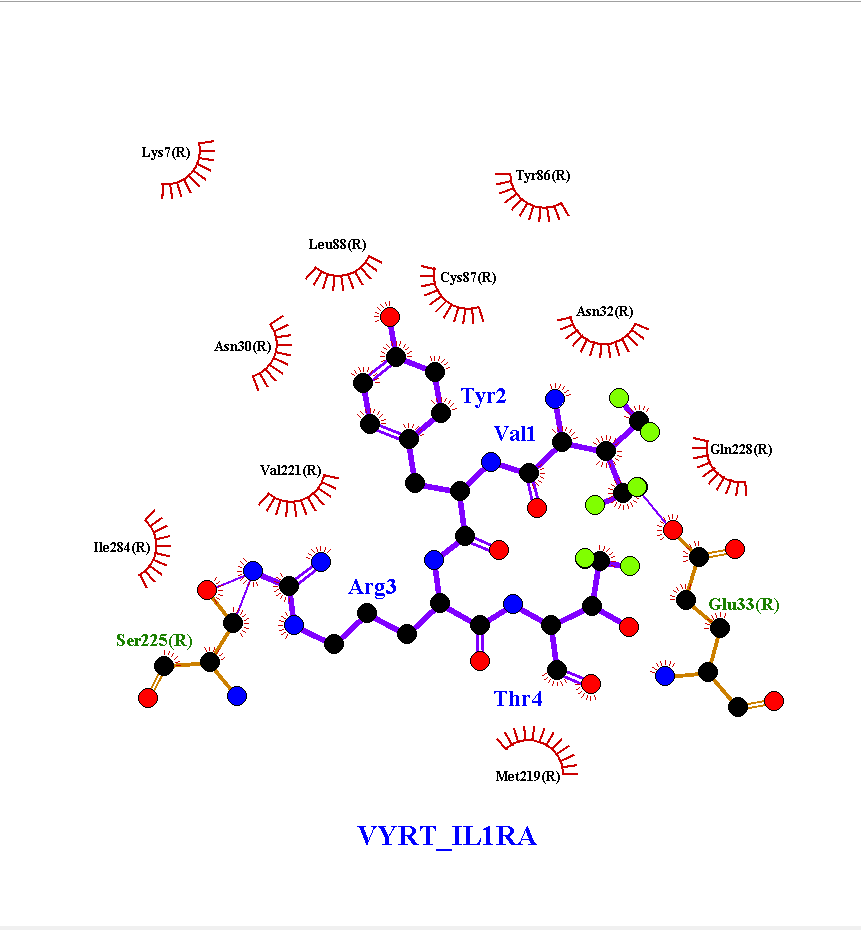

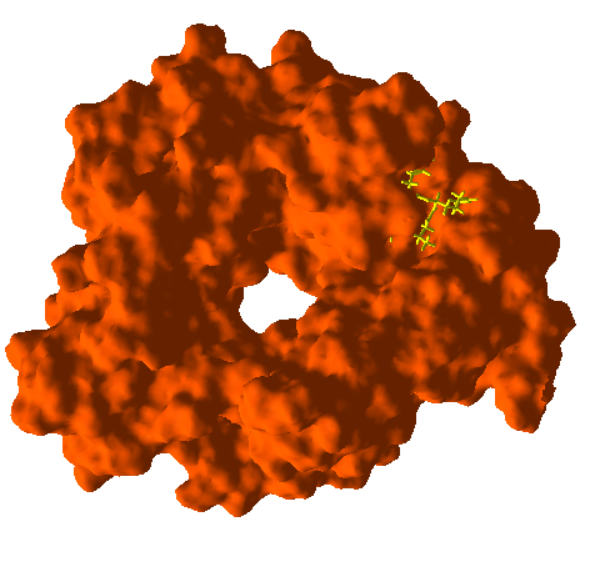


**Figure (1) VYRT_IL1RA** Non-ligand bond. Corresponding atoms and non-ligand Met219, Ile284, Val221, Asn30, Leu88, Lys7, Cys86, Tyr86, Asn32 and Gln228 involved in hydrophobic interactions. External bonds with Ser225 and shown by dotted purple lines.


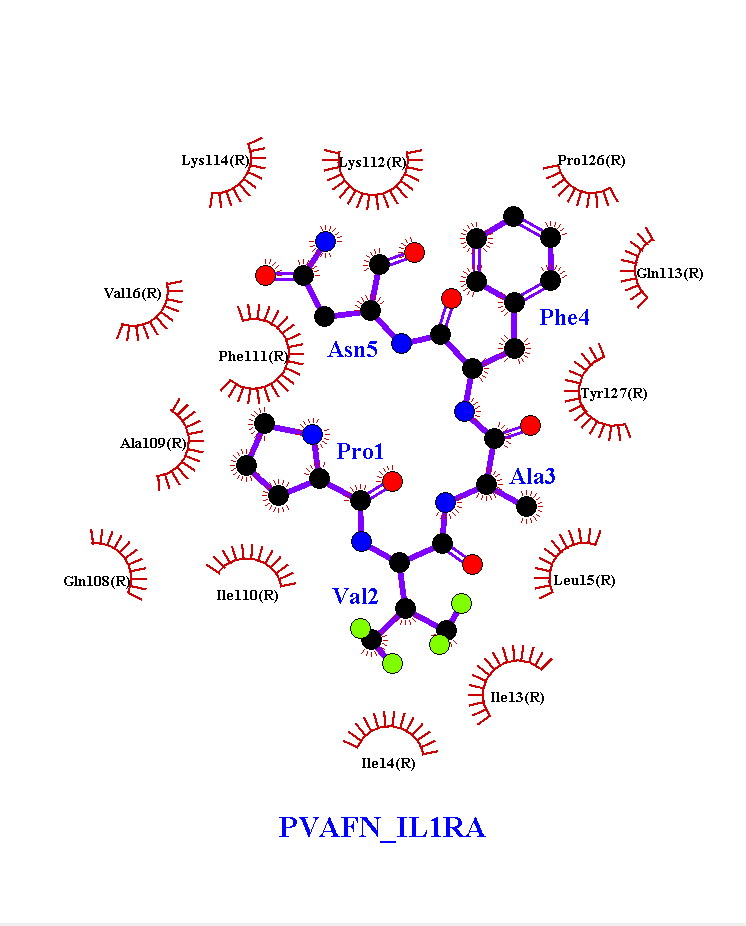

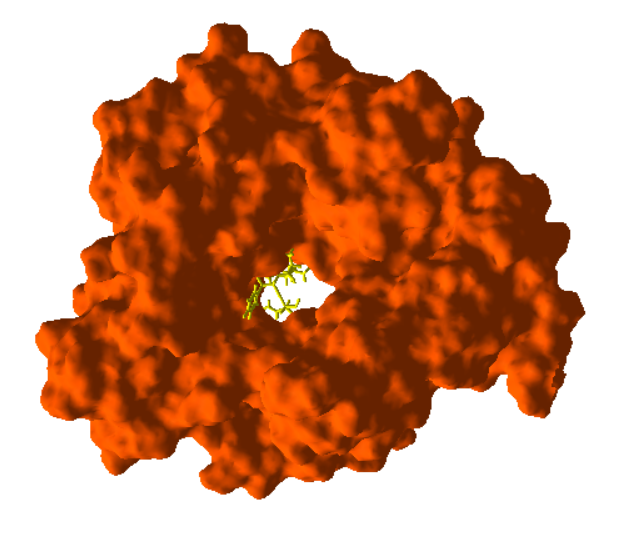


**Figure (2) PVAFN_IL1RA** Corresponding atoms and non-ligand Ile110, Gln108, Ala109, Val16, Phe111, Lys114, Lys112, Pro126, Gln113, Tyr127, Leu15, Ile13 and Ile14 involved in hydrophobic interactions.


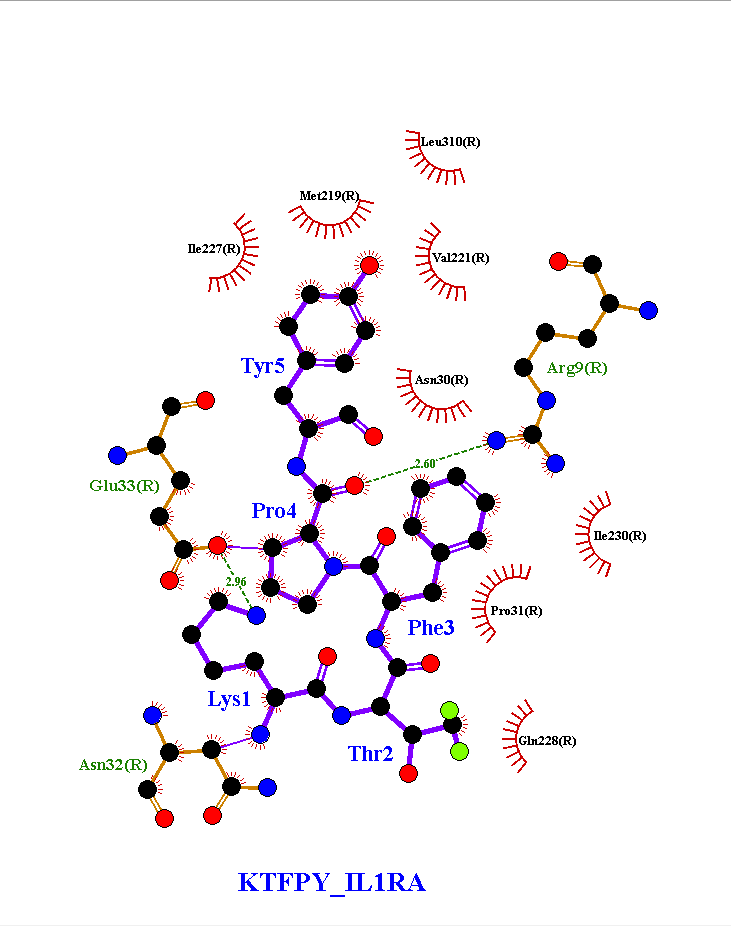

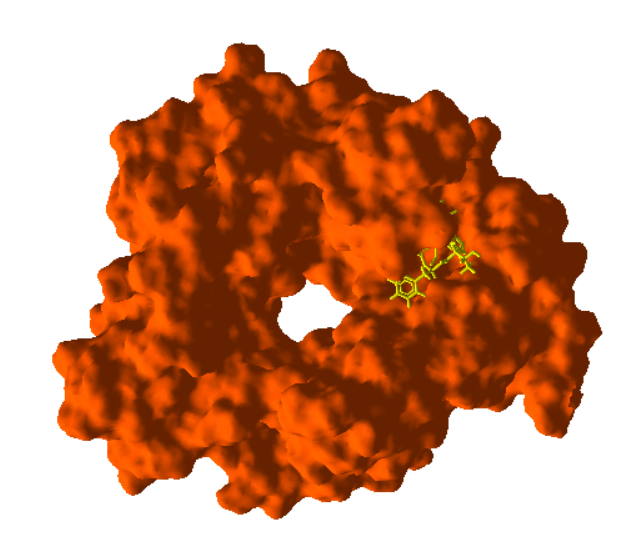


**Figure (3) KTFPY_IL1RA** Non-ligand bond. Corresponding atoms and non-ligand Gln228, Pro31, Ile230, Asn30, Val221, Leu310, Met219, Ile227 involved in hydrophobic interactions. Hydrogen bond between KTFPY and Asn32, Glu33 and Arg9 shown by dotted green lines.


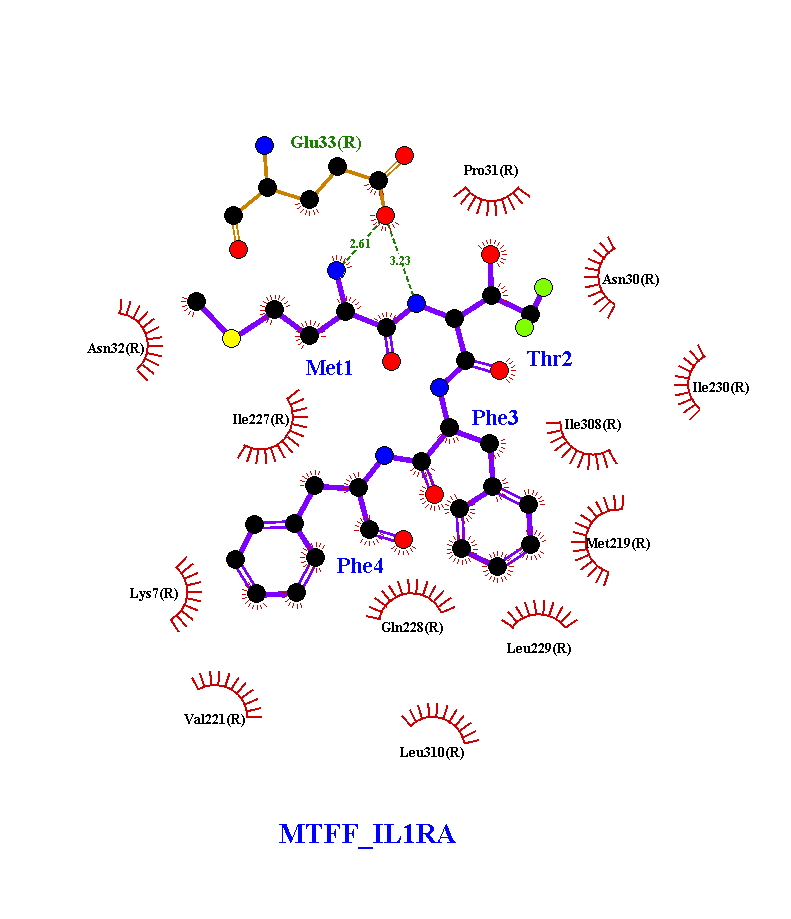

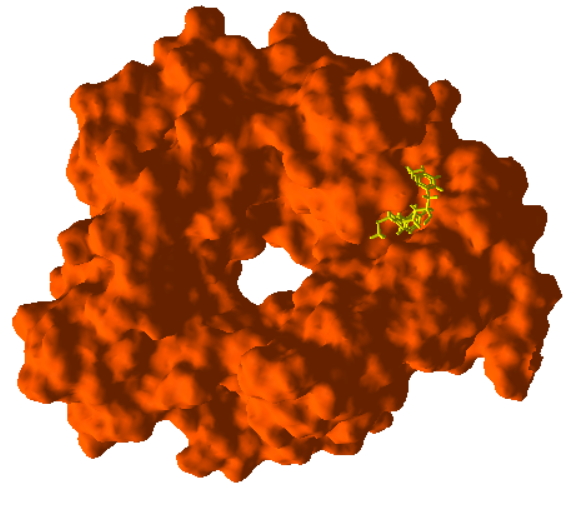


**Figure (4) MTFF_IL1RA** Non-ligand bond. Corresponding atoms and non-ligand Gn228, Leu310, Leu229, Met219, Ile308, Ile230, Asn30, Pro31, Asn32, Ile227, Lys7 and Val221 involved in hydrophobic interactions. Hydrogen bond between MTFF and Glu33 shown by dotted green lines.


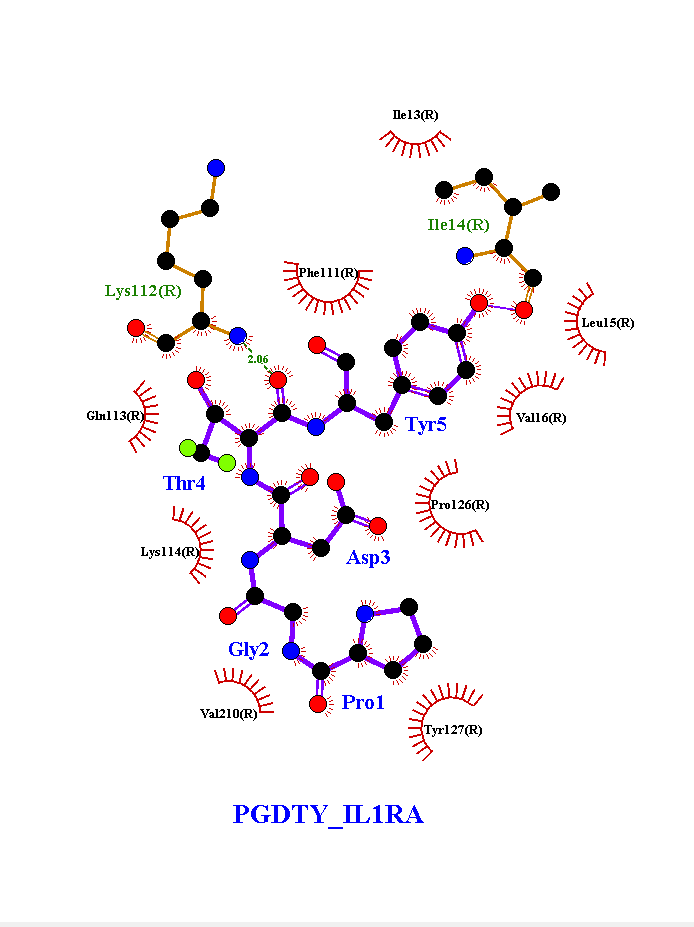

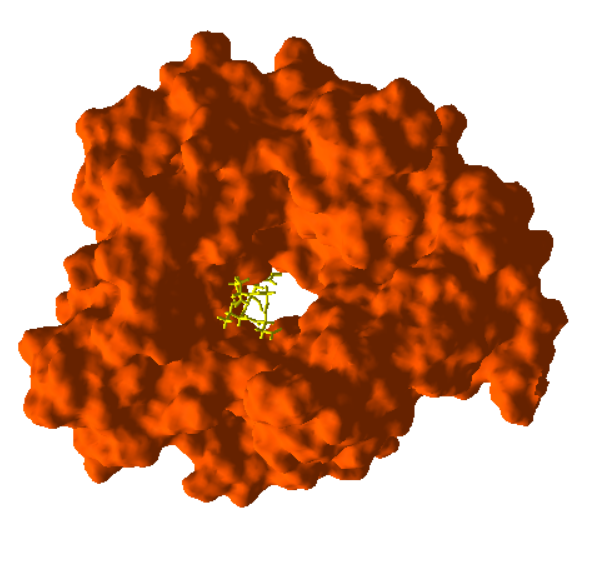


**Figure (5) PGDTY_IL1RA** Non-ligand bond. Corresponding atoms and non-ligand Tyr127, Pro126, Val126, Leu15, Ile13, Phe111, Gln113, Lys114 and Val210 involved in hydrophobic interactions. Hydrogen bond between PGTDY and Lys112 shown by dotted green lines.


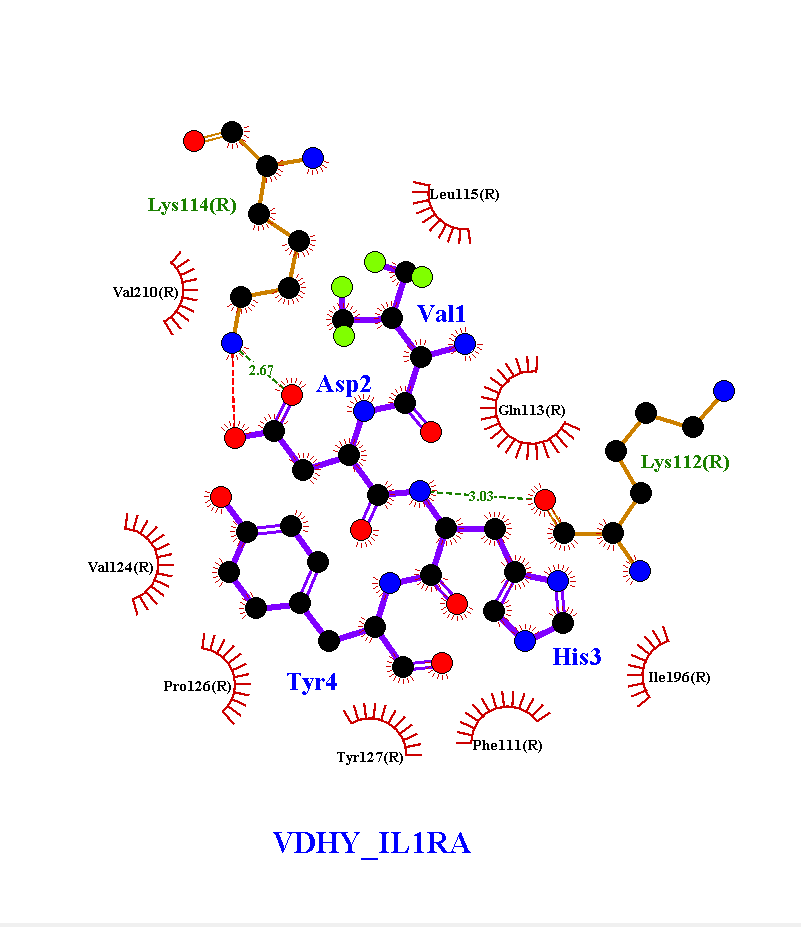

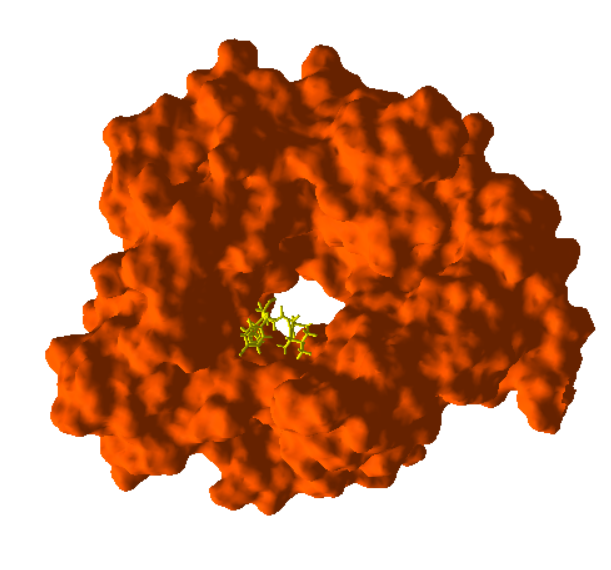


**Figure (6) VDHY_IL1RA** Non-ligand bond. Corresponding atoms and non-ligand Ile196, Phe111, Tyr127, Pro126, Val124, Vall210, Gn113 and Leu115 involved in hydrophobic interactions. Hydrogen bond between VDHY and Lys112 and Lys114 and salt bridges with Lys114 shown by dotted green lines.


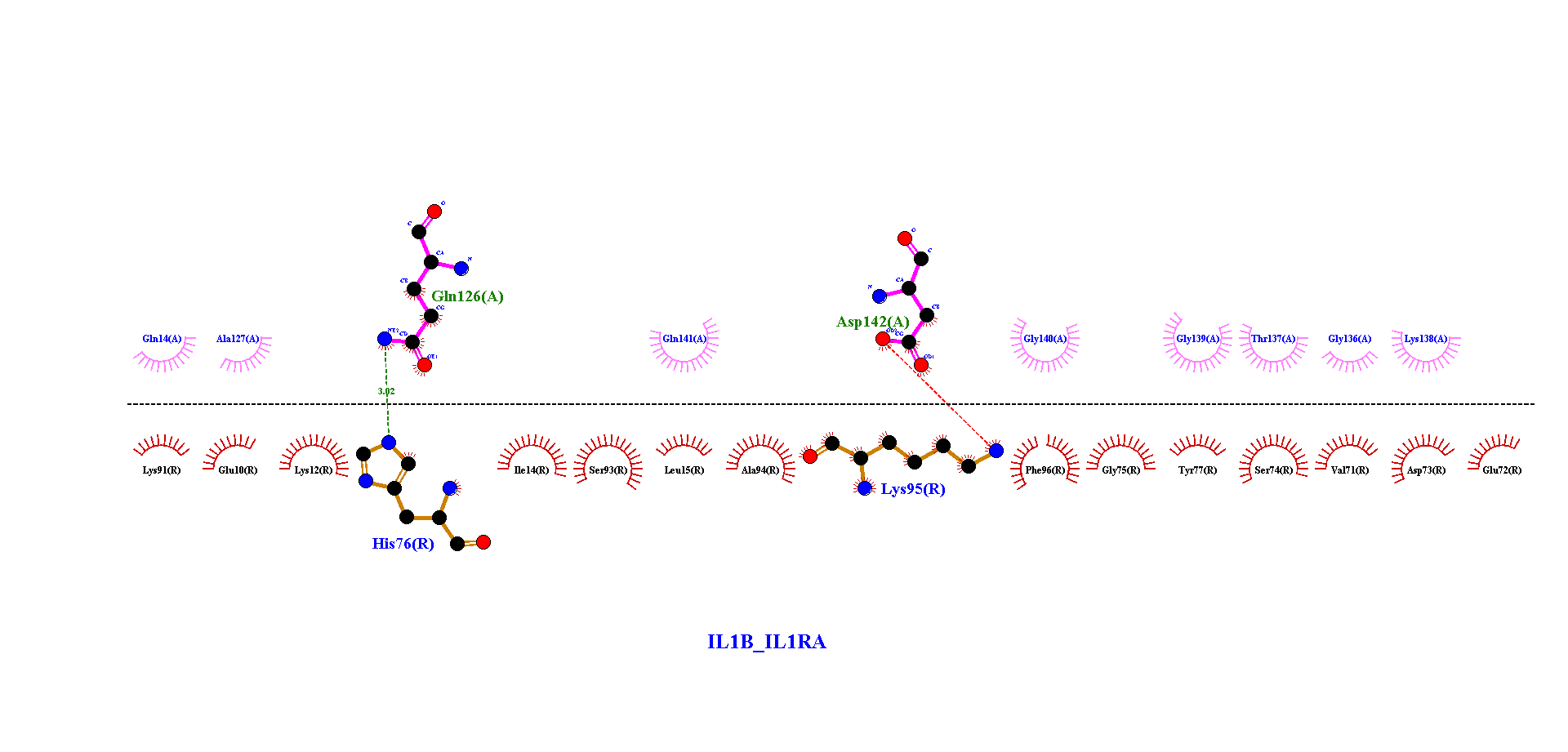

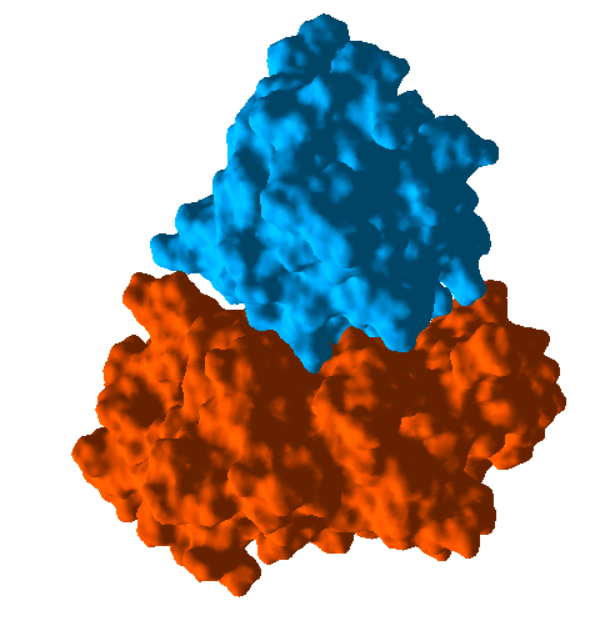


**Figure (7) IL1B_IL1RA** Gln14, Ala127, Gln141, Gly140, Gly139, Thr137, Gly136 and Lys138. Lys91, Glu10, Lys120, Ile14, Ser93, Leu15, Ala94, Phe96, Gly75, Try77, Ser74, Val71, Asp73 and Gln72Hydrogen bond between (Gln126 and His76) shown by dotted green lines. and salt bridges between (Asp142 and Lys95) shown by dotted red lines.


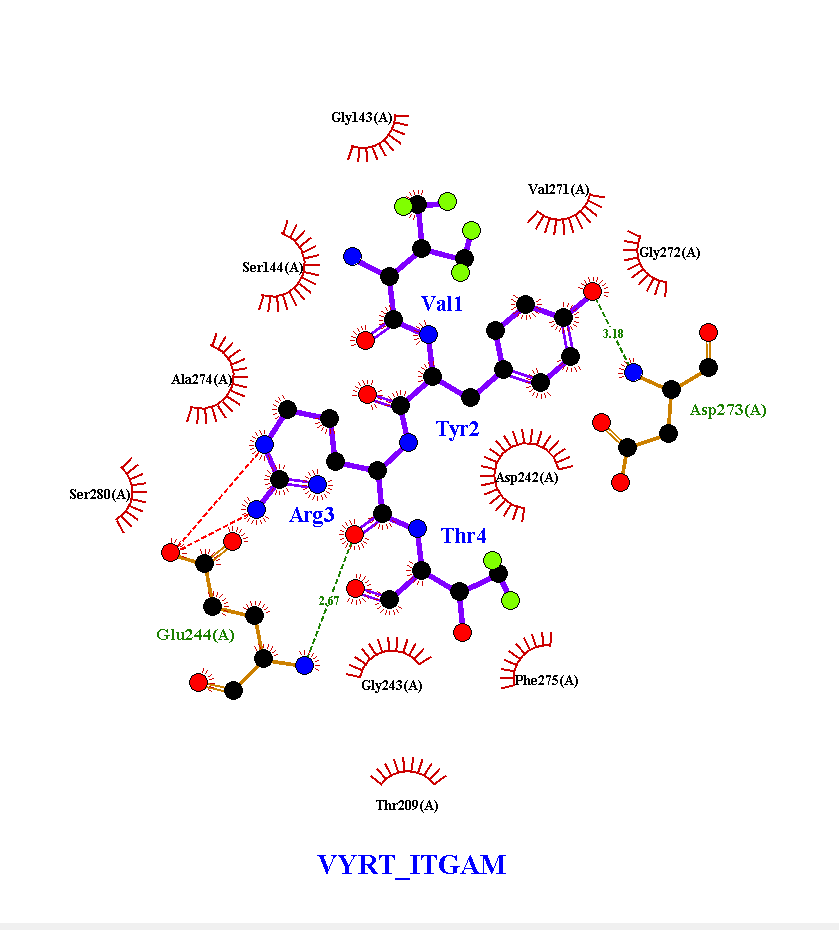

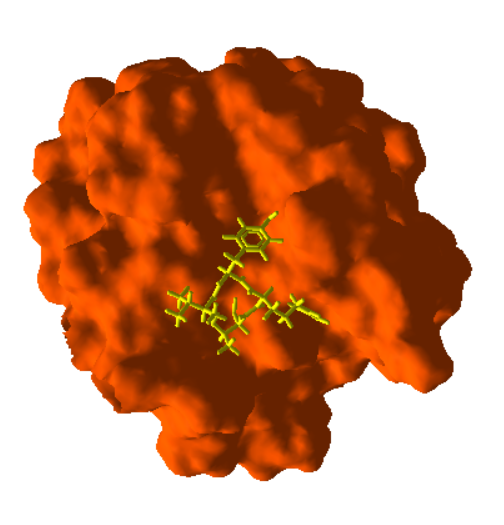


**Figure (1) VYRT_ITGAM** Non-ligand bond. Corresponding atoms and non-ligand Asp242, Phe275, Gly243, Thr209, Ser280, Ala274, Ser144, Gly143, Val271 and Gly272 involved in hydrophobic interactions. Hydrogen bond between VYRT and Glu244 and Asp273 and salt bridges with Glu244 shown by dotted green lines.


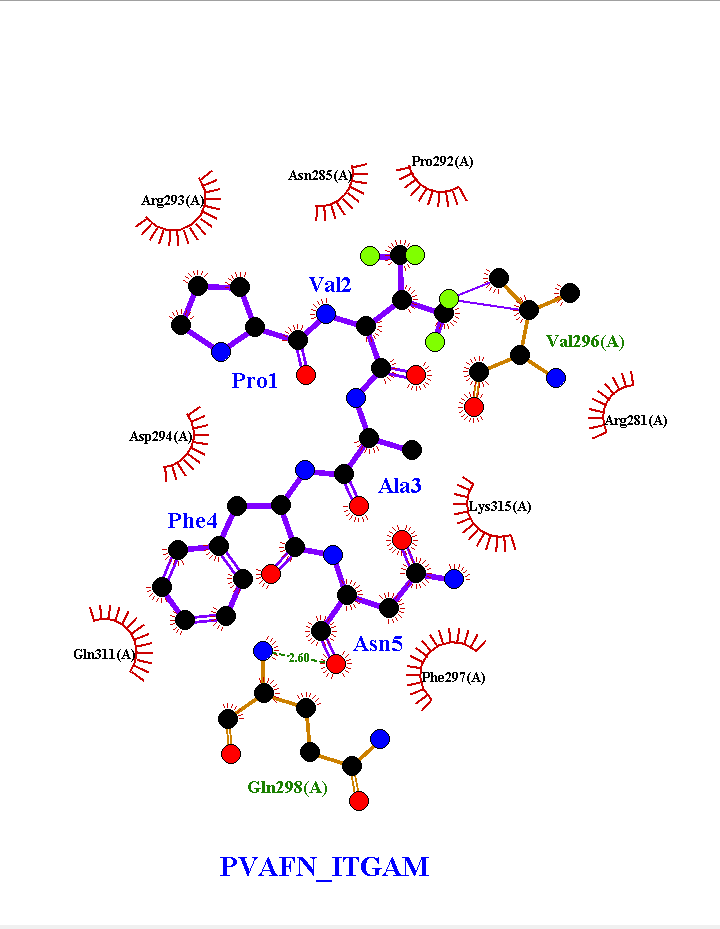

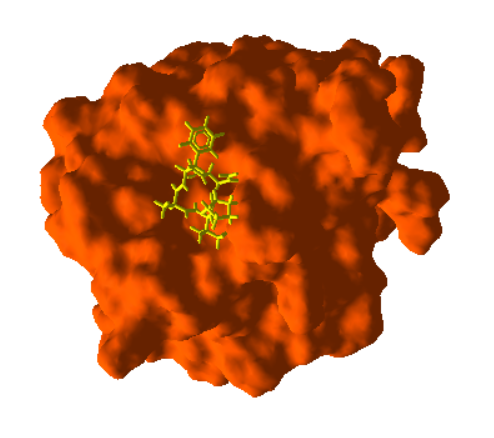


**Figure (2) PVAFN_ITGAM** Non-ligand bond. Corresponding atoms and non-ligand Arg281, Lys315, Phe297, Gln311, Asp294, Arg293, Asn285 and Pro292 involved in hydrophobic interactions. Hydrogen bond between PVAFN and Gln298 and external bonds with Val296 shown by dotted green lines.


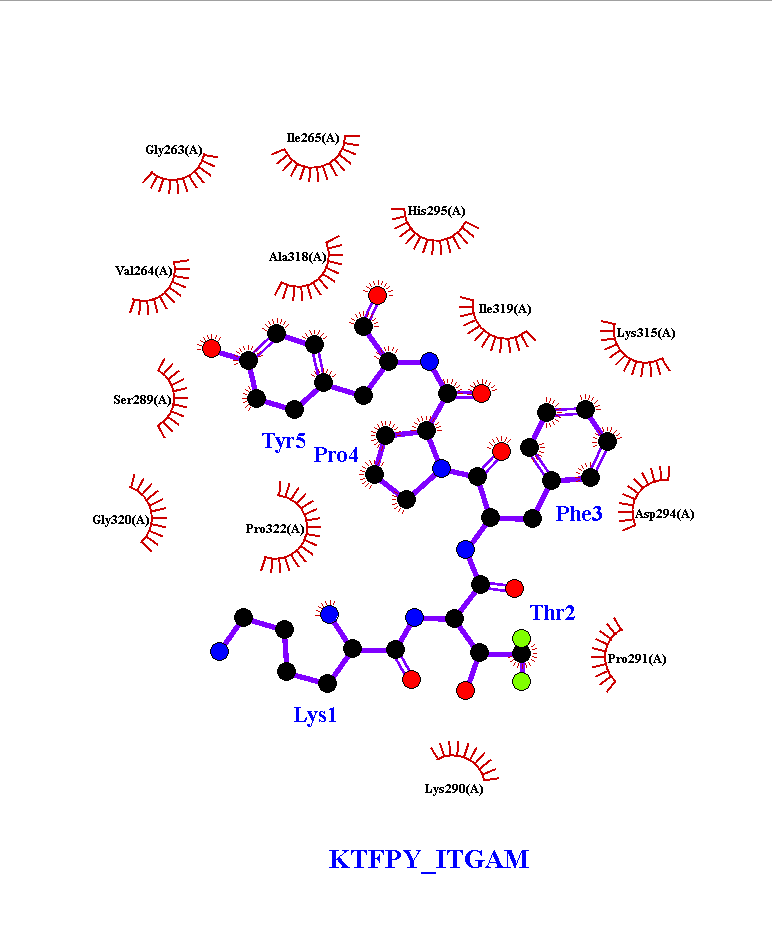

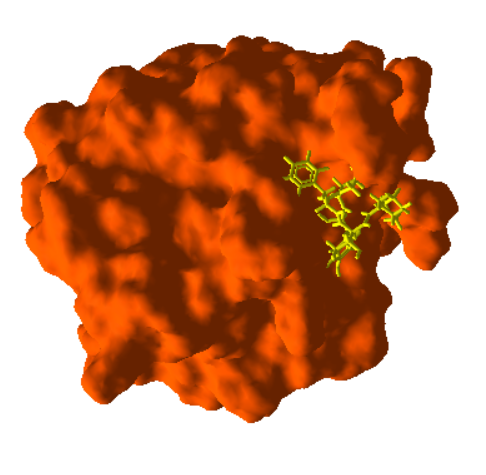


**Figure (3) KTFPY_ITGAM** Corresponding atoms and non-ligand Lys290, Pro291, Asn294, Lys315, Ile319, His295, Ile265, Gly263, Val243, Ala318, Ser289, Gly320 and Pro322 involved in hydrophobic interactions.


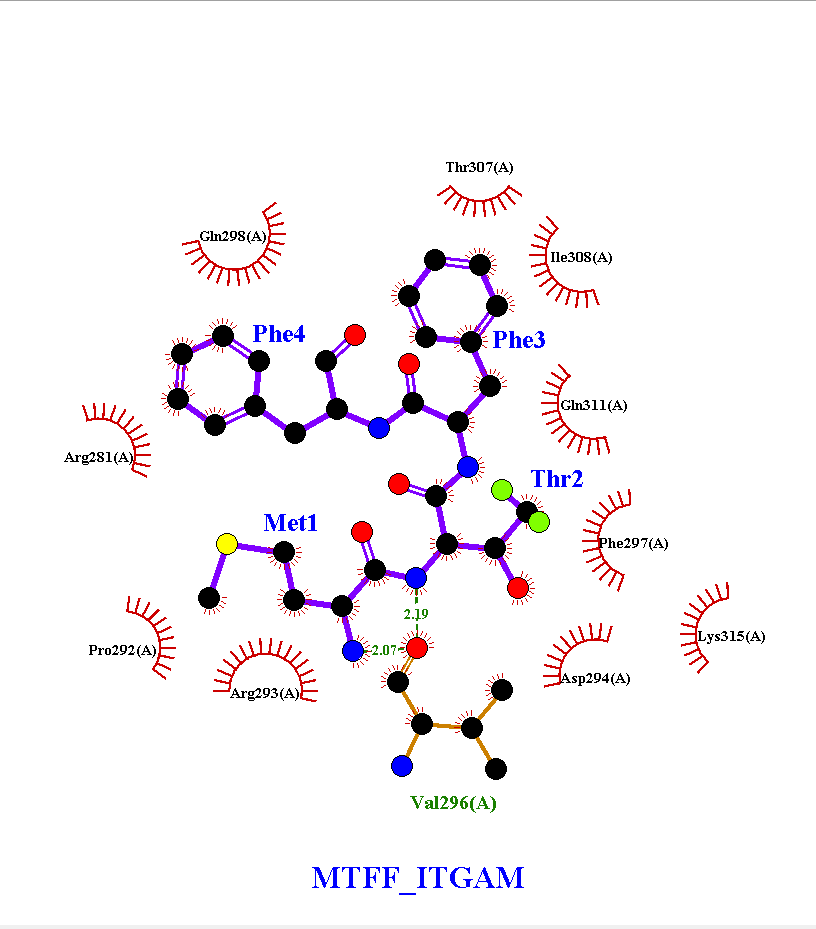

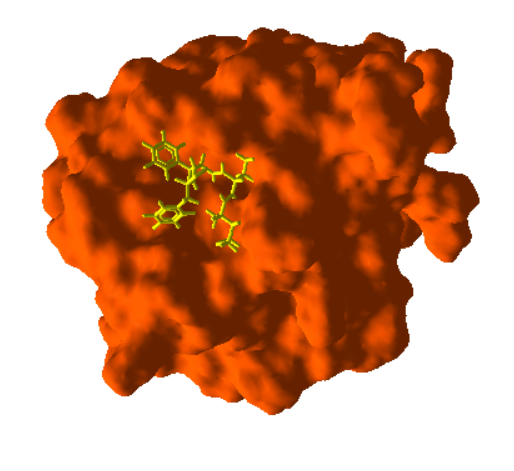


**Figure (4) MTFF_ITGAM** Non-ligand bond. Corresponding atoms and non-ligand Asp294, Lys315, Phe297, Gln311, Ile308, Thr307, Gln298, Arg281, Pro292 and Arg293 involved in hydrophobic interactions. Hydrogen bond between MTFF and Val296 shown by dotted green lines.


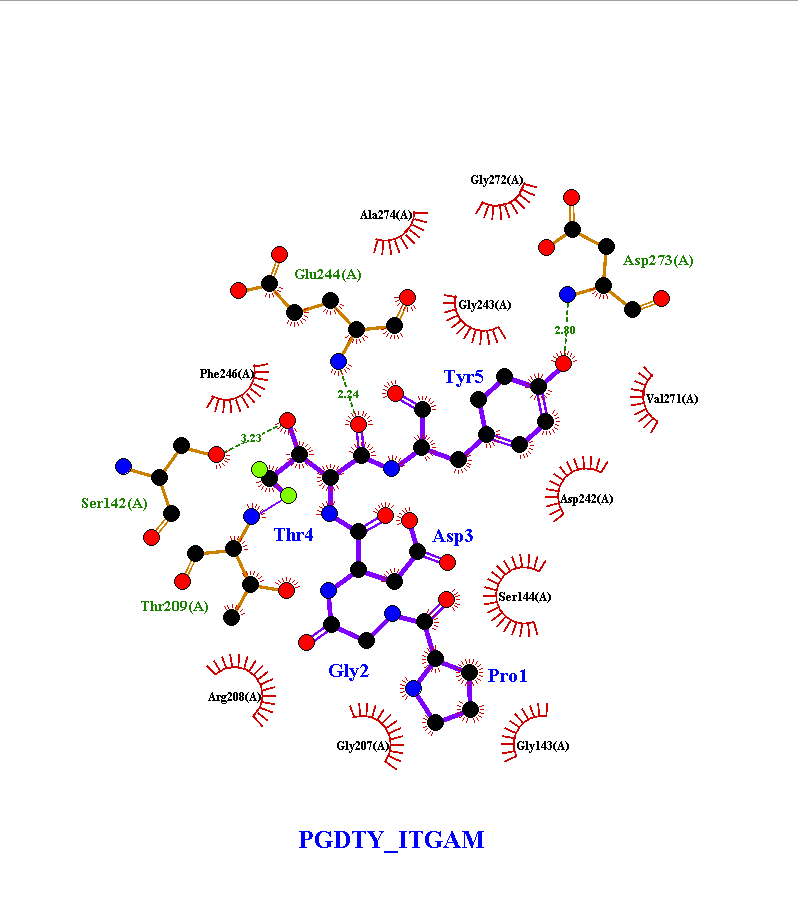

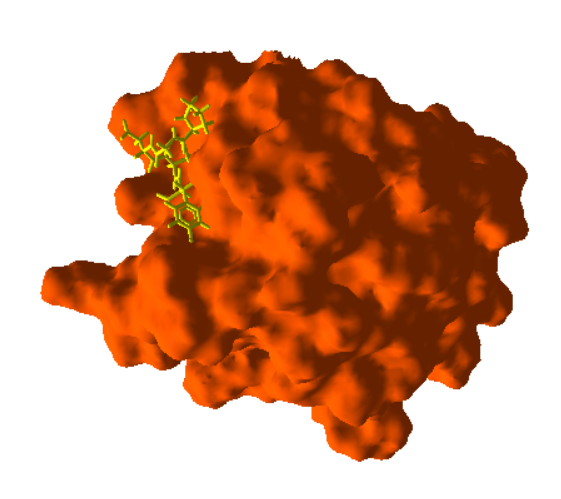


**Figure (5) PGDTY_ITGAM** Non-ligand bond. Corresponding atoms and non-ligand Gly143, Ser14, Asp242, Val271, Gly272, Gly243, Ala274, Phe246, Arg208 and Gly207 involved in hydrophobic interactions. Hydrogen bond between PGDTY and Asp273 and Glu24 and Ser142 and external bond with Thr209 shown by dotted green lines.


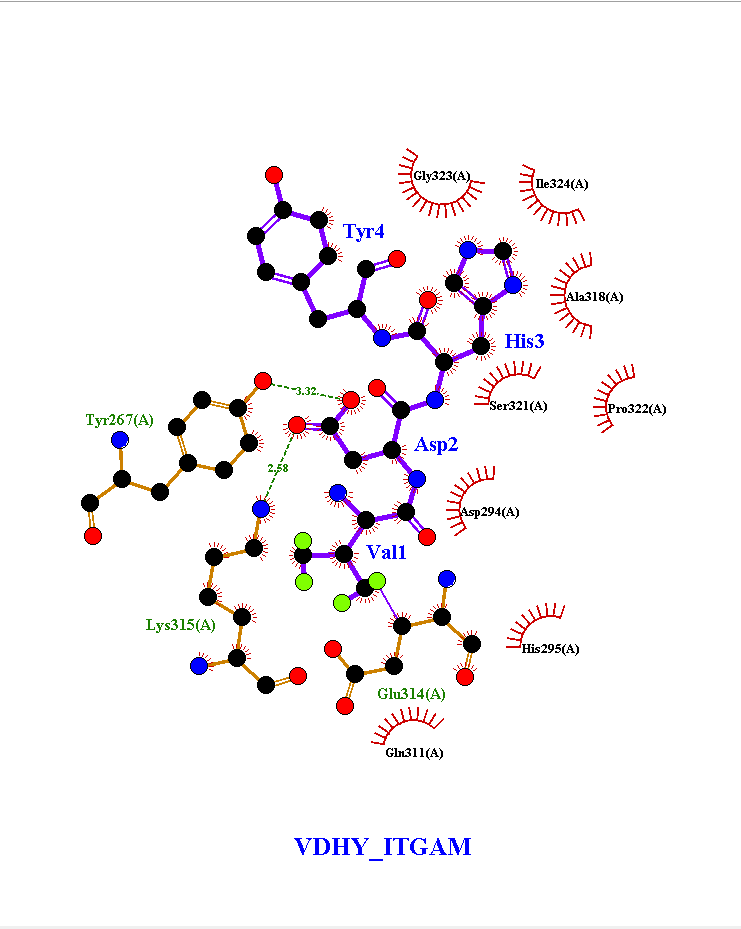

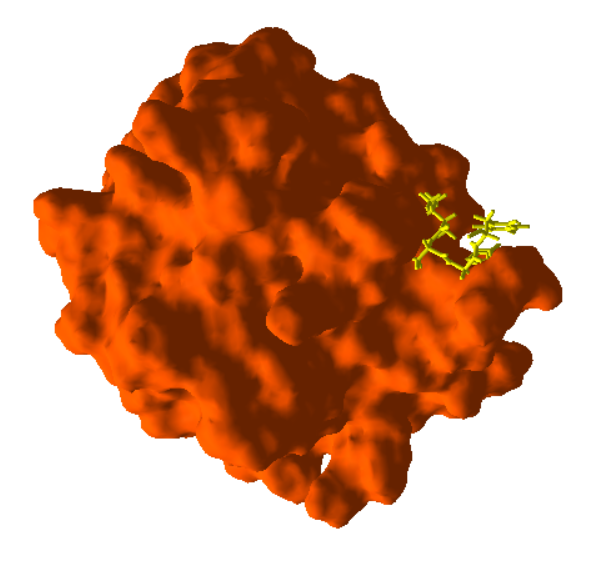


**Figure (6) VDHY_ITGAM** Non-ligand bond. Corresponding atoms and non-ligand Gln311, His295, Asp294, Ser321, Pro322, Ala318, Ile324 and Gly323 involved in hydrophobic interactions. Hydrogen bond between VDHY and Lys315 and Tyr267 shown by dotted green lines.

------------------------------------------------------------------------------------------------


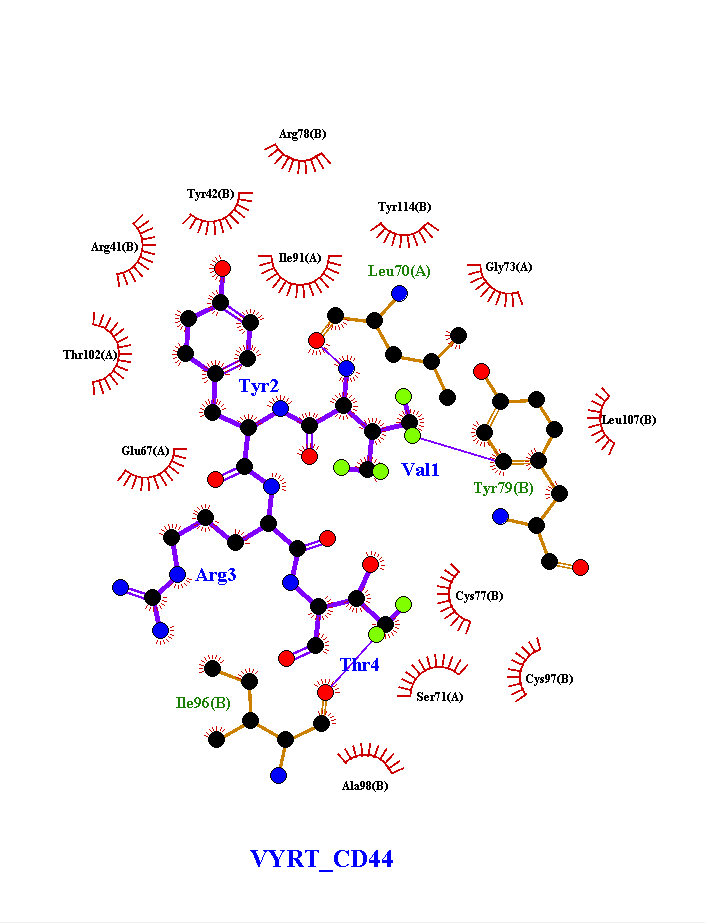

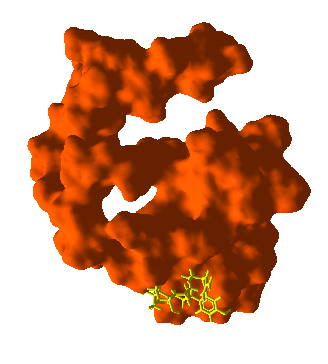


**Figure (1) VYRT_CD44** Non-ligand bond. Corresponding atoms and non-ligand Ala98, Ser71, Cys97, Cys77, Leu107, Gly73, Tyr114, Ile91, Tyr42, Arg78, Arg41, Thr102 and Glu67 involved in hydrophobic interactions. External bonds between Ile96 and Leu70 and Tyr79 shown by dotted purple lines.


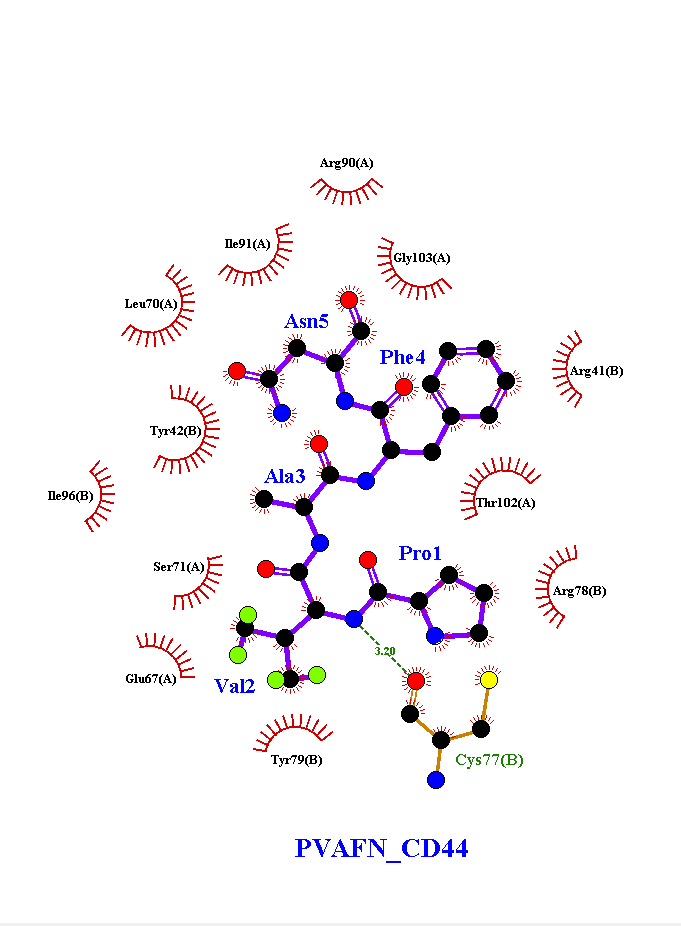

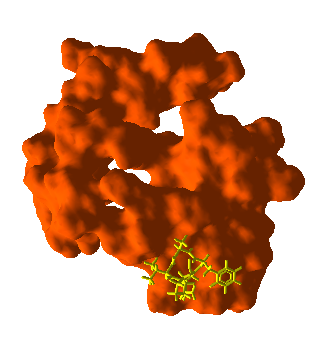


**Figure (2) PVAFN_CD44** Non-ligand bond. Corresponding atoms and non-ligand Arg78, Thr102, Arg41, Gly103, Arg90, Ile91, Leu70, Tyr42, Ile96, Ser71, Ser71 and Glu67 involved in hydrophobic interactions. Hydrogen bonds between PVAFV and Cys77 shown by dotted green lines.


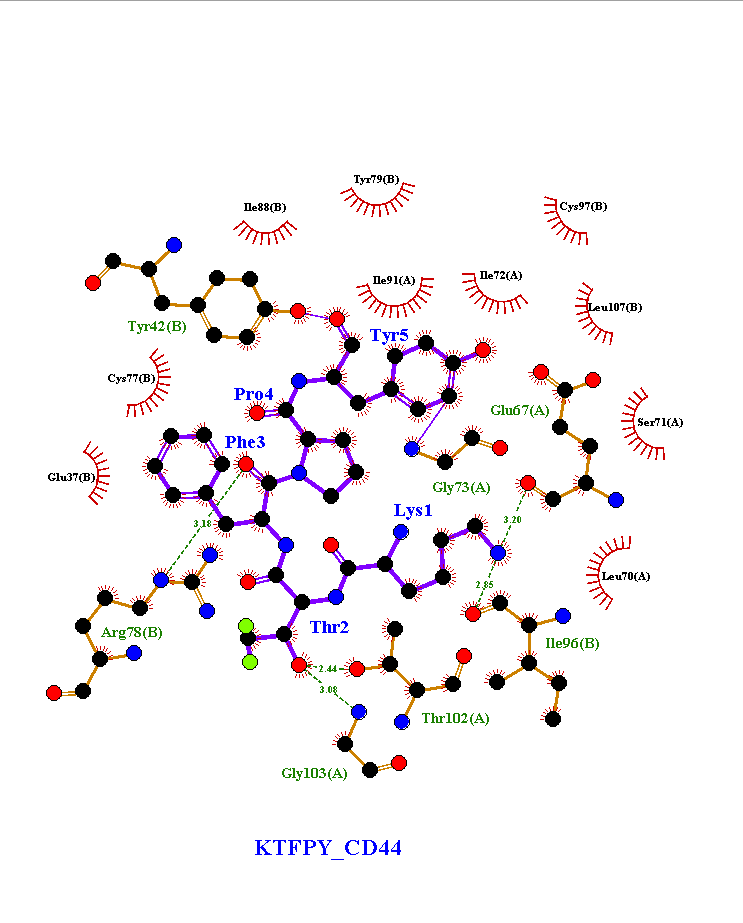

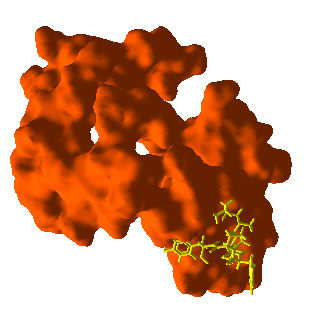


**Figure (3) KTFPY_CD44** Non-ligand bond. Corresponding atoms and non-ligand Arg78, Thr102, Arg41, Gly103, Arg90, Ile91, Leu70, Tyr42, Ile96, Ser71, Ser71 and Glu67 involved in hydrophobic interactions. Hydrogen bonds between KTFPY and Ile96, Glu67, Thr102, Gly103 and Arg78 and external bonds with Gly73 and Tyr42 shown by dotted green lines.


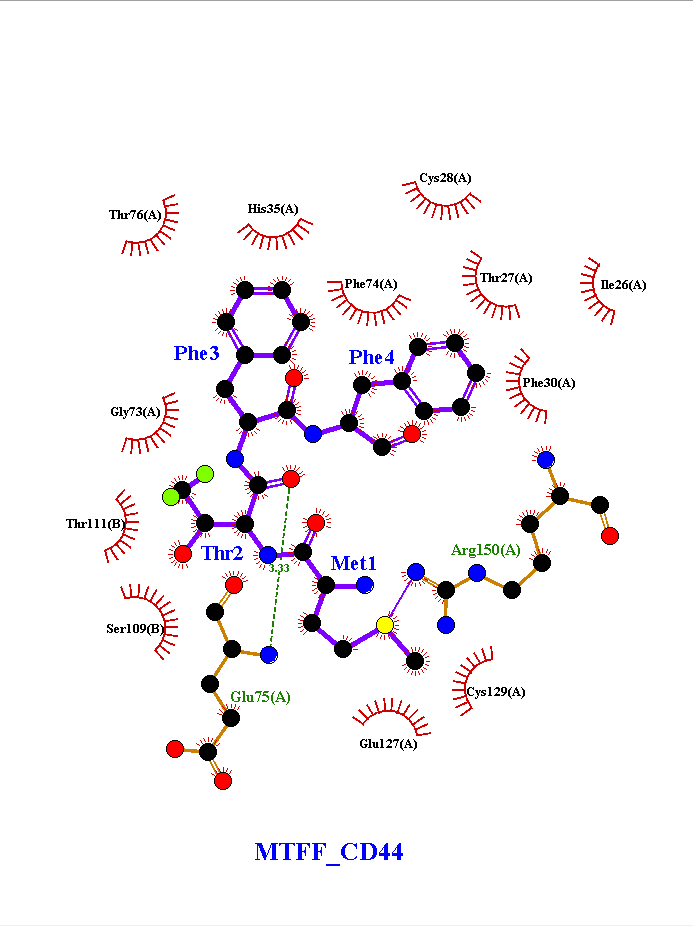

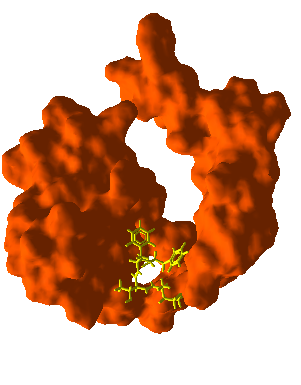


**Figure (4) MTFF_CD44** Non-ligand bond. Corresponding atoms and non-ligand Glu127, Cys129, Phe30, Ile26, Thr27, Phe74, Cys28, His35, Thr76, Gly73, Thr111 and Ser109 involved in hydrophobic interactions. Hydrogen bonds between KTFPY and Glu75 and external bond with Arg150 shown by dotted green lines.


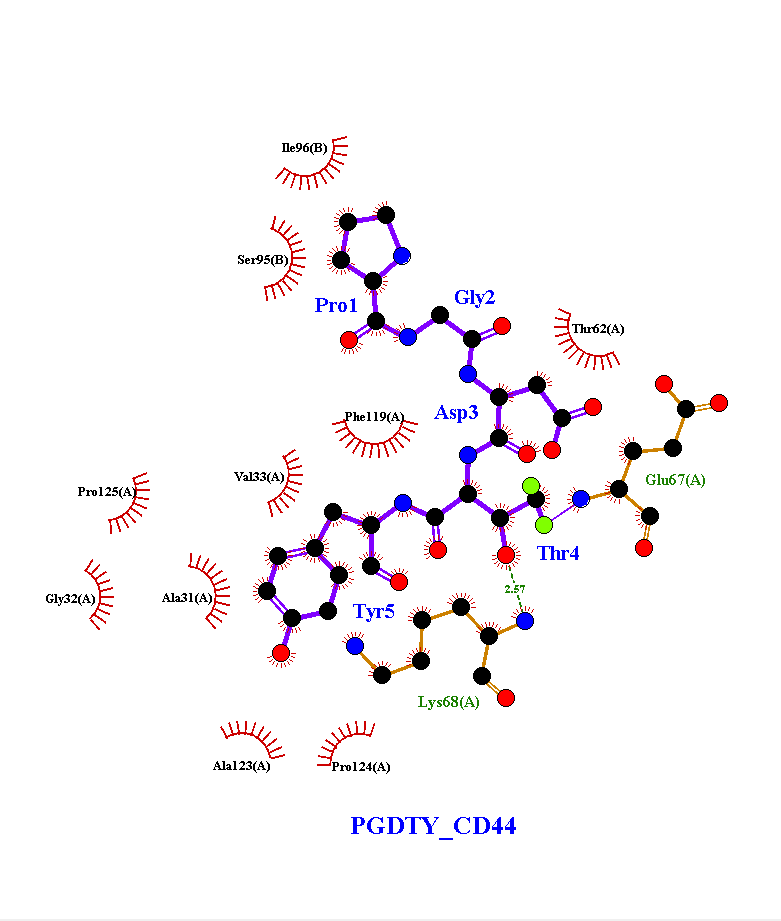

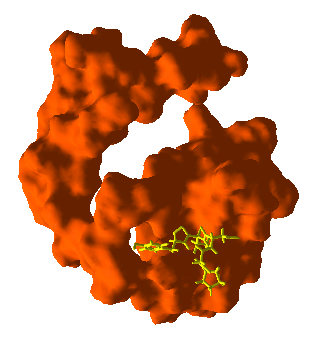


**Figure (5) PGDTY_CD44** Non-ligand bond. Corresponding atoms and non-ligand Pro124, Ala123, Ala31, Gly32, Pro125, Val33, Phe119, Ser95, Ile96 and Thr62 involved in hydrophobic interactions. Hydrogen bonds between PGDTY and Lys68 and external bond with Glu67 shown by dotted green lines.


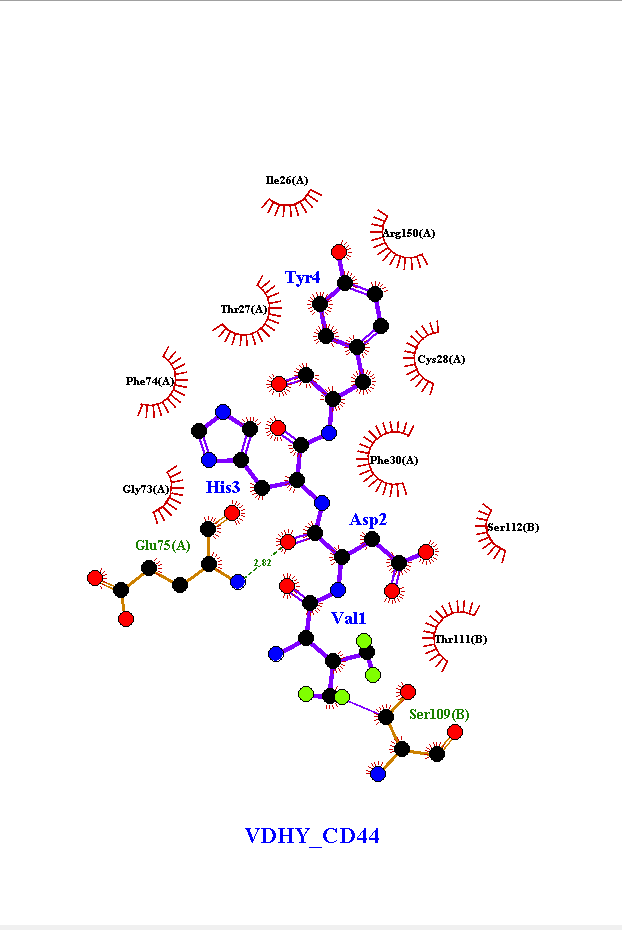

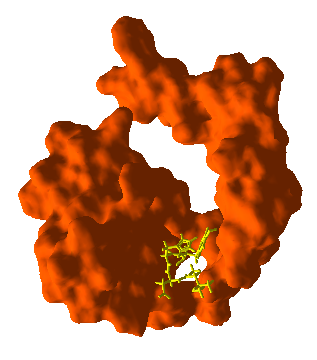


**Figure (6) VDHY_CD44** Non-ligand bond. Corresponding atoms and non-ligand Thr111, Ser112, Phe30, Cys28, Arg140, Thr27, Phe74 and Gly73 involved in hydrophobic interactions. Hydrogen bonds between PGDTY and Lys68 and external bond with Glu67 shown by dotted green lines.


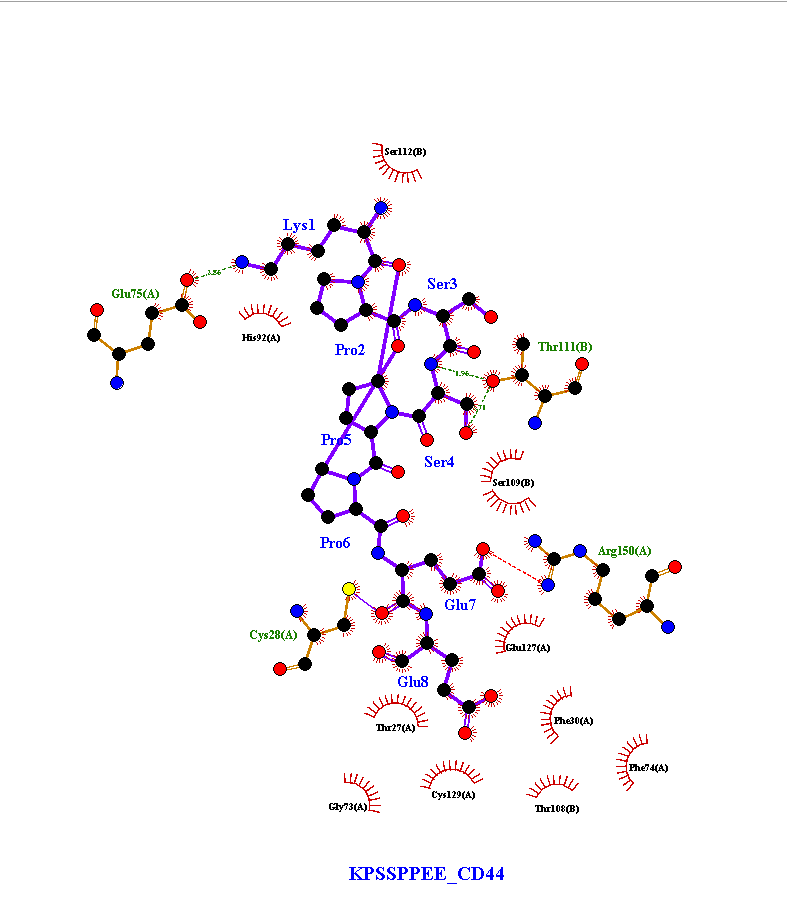

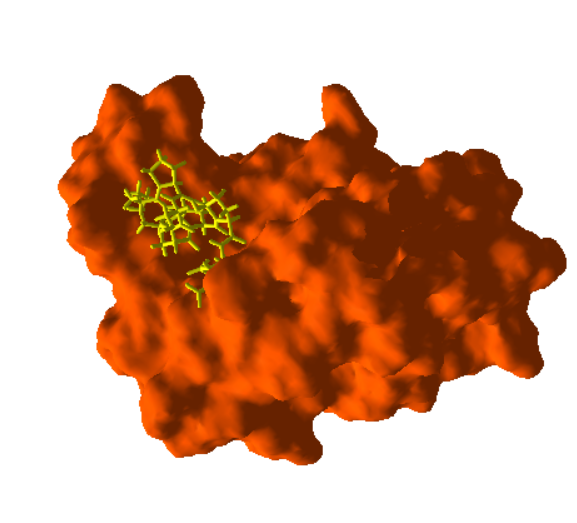


**Figure (1) VDHY_CD44** Non-ligand bond. Corresponding atoms and non-ligand Glu127, Phe30, Phe74, Thr108, Cys129, Thr27, Gly73, His92, Ser112 and Ser109 involved in hydrophobic interactions. Hydrogen bonds between PGDTY and Thr111 and Glu75 and salt bridges with Arg150 shown by dotted green lines.


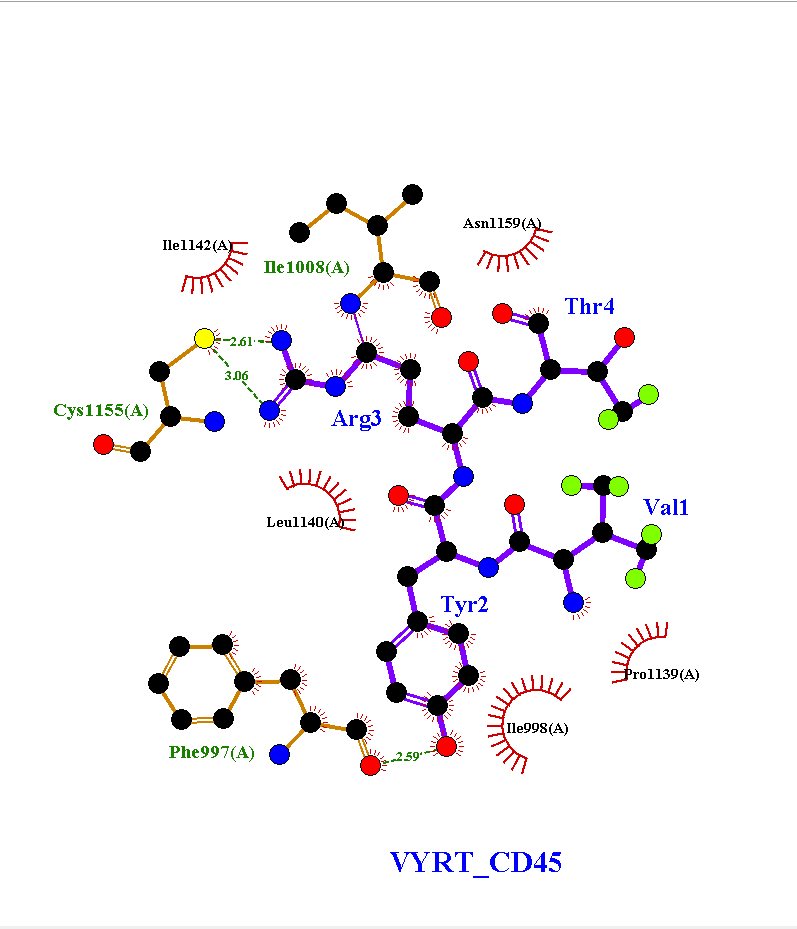

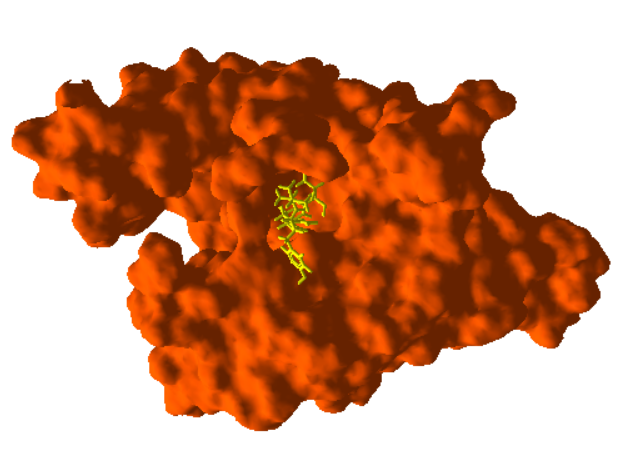


**Figure (1) VYRT_CD45** Non-ligand bond. Corresponding atoms and non-ligand Pro1139, Ile998, Leu1140, Ile1142 and Asn1159 involved in hydrophobic interactions. Hydrogen bonds between VYRT and Phe997 and Cys115 and external bond with Ile1008 shown by dotted green lines.


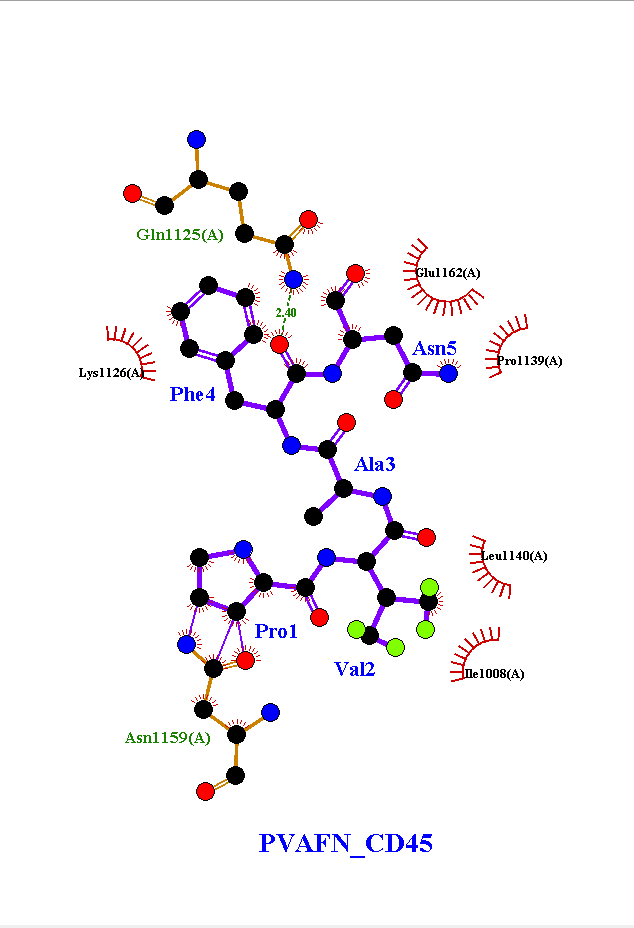

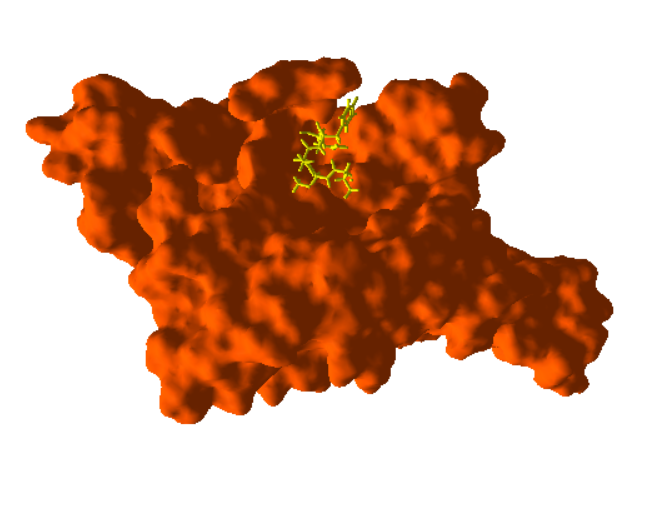


**Figure (2) PVAFN_CD45** Non-ligand bond. Corresponding atoms and non-ligand Ile1008, Leu1140, Pro1139, Glu1162and ys1126 involved in hydrophobic interactions. Hydrogen bonds between PVAFN and Gln1125 and external bond with Asn1159 shown by dotted green lines.


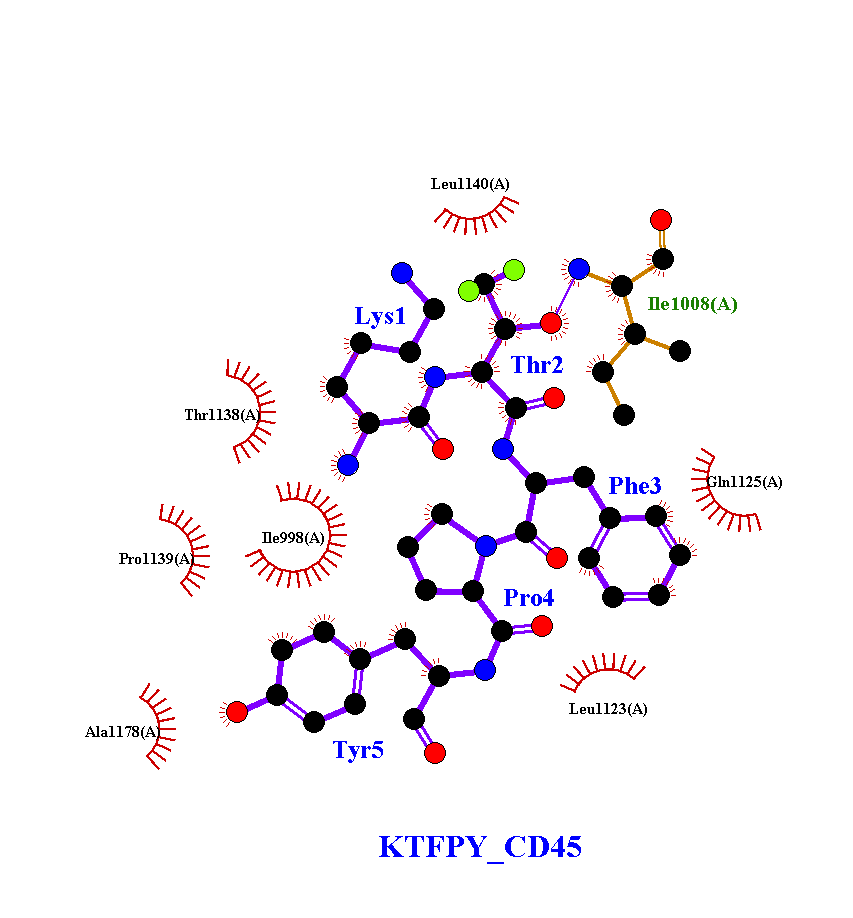

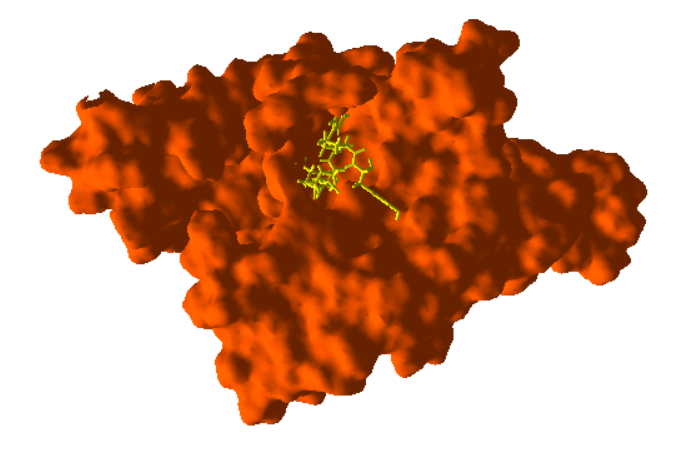


**Figure (3) KTFPY_CD45** Non-ligand bond. Corresponding atoms and non-ligand Leu1123, Gln125, Leu1140, Thr1138, Ile998, Pro1139 and Ala1178 involved in hydrophobic interactions. External bond between KTFPY and Ile1008 shown by dotted purple lines.


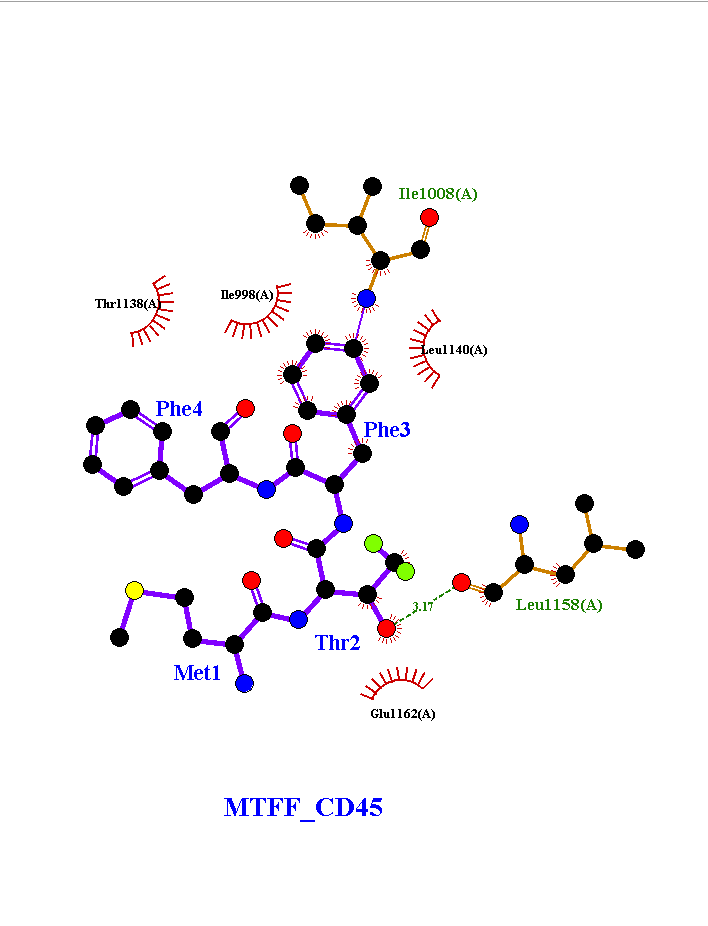

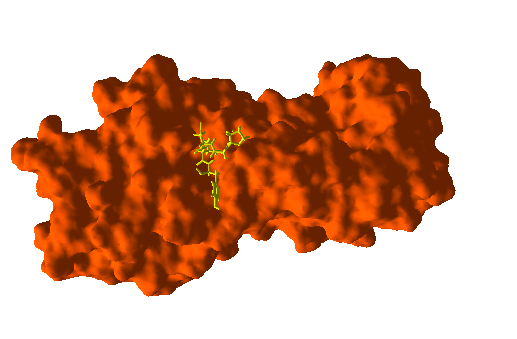


**Figure (4) MTFF_CD45** Non-ligand bond. Corresponding atoms and non-ligand Glu1162, Leu1140, Ile998 and Thr1138 Ala1178 involved in hydrophobic interactions. Hydrogen bond between MTFF and Leu1158 shown by dotted green lines.


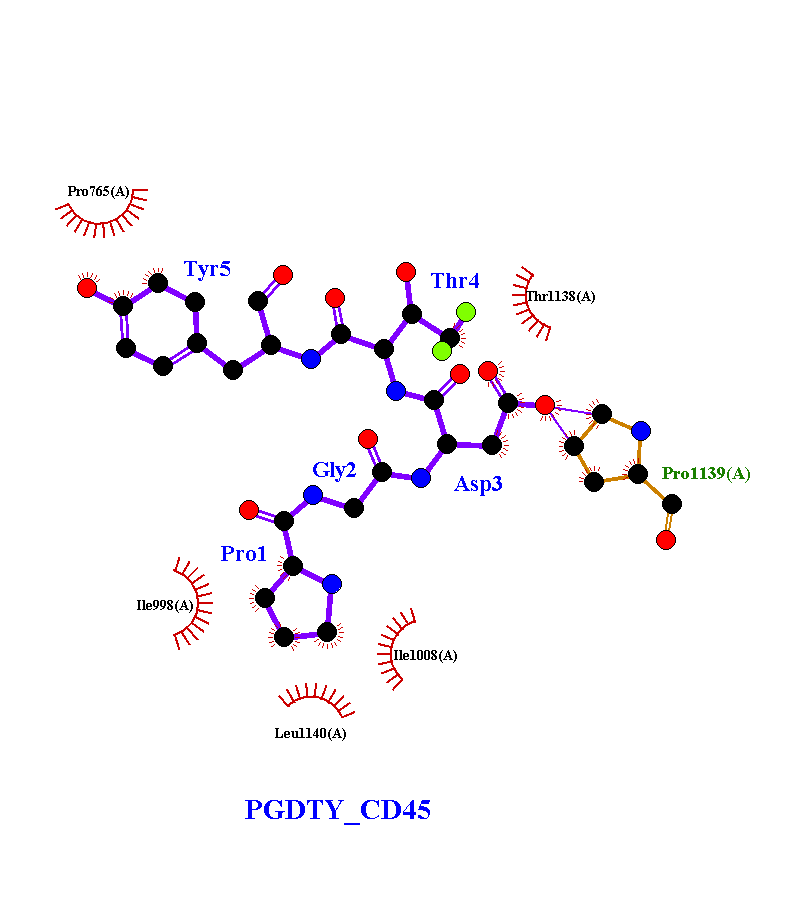

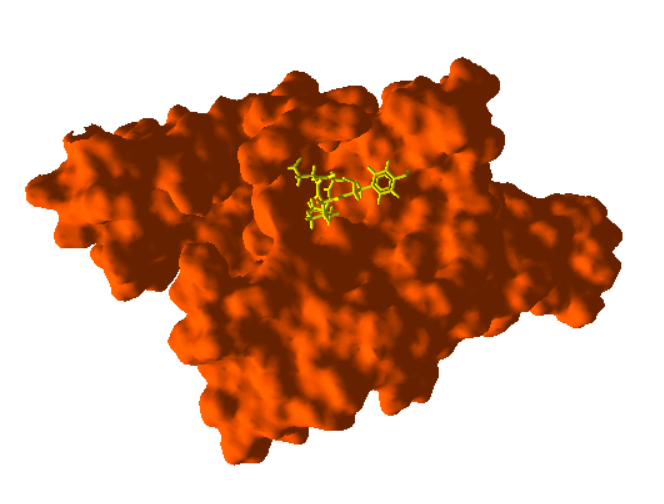


**Figure (5) PGDTY_CD45** Non-ligand bond. Corresponding atoms and non-ligand Ile1008, Leu1140, Ile998, Thr1138 and Pro765 involved in hydrophobic interactions. External bond between PGTDY and Pro1139 shown by dotted purple lines.


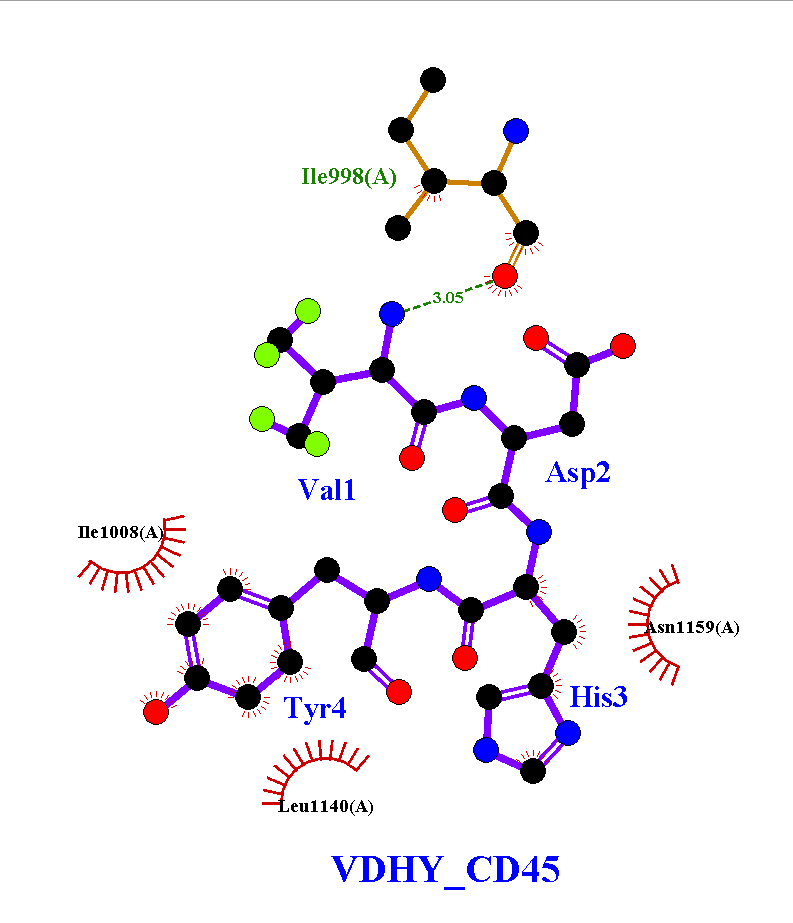

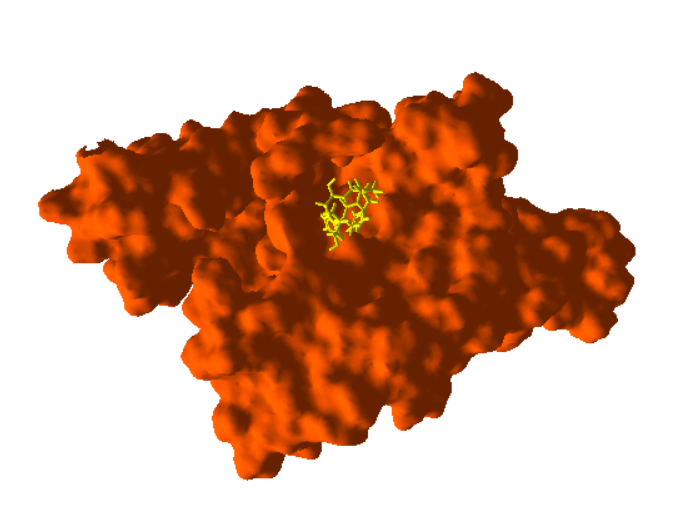


**Figure (5) VDHY_CD45** Non-ligand bond. Corresponding atoms and non-ligand Asn1159, Leu1140 and Ile1008 involved in hydrophobic interactions. Hydrogen bond between VDHY and Ile998 shown by dotted green lines.


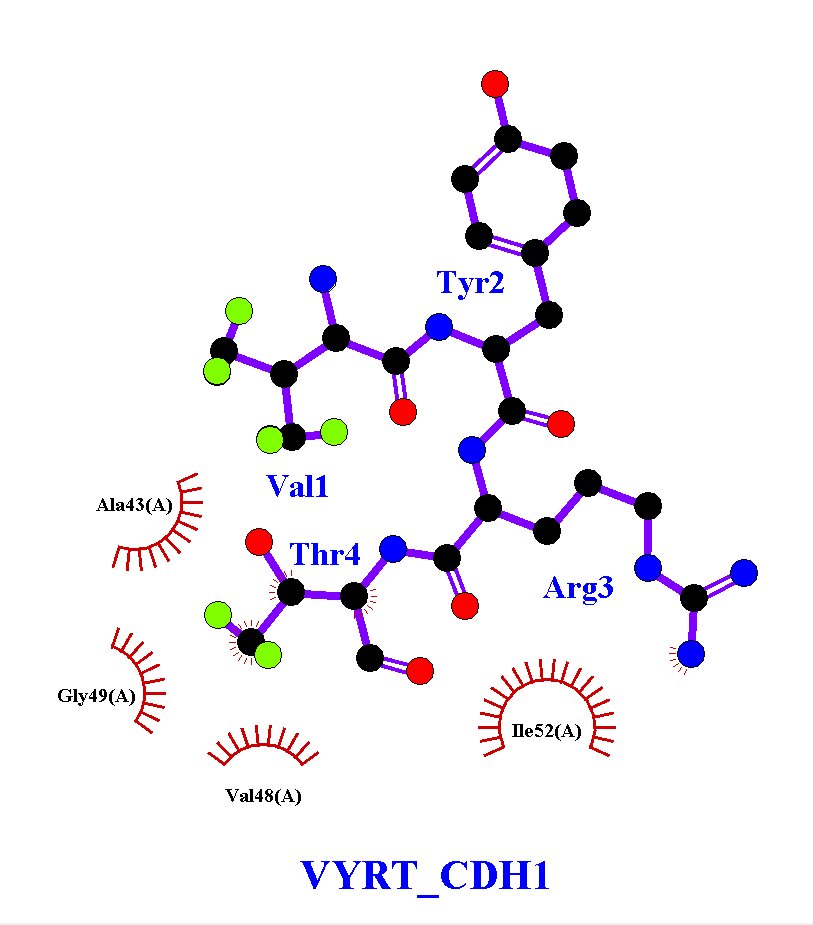

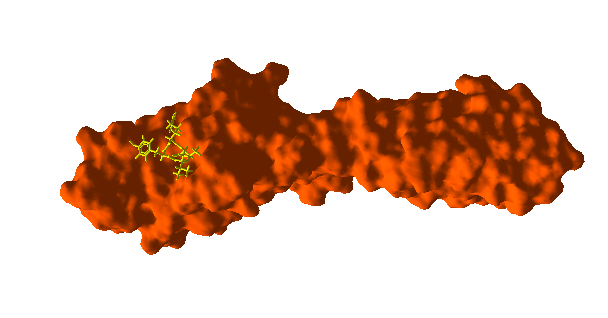


**Figure (1) VYRT_CDH1** Corresponding atoms and non-ligand Ile52, Val148, Gly49 and Ala43 involved in hydrophobic interactions.


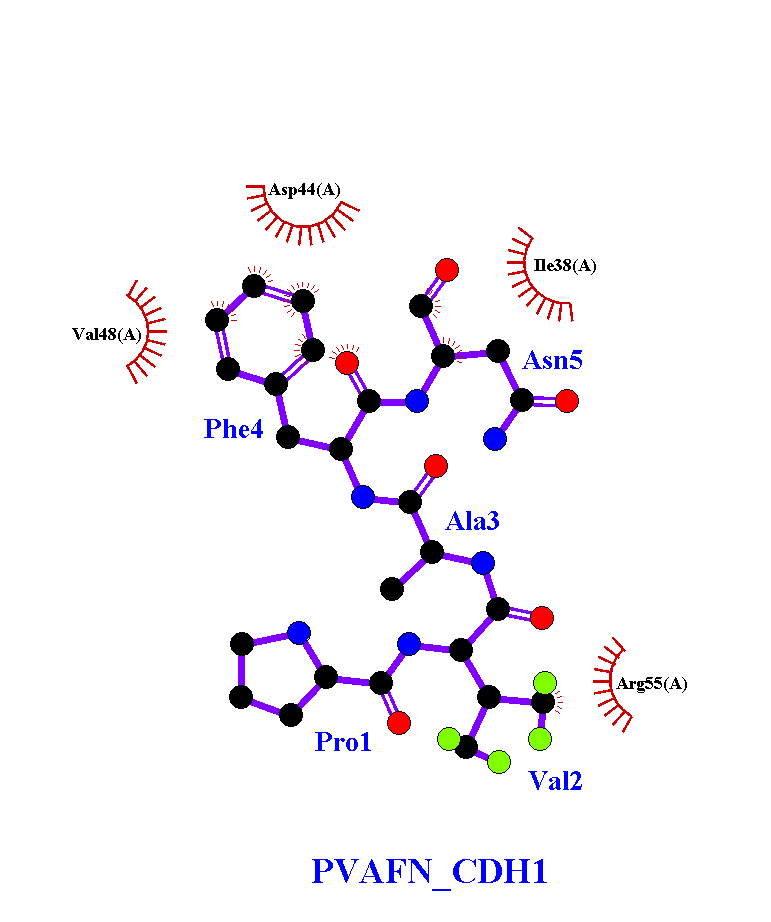

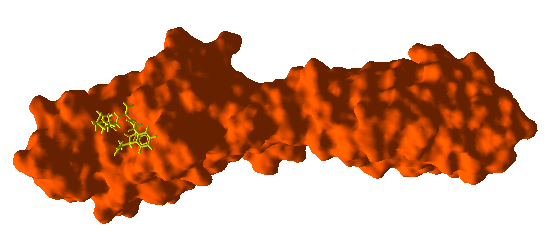


**Figure (2) PVAFN_CDH1** Corresponding atoms and non-ligand Arg55, Val48, Asp44 and Ile38 involved in hydrophobic interactions.


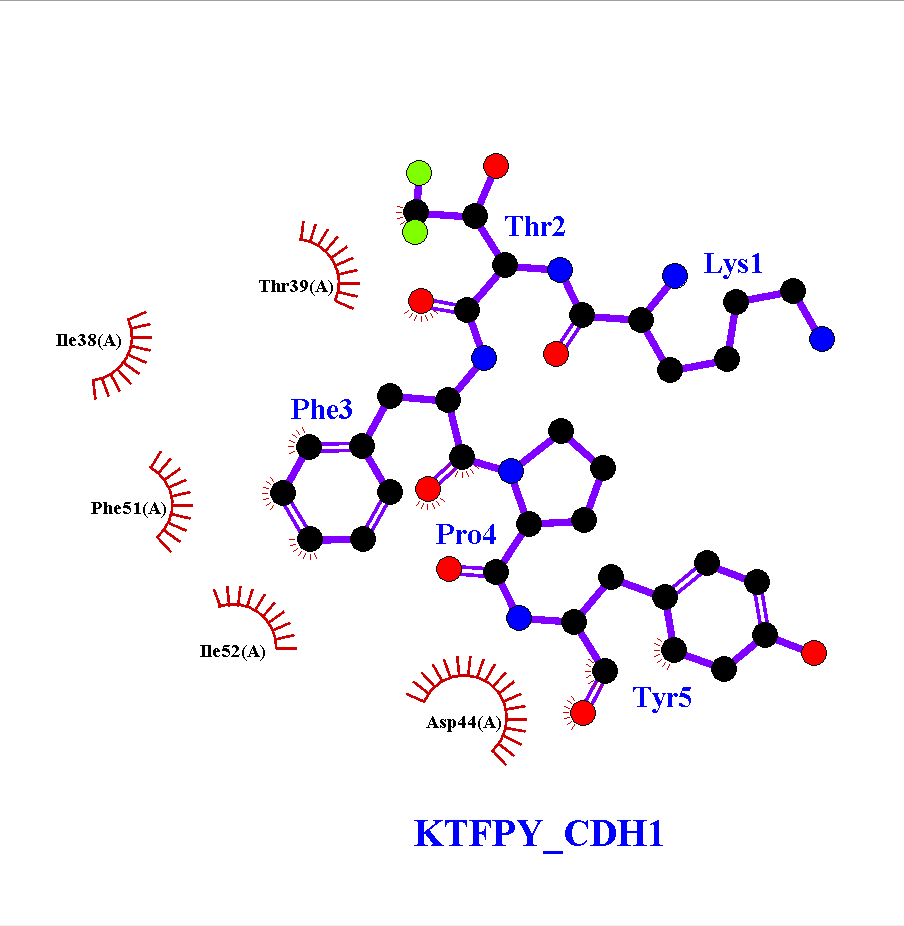

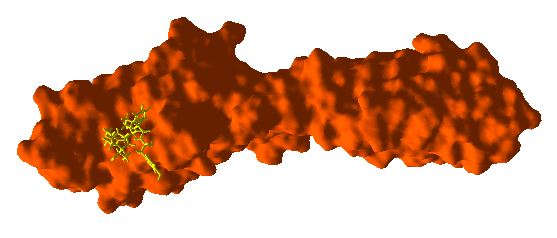


**Figure (3) KTFPY_CDH1** Corresponding atoms and non-ligand Asp4, Ile52, Phe51, Ile38 and Thr39 involved in hydrophobic interactions.


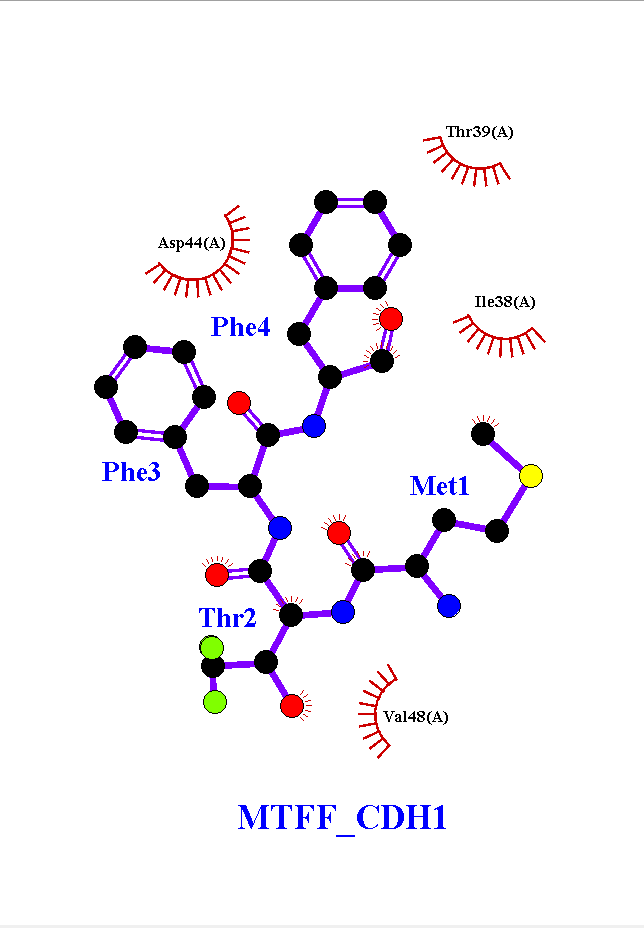

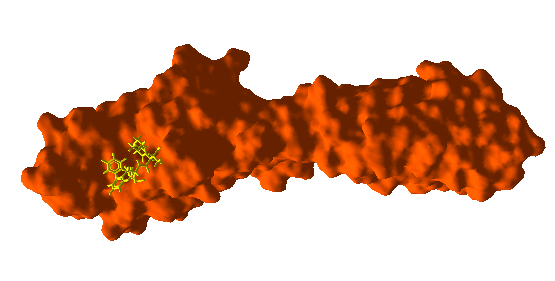


**Figure (4) MTFF_CDH1** Corresponding atoms and non-ligand Val48, Ile38, Thr39 and Asp44 involved in hydrophobic interactions.


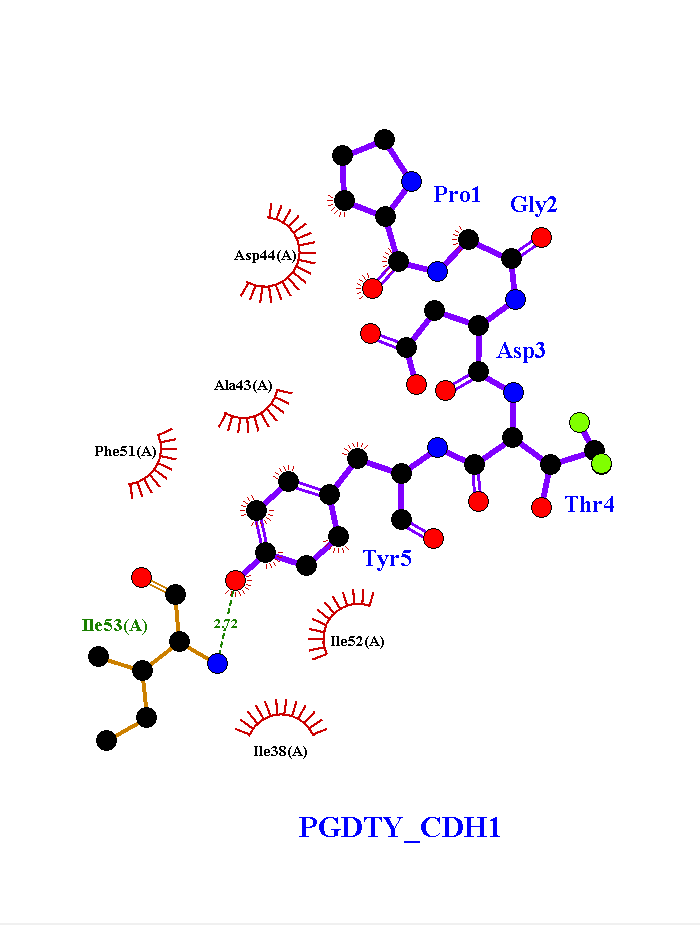

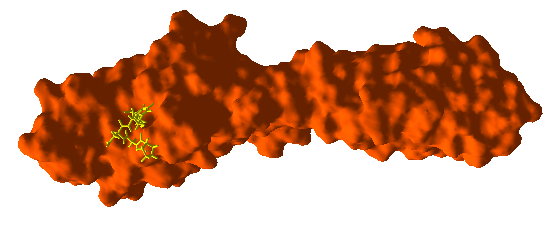


**Figure (5) PGDTY_CDH1** Non-ligand bond. Corresponding atoms and non-ligand Ile52, Ile38, Phe51, Ala43 and Asp44 involved in hydrophobic interactions. Hydrogen bonds between PGDTY and Ile53 shown by dotted green lines.


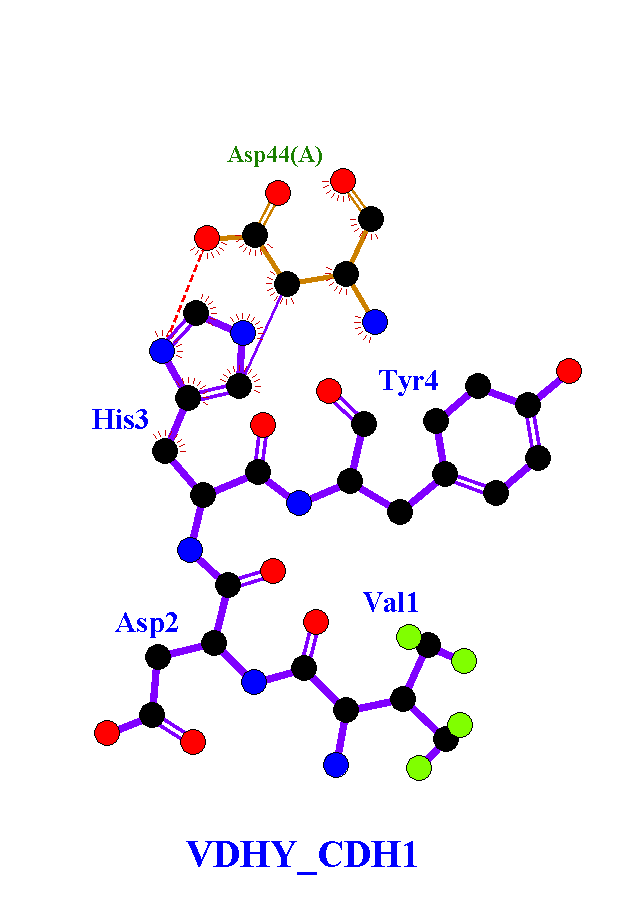

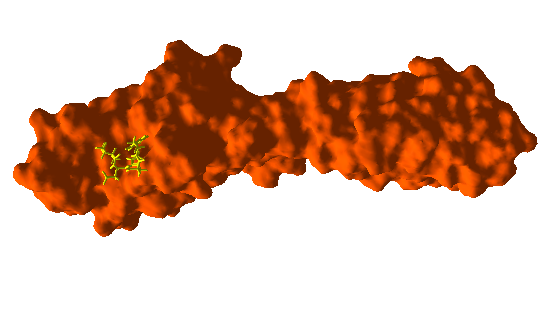


**Figure (6) VDHY_CDH1** Non-ligand bond. External bonds and salt bridges between VDHY and Asp44 and shown by dotted red lines.


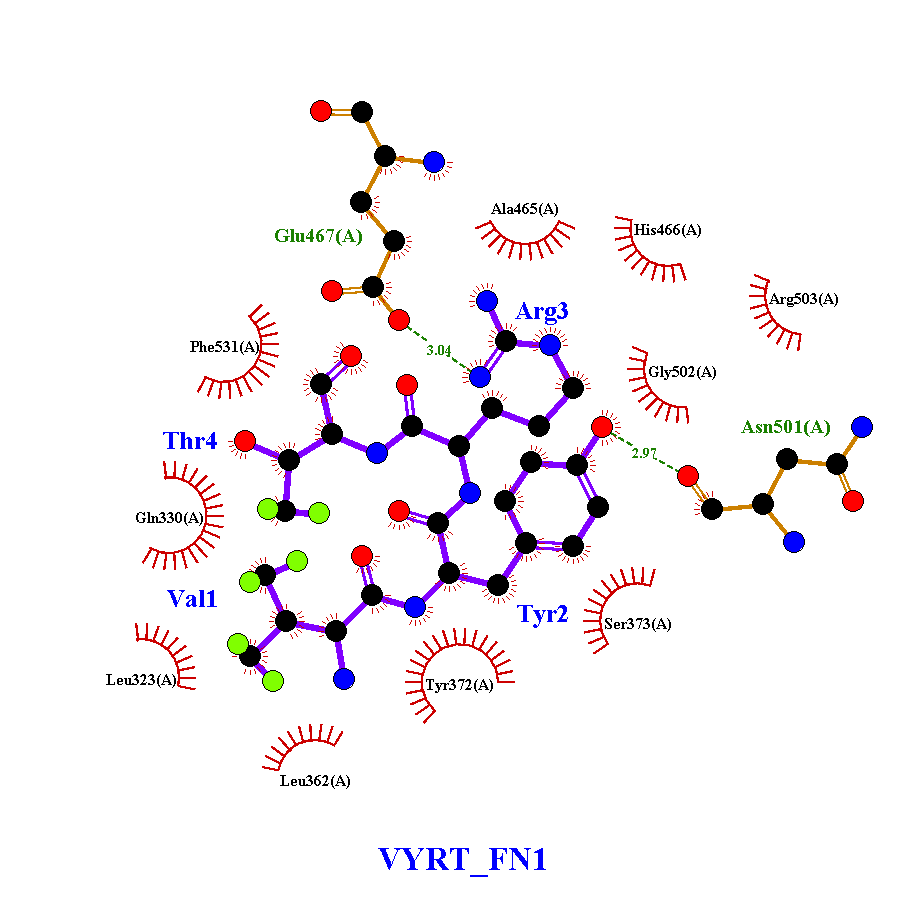

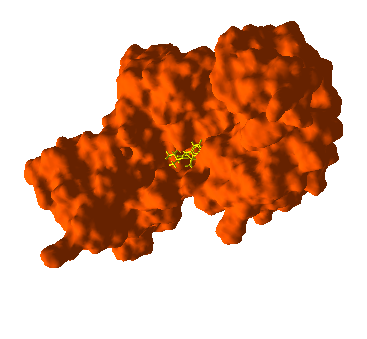


**Figure (1) VYRT_FN1** Non-ligand bond. Corresponding atoms and non-ligand Ser373, Tyr372, Leu362, Leu323, Gln330, Phe531, Phe531, Ala465, His466, Arg503 and Gly502 involved in hydrophobic interactions. Hydrogen bonds between VYRT and Asn501 and Glu467 shown by dotted green lines.


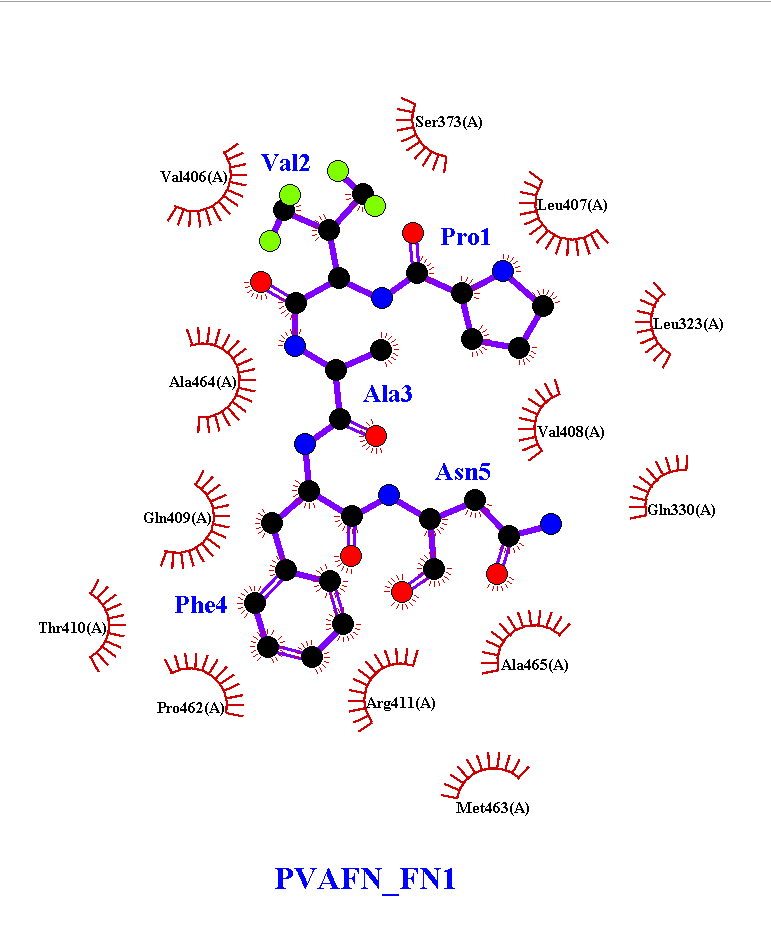

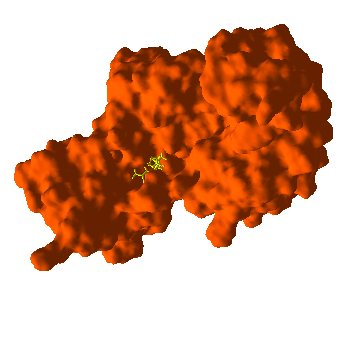


**Figure (2) PVAFN_FN1** Corresponding atoms and non-ligand Arg411, Met463, Ala465, Gln330, Val408, Leu323, Leu407, Ser373, Val406, Ala464, Gln409, Thr410 and Pro462 involved in hydrophobic interactions.


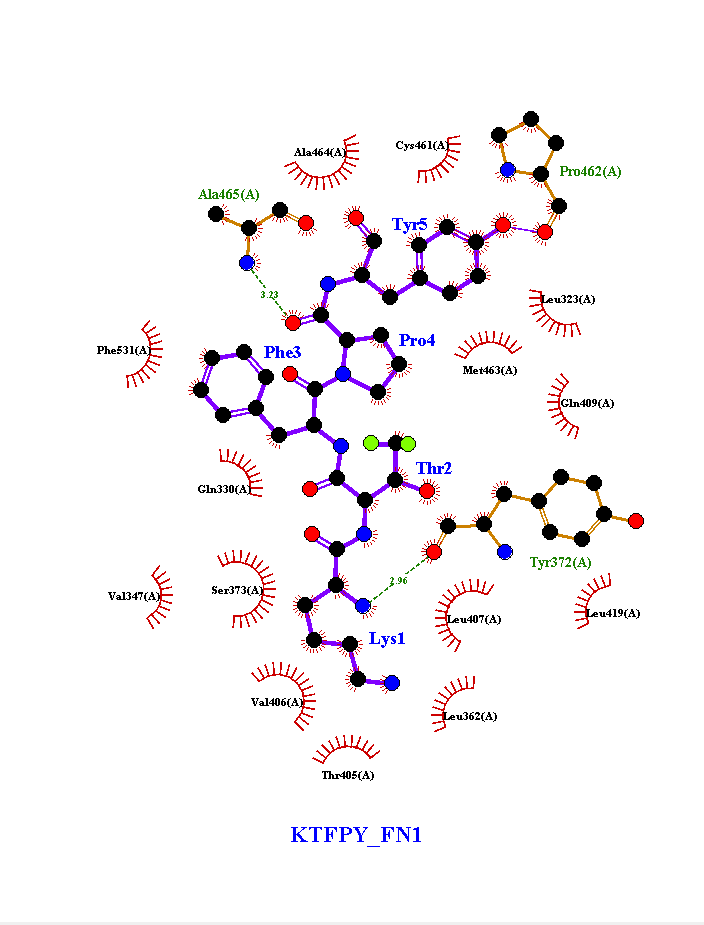

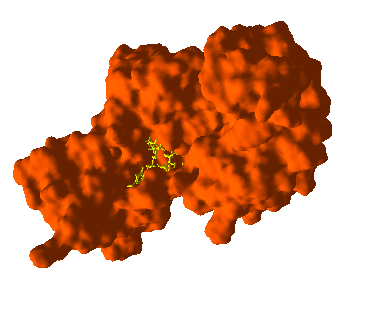


**Figure (3) KTFPY_FN1** Non-ligand bond. Corresponding atoms and non-ligand Leu419, Leu407, Leu362, Thr405, Val406, Ser373, Val347, Gln330, Phe531, Ala464, Cys461, Leu323, Met463 and Gln409 involved in hydrophobic interactions. Hydrogen bonds between KTFPY and Tyr419, Ala465 and external bond with Pro462 shown by dotted green lines.


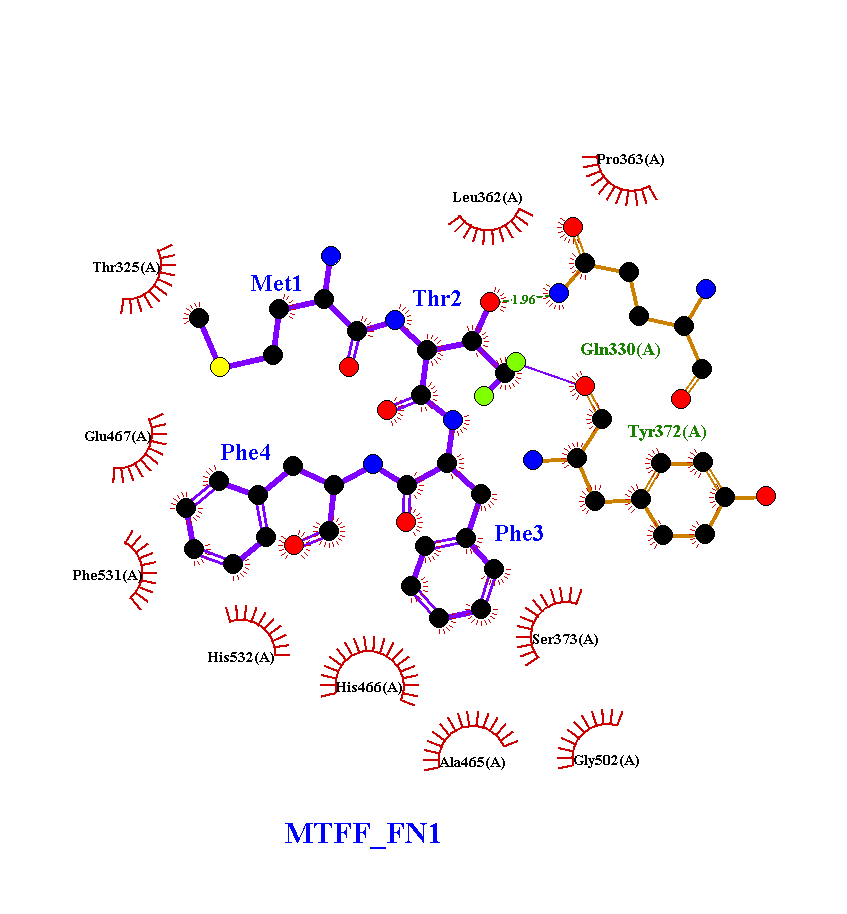

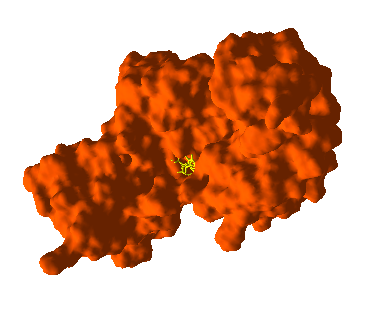


**Figure (4) MTFF_FN1** Non-ligand bond. Corresponding atoms and non-ligand Ser373, Gly502, Ala465, His466, His532, Phe531, Glu467, Thr325, Leu362 and Pro363 involved in hydrophobic interactions. Hydrogen bonds between MTFF and Gln330 and external bond with Tyr372 shown by dotted green lines.


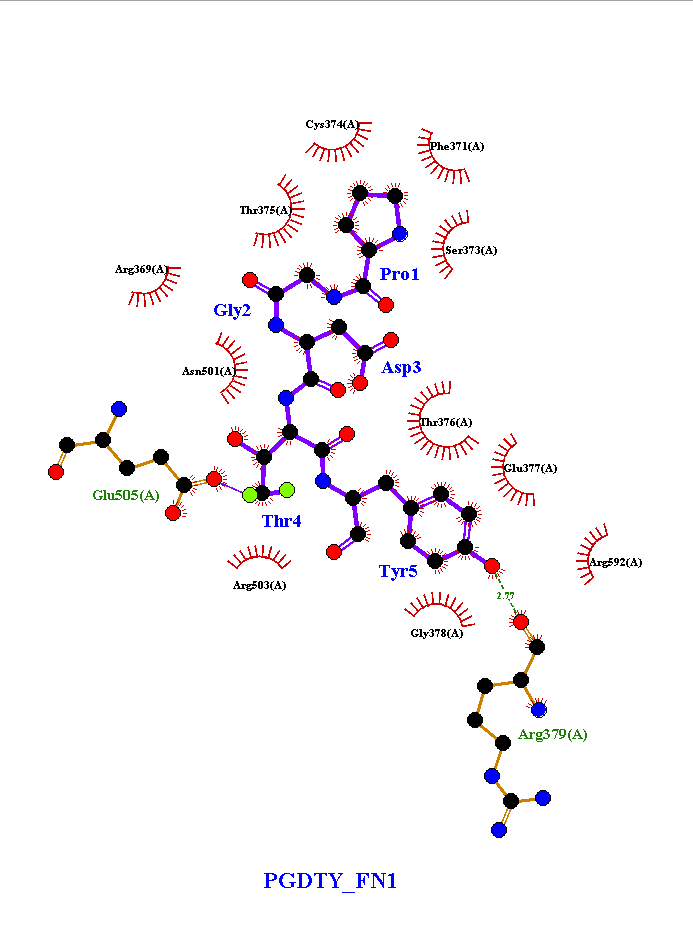

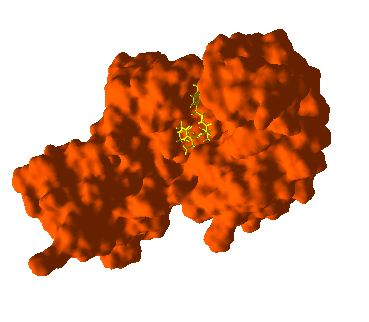


**Figure (5) PGDTY_FN1** Non-ligand bond. Corresponding atoms and non-ligand Arg592, Glu377, Thr376, Ser373, Phe371, Cys374, Thr375, Arg369, Asn501, Arg503 and Gly378 involved in hydrophobic interactions. Hydrogen bonds between PGDTY and Arg379 and external bond with Glu505 shown by dotted green lines.


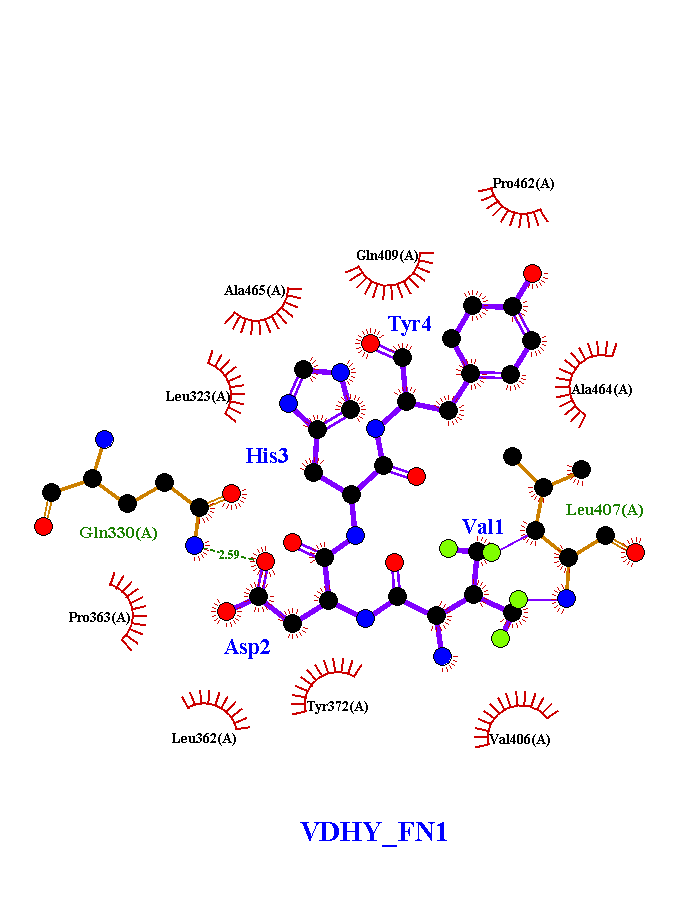

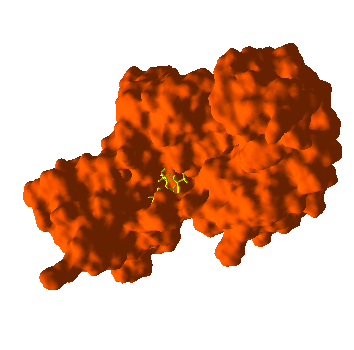


**Figure (6) VDHY_FN1** Non-ligand bond. Corresponding atoms and non-ligand Val406, Tyr372, Leu362, Pro363, Leu323, Ala465, Gln409, Pro462 and Ala464 involved in hydrophobic interactions. Hydrogen bonds between VDHY and Gln330 and external bond with Leu407 shown by dotted green lines.

**Figure (1) Smallpeptide_FN1** Glu20, Glu16, Ala9, Leu8 and Gln5, Gln301, Asn414, Asn416, Gly413, Arg411, Gly417 and Gln409.

**Figure (1) VYRT_STAT3 N**on-ligand bond. Corresponding atoms and non-ligandSer24, Arg107, Ile104, Arg103, Gln17, Gln20 and Tyr94, Lys97 and Arg93 involved in hydrophobic interactions. Hydrogen bonds between VYRT and Glu100 and Asp24 shown by dotted green lines.

**Figure (2) PVAFN_STAT3 N**on-ligand bond. Corresponding atoms and non-ligand Arg70, Phe71, Gln67, Pro36, Ser40, Gln41, Glu63, Phe33, Leu34, Ala35, Ile38 and Asp42 involved in hydrophobic interactions. Hydrogen bonds between VYRT and Glu100 and Asp24 shown by dotted green lines.

**Figure (3) KTFPY_STAT3 N**on-ligand bond. Corresponding atoms and non-ligand Ile64, Leu60, Leu34, Trp37, Gln41, Ser40, Glu39, Pro36, Phe33, Asp42, Phe71 and Arg70 involved in hydrophobic interactions. Hydrogen bonds between KTFPY and Gln66 and external bond with Glu63 shown by dotted green lines.

**Figure (4) MTFF_STAT3 N**on-ligand bond. Corresponding atoms and non-ligand Tyr79, Phe33, Phe71, Asp42, Gln66, Trp37, Leu34, Ala35, Pro36 and Gln32 involved in hydrophobic interactions. Hydrogen bonds between MTFF and Gln67, and Arg70 shown by dotted green lines.

**Figure (5) PGDTY_STAT3** Corresponding atoms and non-ligand Asp24, Ser23, Ser5, Leu21, Gln20, Glu111, Arg114, Gln17, Arg103, Cys108, Ile104, Arg107 and Arg107 involved in hydrophobic interactions.

**Figure (6) VDHY_STAT3 N**on-ligand bond. Corresponding atoms and non-ligand Pro36, Gln67, Gln32, Phe33, Gln66, Asp42, Ser40 and Arg70 involved in hydrophobic interactions. External bonds between VDHY and Gln67, and Arg70 shown by dotted purple lines.

**Figure (1) KTFPY_ACE** Non-ligand bond. Corresponding atoms and non-ligand Tyr287, Leu375, Lys49, Met299, Asn285, Ser284, Thr302, Thr171, Ala170 and Ile286 involved in hydrophobic interactions. Hydrogen bond between KTFPY with Thr301 and external bonds between VDHY and Asp288 and Arg173 and salt bridge with Asp300 shown by dotted purple lines.

**Figure (2) MTFF_ACE** Non-ligand bond. Corresponding atoms and non-ligand Asp288, Arg173, Ser298, Asp300, Ser298, Tyr287, Glu376, Asn285, Ser284, Leu375, Ala170, Thr171 and Asn167 involved in hydrophobic interactions. Hydrogen bond between MTFF with Thr302 shown by dotted green lines.

**Figure (3) PGDTY_ACE** Non-ligand bond. Corresponding atoms and non-ligand Ser222, Pro227, Thr226, Asp218, Tyr213, Lys117 and Glu225 involved in hydrophobic interactions. Hydrogen bond between PGDTY with Arg221 shown by dotted green lines.

**Figure (4) PVAFN_ACE** Non-ligand bond. Corresponding atoms and non-ligand Asn374, Arg173, Aal170, Thr301, Asp300, Ser298, Met299, Asp288, Tyr287, Ser284, Glu376 and Leu375 involved in hydrophobic interactions. Hydrogen bond between PVAFN with Asn285 and Glu303 and external bond with Thr302 shown by dotted green lines.

**Figure (5) VDHY_ACE** Non-ligand bond. Corresponding atoms and non-ligand Thr171, Asn285, Arg173, Ser284, Ser298, Tyr287, Lys449, Thr301, Leu375 and Asn167 involved in hydrophobic interactions. Hydrogen bond between VDHY with Thr302 and external bond with Aa170 and salt bridges with Asp300 shown by dotted green lines.

**Figure (6) VYRT_ACE** Corresponding atoms and non-ligand Asn300, Leu375, Glu376, Lys449, Asp453, Ser284, Thr300, Asn167, Aal170, Asn285, Thr171, Arg173 Asp288 and Tyr287 involved in hydrophobic interactions.

**Angiotensin1-ACE** Corresponding atoms and non-ligand Tyr4, His9, Leu10, Phe8, Pro7, Val3, Arg2, His6 and Asn105, Gly212, Tyr213, Asp121, Ser222, Lys118, Lys117, Val104, Glu225 and Pro227 involved in hydrophobic interactions.

**Figure (2) Angiotensin2-ACE** Non-ligand bond. Corresponding atoms and non-ligand Lys118, Lys117, Tyr213, Asp121, Pro227, Glu225, Thr25 and Thr226 involved in hydrophobic interactions.

**Figure (1) KTFPY_AT1R** Non-ligand bond. Corresponding atoms and non-ligand Glu191, Ile190, Trp99, Glu138, Ser97, Arg96, Thr186, Pro185 and Leu117 involved in hydrophobic interactions. Hydrogen bond between KTFPY with Ile189 and external bond with Asp118 and Ile120 shown by dotted green lines.

**Figure (2) MTFF_AT1R** Non-ligand bond. Corresponding atoms and non-ligand Leu254, Arg210, Trp216, Leu209, Arg214, Glu212 and Arg213 involved in hydrophobic interactions. External bond between MTFF with Ser211 shown by dotted purple lines.

**Figure (3) PGDTY_AT1R** Non-ligand bond. Corresponding atoms and non-ligand Val359, Arg234, Lys232, Gln229, Tyr226, Ala225, Gln350, Asn231 and Lys307 involved in hydrophobic interactions. Hydrogen bond between PGDTY with Lys354 and external bond with Asp321, Ser320 and Ile323 shown by dotted green lines.

**Figure (4) PVAFN_AT1R** Non-ligand bond. Corresponding atoms and non-ligand Gln350, Ile323, Tyr226, Pro233, Lys232, Asn231, Arg234, Ser320, Lys307 and Asp321 involved in hydrophobic interactions. Hydrogen bond between PVAFN with Lys354 and external bond with Val359 shown by dotted green lines.

**Figure (5) VDHY_AT1R** Non-ligand bond. Corresponding atoms and non-ligand Lys354, Arg234, Gln350, Pro233, Asn231, Lys232, Ile323, Asp321 and Lys307 involved in hydrophobic interactions. External bond between VDHY and Val359 shown by dotted green lines.

**Figure (6) VYRT_AT1R** Non-ligand bond. Corresponding atoms and non-ligand Ser211, Arg213, Glu212, Thr47, Gly46, Gly207, Leu254, Leu45 and Val206 involved in hydrophobic interactions. Hydrogen bond between VYRT with Arg210 and Leu4 and external bond VYRT with Arg210 and salt bridges with Asp205 shown by dotted green lines.

**Figure (2) Angiotensin2_AT1R** Non-ligand bond. Corresponding atoms and non-ligand Ser334, Arg30, Glu26, Thr87, Asn22, Asp23, Lys89, Arg52, Lys33, Phe335 and Leu55 involved in hydrophobic interactions. External bond ANG2 with His54 shown by dotted green lines.

**Figure (1) KTFPY_AT2R** Non-ligand bond. Corresponding atoms and non-ligand Trp1007, Asn1011, Lys1015, Asp1012, Glu1008, Pro1046, Leu1003, Glu1004, Leu1106 and Thr1101 involved in hydrophobic interactions. Hydrogen bond between KTFPY with Arg1098 and external bond with Ile102 and salt bridges with Asp205 shown by dotted green lines.

**Figure (2) MTFF_AT2R** Corresponding atoms and non-ligand Glu1004, Leu1106, Ile1102, Trp1007, Glu1008, Asp1012, Asn1011, Arg1098 and Lys1015 involved in hydrophobic interactions.

**Figure (3) PGDTY_AT2R** Non-ligand bond. Corresponding atoms and non-ligand Glu1057, Lys1104, Tyr1105, Ser901, Ser40, Gly902, Pro39 and Gln1103 involved in hydrophobic interactions. Salt bridges between PGDTY and Lys42 shown by dotted red lines.

**Figure (4) PVAFN_AT2R** Non-ligand bond. Corresponding atoms and non-ligand Glu1008, Asp1012, Arg1098, Asn1011, Lys1015, Glu1004, Leu1106, Tyr1104 and Trp1007 involved in hydrophobic interactions. External bonds PVAFN with Ile1102 shown by dotted purple lines.

**Figure (5) VDHY_AT2R** Non-ligand bond. Corresponding atoms and non-ligand Lys1015, Asp1012, Glu1008, Glu1004, Leu1106, Trp1007, Tyr1101 and Arg1098 involved in hydrophobic interactions. External bonds between VDHY with Asn1011 and Ile1102 shown by dotted purple lines.

**Figure (6) VYRT_AT2R** Non-ligand bond. Corresponding atoms and non-ligand Pro39, Gly902, Ser901, Cys35, Glu1057 and Ser40 involved in hydrophobic interactions. Salt bridges between and Asp41 shown by dotted red lines.

**Figure (1) Angiotensin2_AT2R** Non-ligand bond. Corresponding atoms and non-ligand Asn1006, Ala1036, Ala1035, Thr1031, Lys1032, Asn1013, Glu1008, Asp1012 and Asp1005 involved in hydrophobic interactions. External bonds between AT2R and Thr1009 and Ala100 shown by dotted purple lines.

**Figure (1) KTFPY_APOE** Non-ligand bond. Corresponding atoms and non-ligand Lys146, Arg38, Arg145, Trp34, Leu149, Asp153 and Lys156 involved in hydrophobic interactions. Hydrogen bond between KTFPY with Asp154 and external bond with Arg150 shown by dotted green lines.

**Figure (2) MTFF_APOE** Non-ligand bond. Corresponding atoms and non-ligand Asp153, Leu149, Ala152, Leu148, Leu38, Trp34, Gly31 and involved in hydrophobic interactions. Hydrogen bond between MTFF with Gln156 and external bond with Gln156 shown by dotted green lines.

**Figure (3) PGDTY_APOE** Non-ligand bond. Corresponding atoms and non-ligand Leu30, Gly31, Glu27, Gly23, Trp34, Leu149, Ala152, Trp26, Asp153 and Lys157 and involved in hydrophobic interactions. Hydrogen bond between PGDTY with Gln156 and external bond with Gln156 shown by dotted green lines.

**Figure (4) PVAFN_APOE** Non-ligand bond. Corresponding atoms and non-ligand Met64, Met68, Glu66, Lys72 and Lys69 involved in hydrophobic interactions. Hydrogen bond between PVAFN with Asp65 shown by dotted green lines.

**Figure (5) VDHY_APOE** Corresponding atoms and non-ligand Ala152, Gln156, Glu27, Leu28, Gly31, Asp35, Trp34, Leu30 and Leu149 involved in hydrophobic interactions.

**Figure (6) VYRT_APOE** Non-ligand bond. Corresponding atoms and non-ligand Lys146, Arg145, Arg142, Lys143, Trp34 and Leu149 involved in hydrophobic interactions. Hydrogen bond between VYRT with Arg38 and Arg150 shown by dotted green lines.

**Figure (1) KTFPY_CD4** Non-ligand bond. Corresponding atoms and non-ligand Lys7, Glu169, Lys171, Ile172, Gly93, Thr101, Ala102, Thr106, Asp105, Lys8 and Asp10 involved in hydrophobic interactions. Hydrogen bond between KTFPY with Phe98, Leu100 and Gly9 shown by dotted green lines.

**Figure (2) MTFF_CD4** Corresponding atoms and non-ligand Ile172, Asp10, Gly99, Lys7, Phe98, Phe170, Leu100, Lys8, Lys171, Ala192, Thr106 and Asp173 involved in hydrophobic interactions.

**Figure (3) PGDTY_CD4** Non-ligand bond. Corresponding atoms and non-ligand Glu77, Pro122, Ser79, Asp80, Ser31, Asp80, Tyr82, Ile34 and Ser49 involved in hydrophobic interactions. Hydrogen bond between PGDTY with Asn30 and external bonds with Asn30 and Leu51 and salt bridges with Lys50 shown by dotted green lines.

**Figure (4) VDHY_CD4** Corresponding atoms and non-ligand Ile34, Asp80, Asn30, Asp78, Glu77, Lys59, Ser49, Tyr82 and Leu51 involved in hydrophobic interactions.

**Figure (5) PVAFN_CD4** Non-ligand bond. Corresponding atoms and non-ligand Thr106, Asp173, Lys171, Ile172, Leu100, Asp10, Lys7, Lys8, Thr101, Phe98 and Gly99 involved in hydrophobic interactions. External bonds Between PVAFN and Phe170 shown by dotted purple lines.

**Figure (6) VYRT_CD4** Non-ligand bond. Corresponding atoms and non-ligand Arg54, Pro122, Ser79, Asn32, Ile34, Asn38, Ser49, Asp78 and Leu51 involved in hydrophobic interactions. Hydrogen bond between VYRT with Glu77, Asp80, Lys75 and Lys50 shown by dotted green lines.

**Figure (1) VYRT_CD68** Corresponding atoms and non-ligand Ser150, Asn197, Ser153, Leu196, Lys154, Ser148, Pro149, Pro149, Thr156, Pro198, Ie193, Val195, Pro149 involved in hydrophobic interactions.

**Figure (2) PVAFN_CD68** Non-ligand bond. Corresponding atoms and non-ligand Ser150, Ser194, Ser148, Pro147, Ser146, Pro219, Lys200, Asn197, Tyr220, Ser153, Lys154, Thr156 and Leu196 involved in hydrophobic interactions. External bond between P34810_11855 with Val195 shown by dotted green lines.

**Figure (3) KTFPY_CD68** Non-ligand bond. Corresponding atoms and non-ligand Thr201, Phe218, Lys200, eu196, Pro219, Tyr200, Pro147, Ser148, Ser153, Val195, Thr156, Lys154, Glu155 and Gln175 involved in hydrophobic interactions. External bond between P34810_14051 with Asn197 shown by dotted green lines.

**Figure (4) MTFF_CD68** Corresponding atoms and non-ligand Tyr220, Pro219, Lys200, Phe218, eu196, Asn197, Val195, Pro198, Ser153 and Ser150 involved in hydrophobic interactions.

**Figure (5) PGDTY_CD68** Corresponding atoms and non-ligand His211, Phe227, Pro212, Gly226, Met228, Ser239, Leu264, Arg265, Tyr237, Ala262 and Tyr240 involved in hydrophobic interactions.

**Figure (6) VDHY_CD68** Corresponding atoms and non-ligand SER239, His213, His211, Glu208, Pro212, Phe227, Gly226, Tyr240 and Ala262 involved in hydrophobic interactions.
