# Supplementary material for: The anti-aging potential of antihypertensive peptides of Pariset, a dataset of algal peptides
Source: Front Aging. 2025 Jul 30;6:1618082. doi: 10.3389/fragi.2025.1618082 (PMC12343563; doi:10.3389/fragi.2025.1618082)
Supplement: Supplementary file 3 [file Table3.docx]

**The anti-aging potential of antihypertensive peptides of *Pariset*, a dataset of algal peptides**

**Isaac Karimi^1^*, Parisa Olfati^1^, Layth Jasim Mohammed^2^, Jawad Kadhim Tarrad^2^, Ahmed M. Amshawee^3^, Maryam A. Hussain^4^ and Helgi B. Schiöth*^5^**

^1^Laboratory for Computational Physiology, Department of Biology, Faculty of Science, Razi University 67149-67346, Kermanshah, Iran. p.olfati1999@gmail.com

^2^Department of Microbiology, College of Medicine, Babylon University, Hilla City, Babylon Governorate, 51002, Iraq. E-mail: [med996.layth.jasim@uobabylon.edu.iq](mailto:med996.layth.jasim@uobabylon.edu.iq)

^3^Department of Radiology, University of Hilla, Babylon, Iraq. E-mail: [ahmed_meki@hilla-unc.edu.iq](mailto:ahmed_meki@hilla-unc.edu.iq)

^4^Babylon Technical Institute, AL-Furat Al-Awsat Technical University, Babylon, Iraq. E-mail: maryam.hussein.iba3@atu.edu.iq

^5^Department of Surgical Sciences, Functional Pharmacology and Neuroscience, Uppsala University, 751 24, Uppsala, Sweden.

*Correspondence: Helgi B. Schiöth, helgi.schioth@uu.se, Tel and Fax: 0046-18-4714160; Isaac Karimi; isaac_karimi2000@yahoo.com; karimiisaac@razi.ac.ir. Tel & Fax: 0098-83-34274545.

Table 1. *In silico* molecular docking of top-list aging targets with angiotensin-converting enzyme inhibitors presented as binding affinity (upper bound, lower bound)

| Ligand | Protein targets | | | | | | | | | | | | | | |
| --- | --- | --- | --- | --- | --- | --- | --- | --- | --- | --- | --- | --- | --- | --- | --- |
| PubChem code / Name | 1L-1RA | ACE | APOE | AT1R | AT2R | CD4 | CD8A | CD44 | CD45 | CD68 | CDH1 | FN1 | IL1B | ITGAM | STAT3 |
| 44093  Captopril | -5.6 (2.92; 1.864) | -6.7 (23.268; 22.218) | -5.3 (3.623; 2.542) | -5.5 (51.467; 50.879) | -5.0 (2.703; 2.176) | -5.5 (11.459; 10.572) | -5.5 (11.459; 10.572) | -5.8 (12.299; 11.125) | -6.6  93.681; 2.19) | -5.6 (3.246; 1.872) | -5.5 (13.514; 12.889) | -5.5 (2.128, 1.565) | -5.4 (3.616; 2.521) | -6.2 (1.53; 1.264) | -4.9 (26.806; 26.244) |
| 54892  Quinapril | -7.9 (3.04; 1.771) | -8.6 (6.446; 3.249) | -7.0 (4.11; 2.535) | -9.1 (3.464; 1.603) | -7.4 (4.62; 2.096) | -7.9 (5.132; 2.076) | -7.9 (5.132; 2.076) | -8.6 (2.488; 1.52) | -8.3 (71.433; 68.745 | -9.0 (2.525,1.361) | -6.9 (13.29;10.48) | -8.8 (2.059; 1.491) | -7.4 (25.691; 23.734) | -7.3 (4.967; 1.103) | -7.8 (4.123; 2.298) |
| 91270  Moexipril Hydrochloride | -7.8 (4.42; 1.932) | -8.9 (4.735; 2.115) | -6.7 (1.387; 1.115) | -8.1 (3.026; 1.51) | -6.7 (5.067; 2.311) | -7.5 (5.479; 1.836) | -7.5 (5.479; 1.836) | -7.7 (14.457; 11.903) | -7.7 (28.556; 26.887) | -8.5 (2.941; 1.195) | -7.7 (4.131;1.888) | -8.3 (5.071;3.154) | -7.3 (1.67; 1.292) | -7.4 (12.16; 9.996) | -6.9 (3.781; 2.361) |
| 107807  Perindopril | -6.6 (24. 991; 21.903) | -7.7 (5.354; 3.202 | -6.3 (4.419; 2.609) | -7.8 (2.932; 2.369) | -5.9 (4.211; 2.324) | -6.5 (15.573; 12.955) | -6.5 (15.573; 12.955) | -7.1 (2.766; 1.582) | -6.6 (1.999; 0.752) | -7.5 (4.4; 1.124) | -5.8 (28.6; 26.93) | -6.7 (2.077;0.86) | -6.5 (2.013; 0.687) | -6.6 (34.753; 32.443) | -6.3 (5.425; 2.115) |
| 5362129  Ramipril | -7.2 (2.24; 1.601) | -8.4 (6.132; 3.707) | -6.6 (15.613; 13.232) | -7.7 (2.793; 2.175) | -7.0 (6.43; 2.195) | -7.2 (21.645; 18.359) | -7.2 (21.645; 18359) | -7.6 (14.922; 12.095) | -7.9 (4.048; 2.018) | -8.2 (5.894; 2.416) | -7.5 (2.119;1.354) | -7.4 (4.636;3.094) | -7.0 (0.777; 0.429) | -7.2 (3.126; 2.08) | -6.6 (4.649; 3.423) |
| 5388962  Enalapril | -7.1 (16.981; 14.123) | -7.7 (5.556; 2.016) | -6.8 (1.915; 1.288) | -7.3 (4.389; 2.818) | -6.4 (5.821; 2.478) | -6.9 (15.742; 14.003) | -6.9 (18.033; 15.381) | -7.3 (14.285; 12.413) | -8.7 (5.822; 2.253) | -6.2 (6.667; 3.247) | -5.9 (5.507;1.779) | -7.0 (34.095;33.206) | -6.4 (4.566; 3.11) | -7.1 (5.51; 1.832) | -6.8 (3.315; 0.891) |
| 5484727  Trandolapril | -7.3 (4.38; 2.349) | -8.4 (29.329; 26.61 | -6.6 (6.698; 3.131) | -7.0 (5.009; 2.124) | -7.1 (6.42; 2.229) | -7.6 (15.459; 13.474) | -7.6 (15.459; 13.474) | 7.7 (14.623; 12.297) | -7.7 (73.366;70.19 | -8.1 (4.068; 2.36) | -7.6 (2.28; 1.357) | -7.5 (4.627; 2.978) | -7.1 (5.413; 1.901) | -6.8 (4.69;1.168) | -7.3 (5.67; 3.957) |
| 53562119  Lisinopril | -6.5 (37.771; 35.824) | -8.1 (4.865; 2.013) | -5.7 (5.465; 2.827) | -6.7 (59.108; 56.5) | -5.7 (32.864; 31.517) | -6.6 (13.356; 10.938) | -6.6 (13.356; 10.938) | -6.6 (3.796; 2.64) | -7.0 (30.913; 28.955) | -7.2 (5.001; 2.267) | -5.6 (6.748; 5.065) | -7.6 (16.507;13.3) | -6.5 (5.325; 1.885) | -7.3 (2.884; 1.529) | -6.3 (5.388;1.804) |
| 53562124  Benazepril | -7.6 (27.958; 24.947 | -8.5 (3.508; 1.586) | -8.1 (4.863; 1.66 | -8.2 (6.266; 2.105) | -6.8 (31.774; 29.347) | -7.5 (1.291; 1.083) | -7.5 (1.291; 1.083) | -8.2 (3.344; 0.856) | -8.1 (113.153; 110.147) | -9.0 (3.241; 1.522) | -7.7 (4.484; 2.157) | -8.0 (2.5; 1.624) | -7.2 (4.146; 2.666) | -7.6 (5.775; 1.445) | -7.2 (3.373; 1.271) |
